# Supplementary material for: Care seeking for under-five children and vaccine perceptions during the first two waves of the COVID-19 pandemic in Lagos State, Nigeria: a qualitative exploratory study
Source: BMJ Open. 2023 Mar 7;13(3):e069294. doi: 10.1136/bmjopen-2022-069294 (PMC10008198; doi:10.1136/bmjopen-2022-069294)
Supplement: Supplementary data [file bmjopen-2022-069294supp001.pdf]

Appendix I: -Ease of COVID – 19 Lockdown in Nigeria

	Phase 1	Phase 2	Phase 3	Phase 4	Phase 5
**Start date**<br><br>**End date**	4th May 2020<br><br>1st June 2020	2nd June 2020<br><br>18th October 2020	19th October 2020	11th May 2021	2nd April 2022
**Land travel (Interstate)**	Banned except for essential services and movement of goods and services only	Open under strict conditions: Allowed for essential services and movement of goods and services only	Open	Open	Open
**Land travel (Intrastate)**	Limited to 6 am-6 pm with a 50% reduction in bus occupancy	Open	Open	Open	Open
**Airspace**	Closed for most passenger flights. Open to cargo and specially approved flights only	Open for domestic flights, limited for essential international flights until August 26	Open for domestic and international flights	Open for domestic and international flights	Open for domestic and international flights
**Movement**	Curfew from 8pm to 6am	Curfew from 10pm – 4am	12am to 4am	Curfew from 12am – 4am	No restrictions
**Working hours**	9 am to 2 pm	9 am – 2 pm for Government/other corporate offices	All government staff on grade level 12 and below to continue staying at home<br><br>No limit for private and other corporate bodies	All government staff on grade level 12 and below to continue staying at home until 11th June 2021<br><br>No limit for private and other corporate bodies	No restrictions
**Workspace**	50% staff occupancy or less	75% staff occupancy or less<br>50% for clients	100% occupancy	No limits but virtual meetings and work from home encouraged	No restrictions
**Entertainment activities**	Banned	Banned	Open	Open with some restrictions (bars, night clubs, pubs remained closed)	Open at 50% capacity
**Mass gathering**	Limited to 20 people or less	Limited to 20 people or less	Limited to 50 people or less	Limited to 50 people or less except with permission	Open

				from the state government	
**Religious gathering**	Restricted	Restricted	Restricted (subject to the protocol from the state government and the federal capital territory	Limited to less than 50% capacity<br><br>Gathering more than 50 people must be held outdoors only	Open
**Schools**	Closed	Closed, but special consideration for graduation exams	Open	Open	Open
**Markets**	Partial closure (opened only on designated days weekly between 8.00 am-3.00 pm)	Controlled access by local authorities	Open	Open	Open
**Face masks**	Mandatory for all persons in public spaces	Mandatory for all persons in public spaces	Mandatory for all persons in public spaces	Mandatory for all persons in public spaces	Mandatory for indoor activities only, but at individual discretion for outdoor activities
**Banks and other financial institutions**	Limited staff physically to between 30%-50%	Limited staff physically to 75% or less. Operated for normal working hours	Open	Open	Open

Source: NCDC Coronavirus COVID-19 Microsite. Accessed June 29, 2022. https://covid19.ncdc.gov.ng/guideline/

- *There was a total lockdown of economic activities in the FCT, Lagos and Ogun states for 35days from 30th March 2020. This was coupled with a total ban on non-essential interstate travels*
- *From the third phase, the end dates were assumed as the onset of the next phase*
- *Data collection was done during the phase 3*
- *The second wave of infection and vaccine rollout started during the phase 3*

Appendix II: In-depth interview guide for healthcare provider's interviews

1. Tell me about the facility you work in?
    a. What type of services do you offer children?
    b. Tell me specifically about this week in your clinic
2. Think about last 8 months, has things been typical? Why? Why not?
    a. When did you first hear about covid?
    b. When did you make adaptation or adjustment in your facility as a result of covid?
    c. What changes did your facility make?

NOW WE WANT TO FOCUS ON QUESTIONS REGARDING CHILDREN

3. Tell me how the lockdown in year 2020 affected service provision at your facility
    a. How did it affect services you provide for children?
    a. How did it affect care seeking for sick under-five?
        - Probe severity of illness at presentation/late presentation
        - Was the PHC the first point of call?

4. Thinking about this time last year, before covid/EndSARS, is there any differences? What is different (numbers, type of presentation, services provided, resources), what is the same?
5. Now that lockdown is over, have things normalized the way it used to be before COVID-19? What has normalized? What is yet to normalize. What about number of under-five that you see, any difference compared to last year in terms of number, type of presentation
6. Late in last year, there was Endsars protest. How did it affect service delivery in your facility?
7. Currently, there is second wave of Covid-19 in Nigeria. How has it affected service delivery in your facility?
8. What can you say about the care seeking behavior of caregivers of sick children you have attended to in recent times?
9. Between Covid-19 lockdown, End-Sars protest and current economic hardship, which one has affected care seeking for sick children most? Why? Short term consequences? Any long term consequences?
10. Finally, the federal government is making plans to procure Covid-19 vaccines for Nigerians. How willing are you to receive the vaccine? Why/why not? What about for your child/children? Why/why not? Will you tell others to take it? Why?
11. Do you have any other things to say?

Appendix III: In – Depth interview guide for caregiver of under-five with recent illness episode

1. Tell me about your family.
   Probe to get information on:
   a. Who lives with the participant
   b. Participant's job
   c. Where participant's extended family live
   d. Involvement in child's care
2. How will you summarize year 2020?
   Probe:
   a. How did it affect you and your family?
   b. Could you say these changes were due to impact of covid-19 pandemic?
      1. If yes, why? In what other ways have covid-19 affected you and your household
      2. If no, why not?
   c. Have you noticed changes in the price of commodities?
      i. How does this affect you and your household?
         1. House rent
         2. Transport cost
         3. Food items

Now we want to talk about child health services, particularly care seeking for sick under-five

3. Your child was recently sick; I would like to know more about the illness.
   a. How did it start? Who first noticed the symptoms?
   b. What did you do first? When did you do that?
   c. What next did you do?
      i. How did you decide?
      ii. Why did you do that? Could you have done something else?
      iii. What treatment were given? Was your child asked to do some tests? Could you afford all the test?
      iv. Were you referred?
         1. If yes, did you honour the referral? Why?
         2. How did you feel with the referral?
         3. If no, why? What did you do next? Why did you do that?
         4. Was your child asked to do some tests? Could you afford all the test?
         5. What about medications? Did you buy all the medication?
      v. Like how much did it cost you to treat your child? Would the cost have been cheaper if not for current situations? How did you cover the cost of treatments for your child?
         1. Personal money/savings?
         2. Support from father?
         3. Support family and friends?
         4. Did you have to borrow or sell any items?

4. Overall, has covid-19 affected your decisions and steps when your child was sick?
   i. If yes, how?
   ii. If no, what affected your decisions and steps?
      i. Endsars protest?

ii. Insecurity?
iii. Current economic hardship?
iv. Could you have taken different actions/steps (*relate this to previous answers*) What about fears of catching covid at the hospital?

5. Finally, the federal government has indicated that by Jan 2021 the country will have covid-19 vaccine. How willing are you to receive the vaccine? Why/why not? What about for your child/children? Why/why not?
6. Do you have other things to say or bring to my attention? Thank you for your time!

Appendix IV: IDI guide for No illness episode

1. Tell me about your family.
   Probe to get information on:
   e. Who lives with the participant.
   f. Participant's job
   g. Where participant's extended family live
   h. Involvement in child's care
2. How will you summarize year 2020?
   Probe:
   a. How has it affected you and your family?
   b. Were these covid-19 related?
      i. If yes, why and how? In what **other** ways have covid-19 affected you and your household
      ii. If no, what do you think is responsible?
   c. Have you noticed changes in the price of commodities?
      i. How does this affect you and your household?
         1. House rent
         2. Transport cost
         3. Food items
         4. Fuel price
3. Now we want to talk about care seeking for under-five. What actions do mothers/caregivers take when their child develops illness?
      i. Why do they do that?
         1. Could it be because of trust/distrust in health care workers?
         2. Could cost have influenced their decision? How?
         3. What else could have influenced their action?
      ii. Think about the last time your child (or that of someone close to you) fell sick
         1. What was wrong? What did you do? How did you decide on what to do? Who did you talk to? What alternatives were considered? What were your concerns?
         2. Was your child referred?
         3. Did you honour the referral?
         4. If yes, why? If no, why?
         5. If it happens this period, could you or they have taken different action? Why?
      iii. During the covid-pandemic in Nigeria, do you think covid-19 affected decisions taken by caregivers when their child was sick? if yes, why and how? If no, why?
      iv. What about now? Do covid-19 affect actions taken by mothers when their child falls sick?
      v. Between covid-19 and current economic hardship, which one has greater influence on actions taken by caregivers when their child is sick?
         1. Why and how?
4. Finally, the federal government has indicated that by Jan 2021 the country will have covid-19 vaccine. How willing are you to receive the vaccine? Why/why not? What about for your child/children? Why/why not?
5. Do you have any other thing to tell me?

Thank you for your time!

Appendix V Outpatient attendance for under-five children in the 7 flagship facilities in Ikorodu LGA (January-June 2020)*

**Year**	**Flagship PHCs**													
2020	**Ikorodu**		**Igbogbo**		**Odonla**		**Agbede**		**Ipakodo**		**Imota**		**Oke-Eletu¥**	
	**Diagnosis**	**Number**	**Diagnosis**	**Number**	**Diagnosis**	**Number**	**Diagnosis**	**Number**	**Diagnosis**	**Number**	**Diagnosis**	**Number**	**Diagnosis**	**Number**
**January—March**	Pneumonia	9	Pneumonia	3	Pneumonia	1	Pneumonia	0	Pneumonia	3	Pneumonia	19	Pneumonia	-
	LRTI	7	LRTI	12	LRTI	1	LRTI	0	LRTI	1	LRTI	2	LRTI	-
	URTI	133	URTI	290	URTI	89	URTI	0	URTI	102	URTI	47	URTI	-
	ARTI	0	ARTI	0	ARTI	0	ARTI	0	ARTI	5	ARTI	0	ARTI	-
	Malaria	511	Malaria	275	Malaria	149	Malaria	129	Malaria	234	Malaria	125	Malaria	-
	Sepsis	43	Sepsis	97	Sepsis	243	Sepsis	21	Sepsis	42	Sepsis	33	Sepsis	-
	Others	274	Others	374	Others	406	Others	115	Others	211	Others	252	Others	-
	**Total**	**977**	**Total**	**1051**	**Total**	**892**	**Total**	**265**	**Total**	**589**	**Total**	**478**	**Total**	**-**
**April—June**	Pneumonia	1	Pneumonia	0	Pneumonia	2	Pneumonia	0	Pneumonia	0	Pneumonia	1	Pneumonia	0
	LRTI	3	LRTI	1	LRTI	0	LRTI	0	LRTI	0	LRTI	0	LRTI	0
	URTI	10	URTI	55	URTI	5	URTI	1	URTI	25	URTI	2	URTI	34
	ARTI	0	ARTI	0	ARTI	0	ARTI	0	ARTI	0	ARTI	0	ARTI	0
	Malaria	215	Malaria	183	Malaria	64	Malaria	23	Malaria	39	Malaria	52	Malaria	42
	Sepsis	22	Sepsis	26	Sepsis	55	Sepsis	2	Sepsis	9	Sepsis	8	Sepsis	63
	Others	113	Others	39	Others	159	Others	5	Others	99	Others	125	Others	164
	**Total**	**364**	**Total**	**304**	**Total**	**285**	**Total**	**31**	**Total**	**172**	**Total**	**188**	**Total**	**303**

*Lagos placed on lockdown on the 30 March 2020

¥ Facility register not found
