# Supplementary material for: Care seeking for under-five children and vaccine perceptions during the first two waves of the COVID-19 pandemic in Lagos State, Nigeria: a qualitative exploratory study
Source: BMJ Open. 2023 Mar 7;13(3):e069294. doi: 10.1136/bmjopen-2022-069294 (PMC10008198; doi:10.1136/bmjopen-2022-069294)
Supplement: Author's manuscript [file bmjopen-2022-069294.draft_revisions.pdf]

BMJ Open

BMJ Open is committed to open peer review. As part of this commitment we make the peer review history of every article we publish publicly available.

When an article is published we post the peer reviewers' comments and the authors' responses online. We also post the versions of the paper that were used during peer review. These are the versions that the peer review comments apply to.

The versions of the paper that follow are the versions that were submitted during the peer review process. They are not the versions of record or the final published versions. They should not be cited or distributed as the published version of this manuscript.

BMJ Open is an open access journal and the full, final, typeset and author-corrected version of record of the manuscript is available on our site with no access controls, subscription charges or pay-per-view fees (http://bmjopen.bmj.com).

If you have any questions on BMJ Open's open peer review process please email info.bmjopen@bmj.com

BMJ Open

Care seeking for under-five children during the first two waves of the COVID-19 pandemic in Lagos State, Nigeria: a qualitative exploratory study

Journal:	*BMJ Open*
Manuscript ID	bmjopen-2022-069294
Article Type:	Original research
Date Submitted by the Author:	23-Oct-2022

Complete List of Authors:	Bakare, Ayobami; Karolinska Institutet, Department of Global Public Health; University College Hospital, Department of Community Medicine<br>Olojede, Omotayo; University College Hospital Ibadan, Department of Paediatrics<br>King, Carina; Karolinska Institute<br>Graham, Hamish; Centre for International Child Health, University of Melbourne, MCRI, Royal Children's Hospital; University College Hospital Ibadan, Department of Paediatrics<br>Uchendu, Obioma; University College Hospital Ibadan, Department of Community Medicine; University of Ibadan College of Medicine, Department of Community Medicine<br>Colbourn, Timothy; UCL Institute for Global Health<br>Falade, Adegoke; University College Hospital Ibadan, Department of Paediatrics; University of Ibadan College of Medicine, Department of Paediatrics<br>Alvesson, Helle; Karolinska Universitetssjukhuset, Global Public Health

Keywords:	COVID-19, Community child health < PAEDIATRICS, PREVENTIVE MEDICINE, PRIMARY CARE

SCHOLARONE™
Manuscripts

I, the Submitting Author has the right to grant and does grant on behalf of all authors of the Work (as defined in the below author licence), an exclusive licence and/or a non-exclusive licence for contributions from authors who are: i) UK Crown employees; ii) where BMJ has agreed a CC-BY licence shall apply, and/or iii) in accordance with the terms applicable for US Federal Government officers or employees acting as part of their official duties; on a worldwide, perpetual, irrevocable, royalty-free basis to BMJ Publishing Group Ltd ("BMJ") its licensees and where the relevant Journal is co-owned by BMJ to the co-owners of the Journal, to publish the Work in this journal and any other BMJ products and to exploit all rights, as set out in our licence.

The Submitting Author accepts and understands that any supply made under these terms is made by BMJ to the Submitting Author unless you are acting as an employee on behalf of your employer or a postgraduate student of an affiliated institution which is paying any applicable article publishing charge ("APC") for Open Access articles. Where the Submitting Author wishes to make the Work available on an Open Access basis (and intends to pay the relevant APC), the terms of reuse of such Open Access shall be governed by a Creative Commons licence – details of these licences and which Creative Commons licence will apply to this Work are set out in our licence referred to above.

Other than as permitted in any relevant BMJ Author's Self Archiving Policies, I confirm this Work has not been accepted for publication elsewhere, is not being considered for publication elsewhere and does not duplicate material already published. I confirm all authors consent to publication of this Work and authorise the granting of this licence.

For peer review only

Title: Care seeking for under-five children during the first two waves of the COVID-19 pandemic in Lagos State, Nigeria: a qualitative exploratory study

Authors: Ayobami A Bakare[1,2]*, Omotayo E Olojede[3], Carina King[2], Hamish R Graham[4], Obioma Uchendu[1,5], Tim Colbourn[6], Adegoke G Falade[3,7], Helle Mölsted Alvesson[2] on behalf of the INSPIRING Project Consortium**

Affiliations

[1] Department of Community Medicine, University College Hospital, Ibadan, Nigeria.
[2] Department of Global Public Health, Karolinska Institutet, Stockholm, Sweden.
[3] Department of Paediatrics, University College Hospital, Ibadan, Nigeria.
[4] Centre for International Child Health, Murdoch Children's Research Institute, University of Melbourne, Royal Children's Hospital, Parkville, Victoria, Australia.
[5] Department of Community Medicine, College of Medicine, University of Ibadan, Ibadan, Nigeria.
[6] Institute for Global Health, University College London, London, UK.
[7] Department of Paediatrics, College of Medicine, University of Ibadan, Ibadan, Nigeria.

***Corresponding author**

bakare.ayobami.adebayo@ki.se

Department of Global Public Health, Karolinska Institutet, Stockholm.

****Full INSPIRING consortium:** Carina King (Karolinska Institutet); Tim Colbourn, Rochelle Ann Burgess, Agnese Iuliano (UCL); Hamish Graham (Melbourne); Eric McCollum (Johns Hopkins); Tahlil Ahmed, Samy Ahmar, Christine Cassar, Paula Valentine (Save the Children UK); Adamu Isah, Adams Osebi, Magama Abdullahi, Ibrahim Haruna (Save the Children Nigeria); Temitayo Folorunso Olowookere (GSK Nigeria); Matt MacCalla (GSK UK); Adegoke Gbadegesin Falade, Ayobami Adebayo Bakare, Obioma Uchendu, Julius Salako, Funmilayo Shittu and Damola Bakare, Omotayo Olojede (University of Ibadan).

Keywords: COVID-19, lockdown, under-five children, care-seeking, Nigeria

Word count: 4703 words (excluding declarations, references and tables)

For peer review only - http://bmjopen.bmj.com/site/about/guidelines.xhtml

ABSTRACT (270/300)

Objective

To explore health care-seeking practices for children during the first two waves of COVID-19 in Lagos State, Nigeria and to understand context-specific direct and indirect effects of public health interventions for COVID-19.

Setting and participants

A qualitative explorative study involving 19 semi-structured interviews with healthcare providers from public and private primary health facilities and 32 interviews with caregivers of under-five children in Lagos, from December 2020 to March 2021. Participants were purposively selected from healthcare facilities to include community health workers, nurses, and doctors, and interviews were conducted in quiet locations at facilities. A data-driven reflexive thematic analysis according to Braun & Clark 2019 was conducted.

Results

Two themes were developed on appropriating COVID-19 in the belief systems, and ambiguity about COVID-19 preventive measures. The interpretation of the COVID-19 disease ranged from fearful to considering it as a 'scam' or 'falsification from the government'. Underlying distrust in government fueled COVID-19 misperception. Care seeking for children under-five was affected, as facilities were seen as contagious places for COVID-19. Caregivers resorted to alternative care and self-management of childhood illnesses. COVID-19 vaccine hesitancy was a major concern among healthcare providers compared to community members at the time of vaccine roll-out in Lagos Nigeria. Indirect impacts of COVID-19 lockdown included diminished household income,

For peer review only - http://bmjopen.bmj.com/site/about/guidelines.xhtml

worsening food insecurity, mental health challenges for caregivers and reduced clinic visits for immunization.

Conclusion: The first wave of the COVID-19 pandemic in Lagos was associated with reductions in care seeking for children, clinic attendance for childhood immunizations, and household income. Strengthening health and social support systems with context-specific interventions and containing misinformation is crucial to building adaptive capacity for response to future pandemics.

For peer review only

What is already known on this topic

- Data on the impact of COVID-19 prevention measures on childhood illnesses and hospital admissions are mostly from high-income countries, suggesting reduced health service utilization and acute morbidity during the early periods of the pandemic.
- However, few studies have explored the changes in care-seeking behaviour for children during this period, especially from low-and-middle-income countries which share a higher burden of childhood morbidity and mortality.

What this study adds

- Underlying distrust in government fuelled misinformation about the virus and shaped the perception of public health measures including vaccine acceptability.
- COVID-19 restrictions affected care-seeking for children in Lagos, Nigeria, through both direct and indirect mechanisms.

How this study might affect research, practice or policy.

- Future outbreak responses should include effective communication strategies to tackle rumours and misinformation, and when applicable, set up mobile clinics to prevent reduced healthcare access among children.
- COVID-19 global responses should be integrated into the existing health systems to avoid neglect of other important causes of morbidity and mortality.
- The global COVID-19 vaccine programme requires equity and transparency, and more research-led advocacy is needed to improve vaccine uptake among healthcare providers.

For peer review only - http://bmjopen.bmj.com/site/about/guidelines.xhtml

INTRODUCTION (word count=557)

The COVID-19 pandemic was declared a public health emergency of international concern in January 2020 by the World Health Organization.[1] Differential negative impacts have been reported across the globe due to the COVID-19 pandemic. While some countries have reported a high number of deaths due to COVID-19, others particularly in sub-Sahara Africa have reported low mortality,[2] but have suffered significant social and economic impacts with recovery likely to take a protracted course.[3] As of March 27, 2022, over eight million cases and 170,000 deaths have been reported in Africa, although estimates of actual cases (505.6 million) and deaths (439,500) in the region is much higher.[45] Within Africa, Nigeria reported the fourth highest number of COVID-19 cases in 2020-2021, with 215,164 reported cases (3.4% of the African total) and 92 million estimated actual cases.[6]Lagos State was the epicentre of the COVID-19 pandemic in Nigeria during this period, accounting for more than 30% of Nigeria's reported cases, with the first cases identified in late February 2020. [7,8]

The pandemic has been a major stressor to health systems, exposing and exacerbating pre-existing fragility and inequities within the system. [9,10] Given the absence of effective and widely available COVID-19 treatments during the first and second waves—February-October 2020 and November 2020-April 2021 respectively, [11] containment measures were based on public health measures like movement and travel restrictions (i.e. 'lockdowns'), physical distancing, personal hygiene and use of personal protective equipment (PPE). [12] Negative impacts of these containment measures on social life and mental well-being, education, economy, health service delivery and utilization have been reported, but mostly from non-empirical data and outside the African context.[13–18] Early predictions of Africa being worst hit by the COVID-19 pandemic did not come to fruition,[19] underscoring the need for context-specific empirical data. While the direct clinical impact of COVID-19 has affected adults more directly to date, children are not exempt from indirect effects of mitigations, although empirical data from Africa is lacking. [20,21]

In March 2020, the Nigerian government imposed several public health measures. The initial COVID-19 pandemic wave in Nigeria was characterized by fear, confusion and instability in the existing social structures, with misinformation fueled by social media reports and lockdown measures imposed by the government.[7,22–25] These may have had knock-on effects on healthcare service utilization and delivery. While multiple studies, largely from high-income contexts, have reported reductions in child illnesses and hospital admissions during periods of COVID-19 restrictions, fewer have explored the role of changes in care-seeking behaviour for children during this period and their implications for future public health responses to disease outbreaks.[16,26]

In Nigeria, under-five mortality remains high, and the yearly trajectory is not likely to meet the 2030 Sustainable Development Goal global target of having less than 25 deaths per 1000 live births.[27] Pneumonia, malaria and diarrhoea are leading causes of under-five deaths in the country, responsible for almost 40% of under-five deaths in 2018.[28] Nigeria also experiences multiple outbreaks of diseases of public health significance annually, including meningococcal disease, Yellow fever, and Lassa fever. [29] Given the existing burden of pneumonia, malaria, and diarrhoea among children, the magnitude of the COVID-19 pandemic and response, and the frequency of disease outbreaks requiring public health response, it is important to understand how the COVID-19 pandemic affected care-seeking for under-five children. We therefore aimed to understand care-seeking practices for young children during the first two waves of COVID-19 in Lagos State, to provide a context-specific understanding of the indirect and direct effects of COVID-related public health measures.

For peer review only - http://bmjopen.bmj.com/site/about/guidelines.xhtml

METHODS (word count=967)

Study design

This was an exploratory qualitative study, using semi-structured interviews with caregivers of children under-five and healthcare providers, gathering perspectives on care-seeking practices during the first two waves of the COVID-19 pandemic in Lagos State, Nigeria (February-October 2020 and November 2020-April 2021). The study was conducted as part of the process evaluation of the Lagos INSPIRING project, which is evaluating a child pneumonia health system intervention (study registration: ACTRN12621001071819). We followed the Consolidated Criteria for Reporting Qualitative Research (COREQ) guidelines for reporting.[30]

Study setting

The study was conducted in Ikorodu Local Government Area (LGA) in Lagos State. Lagos is the most populous state in Nigeria with an estimated population of 24.6 million people in 2022, [31] and is an economic hub in West Africa. Ikorodu is one of five administrative divisions of Lagos megacity. It is a peri-urban area, with fishing as the predominant economic activity in the rural parts of the LGA, and small and medium scale entrepreneurship as the major economic activity in the urban parts of the LGA. The LGA is served by two government-owned secondary health facilities (General Hospitals), 28 primary healthcare centers (PHCs) and over one hundred private facilities. Of the 28 PHCs, seven are designated as 'flagship' facilities by the Lagos State government, because they have more personnel and equipment and run 24-hour services for children and adults. There is at least one flagship PHC in each of Ikorodu's six Local Council Development Areas (LCDAs) and all of them remained open during the first two waves of the pandemic. The flagship PHCs are COVID-19 vaccination centres, except one facility without a medical doctor.

As part of the public health measures, Lagos was placed on lockdown by the Federal Government of Nigeria on the 30th March 2020.[7] The lockdown was a 35-day period characterized by a ban on social and economic activities, restriction of all non-essential movements, suspension of commuter services, closure of schools and retail shops and prohibition of mass gatherings except for funeral services.[32] Unlike PHCs and private health facilities, service provisions were limited to emergency cases in the public secondary-level facilities Thereafter, a gradual easing of the lockdown commenced from the 4th May 2020 with no re-instatement of movement restrictions during the second wave (see Appendix I).[7] In addition, there was a period of civil unrest in Lagos including Ikorodu LGA (the 'EndSARS' protests against police brutality [33]) between 8th and 22nd October 2020, when a curfew was imposed.

Study participants and sampling

We purposively selected healthcare providers who attended to sick children from the seven flagship PHCs and six nearby private facilities (Table 1). To ensure representation of each category of healthcare providers, the categories of staff (nurse, community health workers, and doctors) was adapted to each facility. We recruited caregivers of children under-five years presenting at the outpatient departments (i.e. with an illness) or immunization clinics (i.e. healthy children) of seven flagship PHCs and two secondary hospitals. Caregivers were recruited by female clinical data collectors (project staff), who screened every child brought to facility outpatients for pneumonia. In each facility, we conveniently recruited four caregivers of under-five children at random: two caregivers of an acutely unwell child (from out-patient clinic) and two caregivers of a child with no current illness episode (from the immunization clinic).

For peer review only - http://bmjopen.bmj.com/site/about/guidelines.xhtml

Table 1 Summary of participants' characteristics

**Caregivers n=32**		**Healthcare providers n=19**	
**Gender**		**Gender**	
Male	0 (0.0)	Male	5 (26.0)
Female	32 (100.0)	Female	14 (74.0)
Mean age (±SD)	31±5.0 years	Mean age (±SD)	38±8.1 years
**Median no of children (range)**	2 (1-5)	**Median year of experience**	11 (2-40)
**Educational level**		**Educational level**	
Primary	2 (6.3)	Diploma	9 (47.4)
Secondary	13 (40.6)	Tertiary	9 (47.4)
Tertiary	17 (53.1)	Postgraduate	1 (5.2)
**Religion**		**Religion**	
Christianity	25 (78.1)	Christianity	15 (78.9)
Islam	7 (21.9)	Islam	4 (21.1)
**Occupation/Cadre**		**Occupation/Cadre**	
Self-employed	21 (64.5)	Doctor	7 (36.8)
Employed	5 (16.1)	Nurse	6 (31.6)
No employment	6 (19.4)	CHEW	6 (31.6)

CHEW, Community Health Extension Worker;

Data collection

Interviews were conducted from 10 December 2020 to 18 March 2021. The semi-structured interview guides were based on the literature on care-seeking practices and knowledge about COVID-19 during the INSPIRING project formative phase and revised to capture the emerging COVID-19 vaccine programme roll-out in Nigeria. The interview guide for caregiver interviews had three sections, focused on: participants' family and socio-demographic information, their experiences of 2020 in light of COVID-19 including their perception of the illness and economic impacts, and care-seeking practices for children under-five years. The interview guide for healthcare provider interviews had three sections focusing on: service provision, facility adaptation to the COVID-19 pandemic, and care seeking for sick under-five children (Appendices II-IV). The research team was composed by pediatricians, social scientist and public health specialists.

The interviews were conducted by OEO, a Masters student with experience of the context and based in Nigeria. A female clinical study staff based at each facility was present in the interviews. Interviews were conducted in English or Yoruba (the indigenous local language in Ikorodu LGA), depending on the participant preference. The interviewer lived in Ikorodu before and during the COVID-19 pandemic and had previously visited the participating health facilities for other data collection activities. [34] Caregivers' interviews were conducted at the health facility or in another convenient place agreed by the participants. Providers' interviews were held at the facility. Each interview lasted between 30-40 minutes. All interviews were voice-recorded, transcribed and translated into English, before being stored in a secure cloud platform with access granted to only research team members.

For peer review only - http://bmjopen.bmj.com/site/about/guidelines.xhtml

Data analysis

After cross-check of transcripts, the analysis team (AAB, OEO, HMA and CK) conducted a data-driven thematic analysis to develop themes and subthemes.[35] AAB and OEO independently reviewed all the transcripts to identify initial codes which were reconciled in NVivo. [36] Healthcare provider and caregiver interviews were initially coded separately, reviewed by the analysis team to identify common themes and sub-themes, which were refined in subsequent analysis meetings. The process continued till the patterns of meaning were clear. The unit of analysis was COVID-19 related responses in the interviews.

Patient and Public Involvement

The overarching study was designed through a co-design workshop involving representatives from the Nigerian governments, community-based organizations, professionals, Save the Children and evaluation partners. However, patients were not involved in the design of this study. Findings from this study will be incorporated into the final report that will be disseminated to the relevant stakeholders including healthcare providers and community-based organizations. [37]

RESULTS (2114 excluding table 2)

We identified two overarching themes which were common to caregivers and healthcare workers: appropriating COVID-19 in the belief systems, and ambiguity towards preventive measures (Table 2).

For peer review only - http://bmjopen.bmj.com/site/about/guidelines.xhtml

Table 2: Summary of themes, categories and subcategories

**Theme**	**Categories**	**Sub-categories**	**Codes**
Appropriating COVID-19 in the belief systems	Political placement of COVID-19	Disbelief in the virus' existence	Covid is a deceit from the Nigerian government<br>It's a lie, a scam and a falsehood
		Misinformation and misconceptions about COVID-19	Denial of susceptibility<br>Seek care in the hospital and be added to the government COVID-19 list
	Socio-theological placement of COVID-19	Religious explanation for COVID-19	A test of faith from God requiring prayers<br>Strange event portraying end-times<br>God's judgement upon us<br>Devil's work
		Social placement of COVID-19	COVID-19 is not a poor disease<br>COVID-19 is not a black man's disease
	Medical placement of COVID-19	COVID-19 infection is real	COVID-19 is not good for one's health<br>COVID-19 is a serious infection<br>symptomatic diagnosis of COVID-19
		Healthcare as a source of infection	Self-management of infections<br>face masking in hospitals<br>More cautious in a hospital setting<br>Visit hospital and contract COVID-19<br>Healthcare workers are considered to be at high risk for COVID-19 infection<br>Delayed care seeking for illnesses
Ambiguity about COVID-19 preventive measures	Unappealing lockdown experiences and associated adaptive mechanisms	Direct impact of lockdown	Restriction to a home environment.<br>Lack of transportation preventing access to care
		Indirect impact of lockdown	Diminished household income<br>Mental health challenges during lockdown<br>Worsening food insecurity<br>Low immunization clinic attendance<br>Avoidance of social functions
		Health system adaption and its consequences	Physical distancing and hand washing at health facilities<br>Provision of shelter at health facilities<br>Compulsory use of face mask to gain entry to the hospital<br>Transport support for healthcare providers<br>Reduced manpower at work<br>Nonchalant attitude to the use of face mask<br>Care-seeking from an inappropriate person for lack of face mask<br>Denial of care

For peer review only

For peer review only - http://bmjopen.bmj.com/site/about/guidelines.xhtml

Theme	Categories	Sub-categories	Codes
	Drivers of COVID-19 vaccine hesitancy	Misinformation and conspiracy theories about COVID-19 vaccine	Vaccine is a mark of the beast<br>A depopulation strategy<br>The vaccine has been cloned
		Fear and worries about COVID-19 vaccines	Fear of vaccine side effects<br>Uncertainty about vaccine constituents
		Distrust in government efforts regarding COVID-19 vaccines	Uncertainty about the quality and effectiveness of vaccines sent to Nigeria
		Media influence on COVID-19	Vaccine rejection from other countries<br>Negative media reports promoting fear and hesitancy
	Drivers of COVID-19 vaccine uptake	Motivation to accept COVID-19 vaccine among healthcare providers	Perceived higher risk of infection,<br>Possibility of vaccine scarcity<br>Sense of responsibility to patients<br>Motivation from senior colleagues or health managers<br>Positive testimonies from recipients (including via social media platforms)<br>Trust in government efforts<br>For protection against COVID-19 infection
		Motivation to accept COVID-19 among community members or caregivers	Requirement for overseas travel or pilgrimage,<br>Health education and counseling<br>Public awareness from the government<br>Good attitude from healthcare providers<br>Trust in the existing routine immunization programme<br>Utilization of existing routine immunization programme<br>No observed adverse effects in early recipients<br>Being affordable

For peer review only

For peer review only - http://bmjopen.bmj.com/site/about/guidelines.xhtml

Appropriating COVID-19 in the belief systems

This first theme elucidates plurality in the placement of COVID-19 within the context of existing belief systems.

Caregivers and health care providers ascribed various causes to the emergence of COVID-19 including political, religious, social and geographical dimensions. The COVID-19 pandemic was framed through a political lens, with distrust in the government shaping their disbelief in the disease. This distrust in government provided an opening for misinformation about the virus and control measures with participants describing COVID-19 as "a lie", "a deceit from the government". This distrust also fed into perceptions about COVID-19 surveillance, with some caregivers reportedly delaying care seeking to avoid being automatically added to the COVID-19 daily government case list. The disbelief of the existence of COVID-19 had social associations with participants believing that the disease would not affect the poor or black man.

"There were some people that were like nothing is happening, we've not seen someone with it here, none of our relatives had it so it's just a scam. They don't believe it, most people don't believe it". (CHEW—female, public facility)

To others, COVID-19 was symbolic and they offered religious explanations, describing it as a test of faith, signs of the 'end of time', a "punishment from God" or the "work of the devil".

"It's just like God wanted to deliberately punish people for their bad behaviours. Because the leaders of Nigeria can't understand. Everything is in God's hands now. It's just like it's our sin that God is [punishing us on]. That's what's on ground now. Because they themselves don't know the next step they'll take again. Before, when one is sick, they'll say they should carry the individual, if it's our governors, they'll take flight and fly them out of the country. But when COVID-19 came, no one can come inside or go outside. Everyone is static (immobile in lockdown), so it's not COVID-19 again. It's God's judgement on us." (Mother—sick child, 1 child)

Others believed that COVID-19 existed as a symptomatic disease caused by a medical germ. Healthcare facilities were described as a source of infection "contagious" and hospital avoidance during the acute phase of the pandemic was reported by both caregivers and healthcare providers.

Given health facilities were considered high-risk places, this perception resulted in (i) no care-seeking practices for some sick under-five children as caregivers resorted to self-treatment of their child's illness by seeking care from drug sellers instead, and (ii) delayed presentation at health facilities when the child's condition had worsened. Similarly, when caregivers identified COVID-19 signs in their child they avoided hospital for fear of COVID-19 diagnosis or referral to isolation.

They didn't come. A lot of people were practicing self-medication. People who had cough for example, they didn't come for treatment for fear of being told they had COVID. They kept managing it at home. (CHEW—female, public facility)

"Like one of my neighbours when her baby was running a temperature, she as in, she could not bring the baby to the hospital because she said when she goes to the hospital - now they will say her baby have this thing high fever, they should take him to isolation center. Because of that she now went to the pharmacy and brought some (medicine) as in self medication" (Mother—healthy child, 3 children)

Both caregivers and healthcare workers reported being extra-careful in hospital settings, and sometimes this led to inaccessibility of care if healthcare providers suspected COVID-19 or had inadequate protective equipment. In contrast, one healthcare provider noted that service delivery for children did not change, stating that COVID-19 infections in children are not as severe as that of adults, and it would be unethical to deny children access to healthcare.

Ambiguity about COVID-19 preventive measures

This theme details various responses, experiences, and effects of recommended COVID-19 preventive measures and associated adaptations.

For peer review only - http://bmjopen.bmj.com/site/about/guidelines.xhtml

The lockdown was perceived as an unpleasant and difficult period as participants were restricted to indoor livelihood with little or no access to transportation.

Caregivers reported indirect effects of lockdown that could affect care-seeking. Caregivers reported diminished household incomes which necessitated loan acquisition or seeking help from family members. Household food insecurity was exacerbated, and caregivers started reducing their consumption to save food for their children. There was avoidance of social functions, mental health challenges and focus on basic needs:

"l have two teachers in my compound, not government teachers but private teachers. When the lockdown started then, the man is a teacher in private school, the woman is a teacher in a private school. As the school was not open, no salary, no money, nothing, nothing. For them to feed was problem, talk less of [never mind] if the baby falls sick, and now there is no money to take the baby to hospital. Sometimes, they will go and do herbal, this thing agbo (herbal concoction)" (Mother—healthy child, 3 children)

Health facilities made adjustments to ensure continuous service delivery without undermining safety. Face-masking, physical distancing, and improved personal hygiene were adopted amongst others; however, they created additional problems such as discomfort (face-masking), denied access to care, or seeking medical advice from people without medical training. Caregivers complied with the rule although there were reports of anger and verbal assaults with healthcare providers when they were enforced at the health facilities. There was a continuation of routine vaccination services during the lockdown, but caregivers' incorrect assumption of closure of PHC facilities during the lockdown (secondary facilities were close to non-emergency cases), compliance with the lockdown order and fear of coronavirus partly contributed to reduced attendance at the immunization clinic as reported by a CHEW:

"If you remember even on social media (mass media), it was broadcasted that if what you want to do at the hospital is not very important, stay indoors and stay safe? So people adhered to that rule, to the extent that when we go for outreach services, we ask them why they haven't been coming for immunization. Then they will say it's because of the lockdown, and then corona stopped us from coming out. They would also claim they don't know that the facility still runs its services" (CHEW—female, public facility)

When COVID-19 vaccines became available in Nigeria, there were mixed perceptions and ambiguity towards them. Among some caregivers, the vaccine was regarded as "a mark of the beast", or a depopulation strategy from Western countries. Religious belief, misinformation and fear of side effects were reasons identified by caregivers for COVID-19 vaccine hesitancy. Healthcare providers, in contrast, expressed distrust in the government and were concerned about the vaccine safety, quality, short timeline for vaccine development and the government's aggression towards COVID-19. They believed the vaccines were not tested very well in Nigeria before being approved.

"The health system is at risk. Nobody has ever said this is the vaccine for TB, but look at COVID-19, everybody is rushing to bring it to us; on what basis? To protect ourselves or to do what? When we have not yet encountered the illness." (Nurse—female, private hospital)

"*That thing (COVID-19 vaccine) is not well tested that's my point. It's supposed to go through a series of tests before allowing it to come into this country. So I can not even advise anyone to take it*." (Nurse—female, private hospital)

Social media (WhatsApp, Facebook, Instagram) was identified as a source of misinformation about the vaccine. One healthcare provider queried the decision of the government to accept donated vaccines that are being rejected by other countries, as reported on social media. Similarly, vaccines sent to Nigeria were presumed to be of sub-optimal quality compared to the ones used abroad but this was linked to distrust in governments.

"Some people (healthcare providers) don't want to take it because of the thing we have seen on social media that if you take it, it can cause this and that" (CHEW—female, public facility)

However, some healthcare providers and caregivers had positive perceptions of the vaccine, describing it as beneficial to the recipients, such as preventing sudden death and protecting against the virus. Others also showed trust in the government believing that the government cannot bring vaccines if they are harmful. Some caregivers also expressed willingness to receive the vaccine given that they are utilizing an existing routine immunization programme.

"If the vaccine comes, we know there's a reason why the government brought it. It has a work it wants to accomplish, which is why they want to bring it; we will take it" (Mother—sick child, 4 children)

For peer review only - http://bmjopen.bmj.com/site/about/guidelines.xhtml

Perceived higher risk of infection, the possibility of vaccines becoming scarce, a sense of responsibility to clients, motivation from senior colleagues or health managers, and later on positive testimonies from recipients were identified as drivers of uptake among healthcare providers. Being a requirement for overseas travel or pilgrimage, counselling, and public awareness were reported by healthcare providers as drivers of vaccine uptake among community members. Few healthcare providers who had taken the vaccine identified self-reflection and personal inquiry as ways they dealt with the misinformation about the vaccine.

"I heard they were cloning the vaccine in some European countries. That was my fear but when I did my own research. I found out that there is no issue." (Doctor—female, public facility)

Despite the fear and negative perceptions, community members turned out en masse to receive the vaccine, and turnout exceeded expectations, making the supply inadequate.

*We were even surprised. I wasn't expecting people to come out. It was supposed to be a 10 day program, that's for two weeks (the initial plan for the first phase of the COVID-19 vaccine roll-out) 10 working days but we extended further for four weeks or thereabout. People were still coming, we had to tell them that there was no more vaccination. (*Doctor—male, public facility).

DISCUSSION (word count=1427)

It is important to understand both community and healthcare workers' perceptions and experiences during the initial COVID-19 waves to adapt the provision of health care services to children during future pandemics. In the Nigerian context, participants reported both direct and indirect effects on care seeking for children, especially during the acute lockdown periods. Both groups of participants interpreted the COVID-19 pandemic through medical, political, social and economic lenses; however religious interpretation of the pandemic was more prominent among caregivers. Care seeking for children under-five was affected in part due to the perception of healthcare as being contagious, fear of COVID-19 diagnosis, and limited access to transportation. Adapting to seek care from alternative sources for sick children was reported by both groups. COVID-19 vaccine hesitancy was a major issue among healthcare providers, but less so among community members at the time of vaccine roll-out in Lagos. The motivations for vaccine uptake differed for both groups, and social media seemed to play a crucial role in shaping the acceptability of COVID-19 vaccine.

Our study suggests that COVID-19 related misinformation, rooted in a general distrust of government and cutting across every aspect of COVID-19 response including vaccine roll-out, had negative influences on care-seeking for children. This resonates with findings elsewhere in Africa and globally that misinformation and misleading interpretation of health information (daily reporting of cases and deaths from COVID-19 and fear of being counted as a COVID-19 case, assumption of facility closure during the lockdown) contributed to hospital avoidance, [16,38,39] and therefore requires consideration and active management in future outbreaks. [40] Conversely, the diversity in COVID-19 placement could conceivably have positive influences on care seeking. For instance, religious beliefs relating to COVID-19 may provide emotional resilience and motivate caregivers to do everything possible to protect their children.[41] Fear of COVID-19 may similarly motivate caregivers to seek care early and get vaccinated and even disbelief in COVID-19 may motivate caregivers to go about business as usual.

While there were people who did not believe in COVID-19 and/or did not seek care to avoid being caught up in the response (e.g. wanting to avoid isolation centres), some took it seriously and many integrated religious interpretations into their understanding of the disease. A study conducted in Nigeria found that religion and religious institutions, focused on Christianity, could have a negative influence on illness perception and behaviour, but that most Nigerian Christians comfortably integrated religious and physical health domains.[42]Additionally, some religious organizations actively encouraged adherence to COVID-19 preventive measures.[42] These findings highlight the dynamic process of classifying new diseases, as seen in the emergence of Ebola disease,[43] and the need for socio-cultural considerations and community participation in public health planning and communication, as well as active feedback and management of rumours and misinformation during the response. [44,45]

For peer review only - http://bmjopen.bmj.com/site/about/guidelines.xhtml

When caregivers decided to seek care for their children, lack of transportation due to lockdown inhibited access. Our finding agrees with an online survey conducted in Nigeria,[46] but contrasts with a study conducted in the Netherlands which reported parental non-deterrence in care seeking for a sick child.[47] Though the nature of illness could have been responsible for this contrasting finding, given the different epidemiological profiles, differences in health systems, COVID-19 related public health measures, as well as better health literacy around COVID-19, also have modulating effects. As reported in the UK, positive experiences from the National Health Service and support from others were positive influencers of care seeking, whereas fear driven by media and community were barriers to parental care seeking.[48] Worsened household income and food security reported during the acute phase of COVID-19 are in keeping with findings in other African countries, and these have the potential to exacerbate child malnutrition and mortality.[49,50] Like in other settings,[51–54] we found evidence suggesting decreased childhood immunization during the lockdown but the extent is unclear because healthcare providers reported using outreach services to vaccinate defaulters.

Healthcare services being considered as high-risk settings for infection influenced care seeking practices for children. Similar to reports in Nigeria and elsewhere, caregivers were avoiding hospitals for fear of contracting COVID-19.[48,55–57] The resultant self-management of childhood illness and decreased healthcare service utilization are in keeping with other studies from Europe and Africa.[56–59] Studies within and outside Nigeria have also reported increased self-medication practice for the prevention and treatment of COVID-19 related symptoms but did not focus on self-medication for children during the pandemic.[60–62] A study conducted in Uganda also found higher neonatal mortality and morbidity during the lockdown.[63] Estimating the impacts of reduced hospital visits, seeking care from alternative sources, delayed hospital visits and increased self-medication for sick children was outside the scope of this study but will be crucial for understanding the indirect effects of COVID-19 public health measures. Nevertheless, our study supports the need for intelligent health communication and flexible approaches to increasing service delivery capacity, such as mobile clinic outreaches to maintain health care access for children.[20,64] A study conducted in the UK hypothesized that decreased incidence of childhood illness during the lockdown period contributed to low paediatric admission for common and severe childhood illness during the lockdown;[65] however, hospital avoidance, care seeking from alternative sources and delayed presentation should not be dismissed.

The underlying distrust in government influenced COVID-19 perceptions, and provided the platform for the growing misinformation about the pandemic and this in turn shaped vaccine hesitancy.[66,67] Our findings are in agreement with studies in Nigeria which found that non-adherence to recommended preventive measures for COVID-19 was centered on political distrust, stemming from decades of perceived bad governance.[67,68] The mixed perception towards COVID-19 in Nigeria was therefore not surprising and similar controversies have been reported across several regions globally.[69] In times of uncertainty, a coping strategy is to use religion to provide explanations for strange events,[70] and these may conflict with emerging scientific evidence (particularly as conclusions change with new data) and frustrate containment measures.[71] Our findings support the need for inclusive risk communication for epidemic preparedness and control. Moreover, intervention adaptation to suit local contexts is essential during emergency response to epidemics.[44] Early reported cases of COVID-19 in the country were among foreigners and high-profile politicians. Linking COVID-19 results to known public officers could have been responsible for the perception that COVID-19 is a disease of the elite. In addition, limited testing capacity could have driven the perception that COVID-19 is not real, as up to 80% of infected individuals are mild or asymptomatic.[72]

Interestingly, the demand for COVID-19 vaccine was reportedly higher than anticipated among community members despite negative media reports and conspiracy theories. This finding is consistent with a study conducted by Julio et al. which found higher willingness to receive COVID-19 vaccine in low-and-middle-income countries compared to high income countries in which the survey was done.[73] Our findings support the call for vaccine equity, the need for sustained global partnership, and continuous post-vaccination surveillance to achieve effective global vaccination for COVID-19.[74] The concern about the unprecedented short period to vaccine production and licensing underscores the need for sustained and increased efforts toward control of other communicable diseases like tuberculosis, HIV/AIDS, and pneumonia—not neglecting other diseases because of COVID-19. Considering the background mistrust in government, donation of substandard vaccines, and vaccines with short expiry dates or not valid for travel as well as conditional donation of vaccines feeds into public narratives of lack of trust in COVID-19 vaccines and reinforces conspiracy theories about COVID-19.[75–77] Meanwhile, vaccine hesitancy among healthcare providers requires attention for increased and sustained COVID-19 vaccine coverage in the long term.[78]

For peer review only - http://bmjopen.bmj.com/site/about/guidelines.xhtml

This study has some limitations. We recruited caregivers from PHCs only and did not gather perspectives from other community members. This may mean that the perspectives captured here underestimates negative effects on care-seeking. Review of facility data shows a considerable decrease in out-patient attendance for children (Appendix V). Our findings have provided context-specific understanding of the indirect and direct effects of COVID-related public health measures and may inform future public health responses to disease outbreaks. Though the implementation of lockdown is context-specific, findings from our study may be transferrable to other low and middle-income countries with a similar weak health system and where distrust of government has been a problem.

CONCLUSIONS

The interpretation of the emergence of a new disease classification is dynamic and multi-faceted. The COVID-19 pandemic in Lagos had both direct and indirect effects on care-seeking for children. It is plausible that these had negative impacts on morbidity and mortality. Subsequent disease outbreak response requires active management of misinformation and intelligent health communication, including context-specific understanding of social-media messaging and the role of religious institutions. Strengthening health and social support system interventions, notably around ensuring access to healthcare is not negatively affected, is crucial to building adaptive capacity for future disease outbreaks, pandemics and building public trust.

For peer review only

For peer review only - http://bmjopen.bmj.com/site/about/guidelines.xhtml

Acknowledgments

We thank the clinical data collectors and facility heads for their support during the data collection, and the caregivers and healthcare workers for giving us their time.

Authors contributions

AAB, OEO, CK and HMA conceived of the study and TC, CK and AGF are grant holders. AAB designed the study. OEO collected the data with oversight from AAB and OCU. AAB and OEO led the analysis, with support from HMA, CK and HG. The manuscript was drafted by AAB with support from OEO, CK and HMA. All authors contributed to revisions and approved the final manuscript.

Competing Interests

SA, TA, CC and PV are employed by Save the Children UK who are part of the partnership funding the research. TFO, MM are employees of GSK, a multinational for-profit pharmaceutical company that produces pharmaceutical products for childhood pneumonia, including a SARS-CoV-2 vaccine, and no direct financial interests in oxygen or pulse oximeter products.

Funding

This work was funded through the GlaxoSmithKline (GSK)-Save the Children Partnership (grant reference: 82603743). Employees of both GSK and Save the Children UK contributed to the design and oversight of the wider INSPIRING study as part of a co-design process but did not take part directly in this sub-study. Any views or opinions presented are solely those of the author/publisher and do not necessarily represent those of Save the Children UK or GSK, unless otherwise specifically stated.

Data Availability Statement

Data are available upon reasonable request. Transcripts of interviews conducted are available in English may be shared based on nature of request to bakare.ayobami.adebayo@ki.se

Ethics Approval

We obtained ethical approvals from the following ethics committees: Lagos State Primary Health Care Board (ref: LS/PHCB/MS/1128/VOL.V1/005), University of Ibadan/University College Hospital (Ref: UI/EC/19/0551) and the University College London (Ref: 3433/005). We obtained informed oral consent from all the participants and conducted the interviews under strict adherence to the study COVID-19 prevention protocol.

For peer review only

References

1. World Health Organization (WHO). Statement on the second meeting of the International Health Regulations (2005) Emergency Committee regarding the outbreak of novel coronavirus (2019-nCoV). *Geneva, Switzerland*. 2005;(2005):1-6.

2. Adams J, MacKenzie MJ, Amegah AK, et al. The conundrum of low covid-19 mortality burden in sub-saharan africa: Myth or reality? *Glob Health Sci Pract*. 2021;9(3):433-443. doi:10.9745/GHSP-D-21-00172

3. Sub-Saharan Africa Exits Recession in 2021 but Recovery Still Vulnerable. Accessed December 8, 2021. https://www.worldbank.org/en/news/press-release/2021/10/06/sub-saharan-africa-exits-recession-in-2021-but-recovery-still-vulnerable

4. World Health Organization. *COVID-19 Weekly Epidemiological Update*. 85th ed.; 2022. Accessed April 2, 2022. https://www.who.int/publications/m/item/weekly-epidemiological-update-on-covid-19---29-march-2022

5. Waogodo Cabore J, Cyprian Karamagi H, Kipchumba Kipruto H, et al. Articles COVID-19 in the 47 countries of the WHO African region: a modelling analysis of past trends and future patterns. *Lancet Glob Health*. Published online 2022. doi:10.1016/S2214-109X(22)00233-9

6. COVID19_Cases. Accessed December 9, 2021. https://who.maps.arcgis.com/apps/dashboards/0c9b3a8b68d0437a8cf28581e9c063a9

7. Lanre R, Bello K, Olatunde O. Easing of lockdown measures in Nigeria: Implications for the healthcare system. *Health Policy Technol*. 2020;9(January):399-404. https://www.ncbi.nlm.nih.gov/pmc/articles/PMC7490626/

8. Amzat J, Aminu K, Kolo VI, Akinyele AA, Ogundairo JA, Danjibo MC. Coronavirus outbreak in Nigeria: Burden and socio-medical response during the first 100 days. *International Journal of Infectious Diseases*. 2020;98(January):218-224. doi:10.1016/j.ijid.2020.06.067

9. Esmé B, Nick G, Max L, Pablo ARM, Anjela T, and Diego AVP. *The Inequality Virus: Bringing Together a World Torn Apart by coronavirus through a Fair, Just and sustainable Economy*. Vol 1. Oxfam GB; 2021.

10. Jensen N, Kelly AH, Avendano M. The COVID-19 pandemic underscores the need for an equity-focused global health agenda. *Humanit Soc Sci Commun*. 2021;8(1). doi:10.1057/s41599-020-00700-x

11. Akande OW, Elimian KO, Igumbor E, et al. Epidemiological comparison of the first and second waves of the COVID-19 pandemic in Nigeria, February 2020–April 2021. *BMJ Glob Health*. 2021;6(11):e007076. doi:10.1136/BMJGH-2021-007076

12. Odusanya OO, Odugbemi BA, Odugbemi TO, Ajisegiri WS. COVID-19: A Review of the Effectiveness of Non-Pharmacological Interventions. *NIgerian Postgraduate Medical Journal*. 2020;27(4):1-7. doi:10.4103/npmj.npmj

13. Verschuur J, Koks EE, Hall JW. Global economic impacts of COVID-19 lockdown measures stand out in highfrequency shipping data. *PLoS One*. 2021;16(4 April):1-16. doi:10.1371/journal.pone.0248818

14. Ozili PK. COVID-19 Pandemic and Economic Crisis: The Nigerian Experience and Structural Causes. *SSRN Electronic Journal*. Published online 2020:1-19. doi:10.2139/ssrn.3567419

15. Chakraborty I, Maity P. COVID-19 outbreak: Migration, effects on society, global environment and prevention. *Science of the Total Environment*. 2020;728:138882. doi:10.1016/j.scitotenv.2020.138882

For peer review only - http://bmjopen.bmj.com/site/about/guidelines.xhtml

16. Conlon C, McDonnell T, Barrett M, et al. The impact of the COVID-19 pandemic on child health and well-being : Are children " slipping through the net " ? A qualitative study of frontline emergency care staff. *BMC Health Serv Res*. 2021;9(1):1-29. https://bmchealthservres.biomedcentral.com/articles/10.1186/s12913-021-06284-9

17. Impact of COVID-19 on people's livelihoods, their health and our food systems. Accessed March 15, 2022. https://www.who.int/news/item/13-10-2020-impact-of-covid-19-on-people%27s-livelihoods-their-health-and-our-food-systems

18. Passavanti M, Argentieri A, Barbieri DM, et al. The psychological impact of COVID-19 and restrictive measures in the world. *J Affect Disord*. 2021;283(September 2020):36-51. doi:10.1016/j.jad.2021.01.020

19. Berhan Y. Will Africa be Devastated by Covid-19 as Many Predicted? Perspective and Prospective. *Ethiop J Health Sci*. 2020;30(3):459-467. doi:10.4314/ejhs.v30i3.17

20. United Nations. *Policy Brief: The Impacts of COVID-19 on Children*. Vol 109.; 2020. doi:10.1111/apa.15484

21. Ashikkali L, Carroll W, Johnson C. The indirect impact of COVID-19 on child health. *Paediatr Child Health*. 2020;30(12):430-437. doi:10.1016/j.paed.2020.09.004

22. Abayomi O. Olaseni, Akinsola OS, Agberotimi SF, Rotimi Oguntayo. Psychological distress experiences of Nigerians during Covid-19 pandemic; the gender difference. *www.archbronconeumol.org Original*. 2020;(January).

23. Ogoina D. Covid-19 and the rest of us. *Niger Delta Medical Journal*. 2020;4(1):6-8.

24. Obi-Ani NA, Anikwenze C, Isiani MC. Social media and the Covid-19 pandemic: Observations from Nigeria. *Cogent Arts Humanit*. 2020;7(1). doi:10.1080/23311983.2020.1799483

25. Johnson OA, Olaniyi SF, John S, et al. "Infodemic" in a pandemic: COVID-19 Conspiracy Theories in an African Country. *Social Health and Behavior*. Published online 2020:19-24. doi:10.4103/SHB.SHB

26. Scaramuzza A, Tagliaferri F, Bonetti L, et al. Changing admission patterns in paediatric emergency departments during the COVID-19 pandemic. *Arch Dis Child*. 2020;105(7):704-706. doi:10.1136/archdischild-2020-319397

27. United Nations. *Transforming Our World: The 2030 Agenda for Sustainble Development*.; 2016.

28. WHO and Maternal and Child Epidemiology Estimation Group (MCEE). Global and Regional Child Deaths by Cause. 2018.

29. Testimony J Olumade, Oluwafolajimi A Adesanya, Iyanuoluwa J Fred-Akintunwa, et al. Infectious disease outbreak preparedness and response in Nigeria_ history, limitations and recommendations for global health policy and practice _ Enhanced Reader. *AIMS Public Health*. 2020;7(4):736-757.

30. Tong A, Sainsbury P, Craig J. Consolidated criteria for reporting qualitative research (COREQ): a 32-item checklist for interviews and focus groups. 2007;19(6):349-357.

31. About Lagos – Lagos State Government. Accessed February 24, 2022. https://lagosstate.gov.ng/about-lagos/

32. Presidential task force on COVID-19. *Implementation Guidance for Lockdown Policy*. Vol 5.; 2020. http://www.akrabjuara.com/index.php/akrabjuara/article/view/919

33. Lawal RA, Ibrahim K. *#ENDSARS: Effecting Positive Change in Governance in Nigeria BEYOND M a y 2 0 2 1*. www.bbforpeace.org

34. Graham HR, Olojede OE, Bakare AA, et al. Measuring oxygen access: Lessons from health facility assessments in Lagos, Nigeria. *BMJ Glob Health*. 2021;6(8). doi:10.1136/bmjgh-2021-006069

For peer review only - http://bmjopen.bmj.com/site/about/guidelines.xhtml

35. Braun V, Clarke V. Qualitative Research in Psychology Using thematic analysis in psychology Using thematic analysis in psychology. *Qual Res Psychol*. 2006;3(2):77-101. http://www.tandfonline.com/action/journalInformation?journalCode=uqrp20%5Cnhttp://www.tandfonline.com/action/journalInformation?journalCode=uqrp20

36. QSR International Pty Ltd. (2020) NVivo (released in March 2020).

37. Graham HR, Olojede OE, Bakare AAA, et al. Pulse oximetry and oxygen services for the care of children with pneumonia attending frontline health facilities in Lagos, Nigeria (INSPIRING-Lagos): Study protocol for a mixed-methods evaluation. *BMJ Open*. 2022;12(5). doi:10.1136/bmjopen-2021-058901

38. Nigeria records chloroquine poisoning after Trump endorses it for coronavirus treatment - CNN. Accessed January 5, 2022. https://edition.cnn.com/2020/03/23/africa/chloroquine-trump-nigeria-intl/index.html

39. What are the myths about the coronavirus in Africa? | World Economic Forum. Accessed January 5, 2022. https://www.weforum.org/agenda/2020/04/debunking-9-popular-myths-doing-the-rounds-in-africa-about-the-coronavirus/

40. Winters M, Oppenheim B, Sengeh P, et al. Debunking highly prevalent health misinformation using audio dramas delivered by WhatsApp: Evidence from a randomised controlled trial in Sierra Leone. *BMJ Glob Health*. 2021;6(11). doi:10.1136/bmjgh-2021-006954

41. Roberto A, Sellon A, Cherry ST, Hunter-Jones J, Winslow H. Impact of spirituality on resilience and coping during the COVID-19 crisis: A mixed-method approach investigating the impact on women. *https://doi.org/101080/0739933220201832097*. 2020;41(11-12):1313-1334. doi:10.1080/07399332.2020.1832097

42. Ossai EC. 'It is the antichrist. Can't you see?' Perceptions of COVID-19 among Nigeria's Christians and the Religion—Health Debate. *https://doi.org/103366/swc20210325*. 2021;27(1):48-64. doi:10.3366/SWC.2021.0325

43. Barry S Hewlett, Richard P. Amola. Cultural Contexts of Ebola in Northern Uganda. *Emerg Infect Dis*. 2003;9(10).

44. Owoyemi A, Okolie EA, Omitiran K, et al. Importance of community-level interventions during the COVID-19 pandemic: Lessons from sub-saharan Africa. *American Journal of Tropical Medicine and Hygiene*. 2021;105(4):879-883. doi:10.4269/ajtmh.20-1533

45. Dash S, Parray AA, de Freitas L, et al. Combating the COVID-19 infodemic: A three-level approach for low and middle-income countries. *BMJ Glob Health*. 2021;6(1). doi:10.1136/bmjgh-2020-004671

46. Briggs D, Kattey K. COVID-19 : Parents ' Healthcare-Seeking Behaviour for their Sick Children in COVID-19 : Parents ' Healthcare-Seeking Behaviour for their Sick Children in Nigeria - An Online Survey. 2020;(September). doi:10.9734/IJTDH/2020/v41i1330344

47. Tan CD, Lutgert EK, Neill S, et al. Parents ' experiences with a sick or injured child during the COVID- lockdown : an online survey in the Netherlands. Published online 2021:1-7. doi:10.1136/bmjopen-2021-055811

48. Watson G, Pickard L, Williams B, Hargreaves D, Blair M. Do I, don't I?' A qualitative study addressing parental perceptions about seeking healthcare during the COVID-19 pandemic. *Arch Dis Child*. 2021;106(11):1118-1124. doi:10.1136/archdischild-2020-321260

49. Kansiime MK, Tambo JA, Mugambi I, Bundi M, Kara A, Owuor C. COVID-19 implications on household income and food security in Kenya and Uganda: Findings from a rapid assessment. *World Dev*. 2021;137. doi:10.1016/j.worlddev.2020.105199

For peer review only - http://bmjopen.bmj.com/site/about/guidelines.xhtml

50. Osendarp S, Akuoku JK, Black RE, et al. The COVID-19 crisis will exacerbate maternal and child undernutrition and child mortality in low- and middle-income countries. *Nat Food*. 2021;2(7):476-484. doi:10.1038/s43016-021-00319-4

51. Emilia Connolly, Emma J Boley, Donald Luke Fejfar, et al. Childhood immunization during the COVID-19 pandemic: experiences in Haiti, Lesotho, Liberia and Malawi. *Bull World Health Organization*. Published online 2022.

52. Zhong Y, Clapham HE, Aishworiya R, et al. Childhood vaccinations: Hidden impact of COVID-19 on children in Singapore. *Vaccine*. 2021;39(5):780-785. doi:10.1016/J.VACCINE.2020.12.054

53. Chandir S, Siddiqi DA, Setayesh H, Khan AJ. Impact of COVID-19 lockdown on routine immunisation in Karachi, Pakistan. *Lancet Glob Health*. 2020;8(9):e1118-e1120. doi:10.1016/S2214-109X(20)30290-4/ATTACHMENT/620A1068-4BDB-41AC-9C3C-14F0A5E00589/MMC1.PDF

54. McDonald HI, Tessier E, White JM, et al. Early impact of the coronavirus disease (COVID-19) pandemic and physical distancing measures on routine childhood vaccinations in England, January to April 2020. *Eurosurveillance*. 2020;25(19):2000848. doi:10.2807/1560-7917.ES.2020.25.19.2000848/CITE/PLAINTEXT

55. Iwuoha VC, Aniche ET, Obiora CA, UMeifekwem UT. Citizens lack access to healthcare facilities_ How COVID-19 lockdown and social distancing policies boost roadside chemist businesses in South-Eastern Nigeria _ Enhanced Reader. *International Journal of Health Planning Management*. Published online 2021.

56. Davis AL, Sunderji A, Marneni SR, et al. Caregiver-reported delay in presentation to pediatric emergency departments for fear of contracting COVID-19: a multi-national cross-sectional study. *Canadian Journal of Emergency Medicine*. 2021;23(6):778-786. doi:10.1007/s43678-021-00174-z

57. Lazzerini M, Barbi E, Apicella A, Marchetti F, Cardinale F, Trobia G. Delayed access or provision of care in Italy resulting from fear of COVID-19. *Lancet Child Adolesc Health*. 2020;4(5):e10-e11. doi:10.1016/S2352-4642(20)30108-5

58. Ciacchini B, Tonioli F, Marciano C, et al. Reluctance to seek pediatric care during the COVID-19 pandemic and the risks of delayed diagnosis. *Ital J Pediatr*. 2020;46(1):1-4. doi:10.1186/s13052-020-00849-w

59. Singh DR, Sunuwar DR, Shah SK, et al. Impact of COVID-19 on health services utilization in Province-2 of Nepal: a qualitative study among community members and stakeholders. *BMC Health Serv Res*. 2021;21(1):1-14. doi:10.1186/s12913-021-06176-y

60. Wegbom AI, Edet CK, Raimi O, Fagbamigbe AF, Kiri VA. Self-Medication Practices and Associated Factors in the Prevention and/or Treatment of COVID-19 Virus: A Population-Based Survey in Nigeria. *Front Public Health*. 2021;9(June):1-9. doi:10.3389/fpubh.2021.606801

61. Onchonga D, Omwoyo J, Nyamamba D. Assessing the prevalence of self-medication among healthcare workers before and during the 2019 SARS-CoV-2 (COVID-19) pandemic in Kenya. *Saudi Pharmaceutical Journal*. 2020;28(10):1149-1154. doi:10.1016/j.jsps.2020.08.003

62. Osaigbovo L, Ogboghodo E, Obaseki D, et al. Pattern of Drug Sales At Community Pharmacies in Edo State As Evidence of Self-Medication During the Covid-19 Pandemic: Implications for Policy Implementtation. *J Chem Inf Model*. 2020;20(04):150-158.

63. Hedstrom A, Mubiri P, Nyonyintono J, et al. Impact of the early COVID-19 pandemic on outcomes in a rural Ugandan neonatal unit: A retrospective cohort study. *PLoS One*. 2021;16(12 December). doi:10.1371/JOURNAL.PONE.0260006

For peer review only - http://bmjopen.bmj.com/site/about/guidelines.xhtml

64. *Maintaining the Provision and Use of Services for Maternal, during the COVID-19 Pandemic: Lessons Learned from 19 Countries*.; 2021. licence: CC BY-NC-SA 3.0 IGO.

65. Kadambari S, Goldacre R, Morris E, Goldacre MJ, Pollard AJ. Indirect effects of the covid-19 pandemic on childhood infection in England: population based observational study. *BMJ*. Published online 2022. doi:10.1136/bmj-2021-067519

66. Agbo UM, Nche GC. Suspecting the Figures: What Church Leaders Think About Government's Commitment to Combating COVID-19 in Nigeria. *J Asian Afr Stud*. Published online 2022. doi:10.1177/00219096211069645

67. Wonodi C, Obi-Jeff C, Adewumi F, et al. Conspiracy theories and misinformation about COVID-19 in Nigeria: Implications for vaccine demand generation communications. *Vaccine*. 2022;40(13):2114-2121. doi:10.1016/J.VACCINE.2022.02.005

68. Ezeibe CC, Ilo C, Ezeibe EN, et al. Political distrust and the spread of COVID-19 in Nigeria. *Glob Public Health*. 2020;15(12):1753-1766. doi:10.1080/17441692.2020.1828987

69. Hardy LJ. *Connection, Contagion, and COVID-19*.

70. Adiyoso W, Kanegae H. The Preliminary Study of the Role of Islamic Teaching in the Disaster Risk Reduction (A Qualitative Case Study of Banda Aceh, Indonesia). *Procedia Environ Sci*. 2013;17:918-927. doi:10.1016/j.proenv.2013.02.110

71. Yau EKB, Ping NPT, Shoesmith WD, James S, Hadi NMN, Lin LJ. The behaviour changes in response to COVID-19 pandemic within Malaysia. *Malaysian Journal of Medical Sciences*. 2020;27(2):45-50. doi:10.21315/mjms2020.27.2.5

72. Wu Z, McGoogan J. Characteristics of and Important Lessons From the Coronavirus Disease 2019 (COVID-19) Outbreak in China: Summary of a Report of 72 314 Cases From the Chinese Center for Disease Control and Prevention. *JAMA*. Published online 2020.

73. Solís Arce JS, Warren SS, Meriggi NF, et al. COVID-19 vaccine acceptance and hesitancy in low- and middle-income countries. *Nat Med*. 2021;27(8):1385-1394. doi:10.1038/s41591-021-01454-y

74. Forman R, Shah S, Jeurissen P, Jit M, Mossialos E. COVID-19 vaccine challenges: What have we learned so far and what remains to be done? *Health Policy (New York)*. 2021;125(5):553-567. doi:10.1016/j.healthpol.2021.03.013

75. Statement on Covishield and the European Union (EU) Digital COVID Certificate "Green Pass" – Africa CDC. Accessed June 27, 2022. https://africacdc.org/download/statement-on-covishield-and-the-european-union-eu-digital-covid-certificate-green-pass/

76. Joint Statement on Dose Donations of COVID-19 Vaccines to African Countries. Accessed June 27, 2022. https://www.who.int/news/item/29-11-2021-joint-statement-on-dose-donations-of-covid-19-vaccines-to-african-countries

77. Lucien Hordijk, Priti Patnaik. Covid-19-How Europe's vaccine donations went tragically wrong. doi:10.1136/bmj.o1286

78. Ackah M, Ameyaw L, Gazali M, et al. COVID-19 vaccine acceptance among health care workers in Africa: A systematic review and meta-analysis. *PLoS One*. 2022;17(5):e0268711. doi:10.1371/JOURNAL.PONE.0268711

For peer review only - http://bmjopen.bmj.com/site/about/guidelines.xhtml

Appendix I: -Ease of COVID – 19 Lockdown in Nigeria

	Phase 1	Phase 2	Phase 3	Phase 4	Phase 5
**Start date**<br><br>**End date**	4th May 2020<br><br>1st June 2020	2nd June 2020<br><br>18th October 2020	19th October 2020	11th May 2021	2nd April 2022
**Land travel (Interstate)**	Banned except for essential services and movement of goods and services only	Opened under strict conditions:<br><br>Allowed for essential services and movement of goods and services only	Opened	Opened	Opened
**Land travel (Intrastate)**	Limited to 6 am-6 pm with a 50% reduction in bus occupancy	Opened	Opened	Opened	Opened
**Airspace**	Closed<br><br>Opened to cargo and specially approved flights only	Opened for domestic flights, limited for essential international flights until August 26	Opened (domestic and international flights)	Opened for domestic and international	Opened
**Movement**	Curfew from 8pm to 6am	Curfew from 10pm – 4am	12am to 4am	Curfew from 12am – 4am	No restrictions
**Working hours**	9 am to 2 pm	9 am – 2 pm for Government/other corporate offices	All government staff on grade level 12 and below to continue staying at home<br><br>No limit for private and other corporate bodies	All government staff on grade level 12 and below to continue staying at home until 11th June 2021<br><br>No limit for private and other corporate bodies	No limit
**Workspace**	50% staff occupancy or less	75% staff occupancy or less<br>50% for clients	100% occupancy	No limits but virtual meetings and work from home encouraged	No limit
**Entertainment activities**	Banned	Banned	Opened	Open with some restrictions (bars, night clubs, pubs remained closed)	Opened at 50% capacity
**Mass gathering**	Limited to 20 people or less	Limited to 20 people or less	Limited to 50 people or less	Limited to 50 people or less except	Opened

				with permission from the state government	
**Religious gathering**	Restricted	Restricted	Restricted (subject to the protocol from the state government and the federal capital territory	Limited to less than 50% capacity<br><br>Gathering more than 50 people must be held outdoors only	Opened
**Schools**	Closed	Closed, but special consideration for graduation exams	Opened	Opened	Opened
**Markets**	Partial closure, open only on designated days weekly between 8.00 am-3.00 pm	Controlled access by local authorities	Open	Open	Open
**Face masks**	Mandatory for all persons in public spaces	Mandatory for all persons in public spaces	Mandatory for all persons in public spaces	Mandatory for all persons in public spaces	Mandatory for indoor activities only, but at individual discretion for outdoor activities
**Banks and other financial institutions**	Limit staff physically to between 30%-50%	Limit staff physically to 75% or less. To operate normal working hours	To operate normal working hours	To operate normal working hours	To operate normal working hours

Source: NCDC Coronavirus COVID-19 Microsite. Accessed June 29, 2022. https://covid19.ncdc.gov.ng/guideline/

- *There was a total lockdown of economic activities in the FCT, Lagos and Ogun states for 35days from 30th March 2020. This was coupled with a total ban on non-essential interstate travels*
- *From the third phase, the end dates were assumed as the onset of the next phase*
- *Data collection was done during the phase 3*
- *The second wave of infection and vaccine rollout started during the phase 3*

For peer review only

Appendix II: In-depth interview guide for healthcare provider's interviews

1. Tell me about the facility you work in?
    a. What type of services do you offer children?
    b. Tell me specifically about this week in your clinic
2. Think about last 8 months, has things been typical? Why? Why not?
    a. When did you first hear about covid?
    b. When did you make adaptation or adjustment in your facility as a result of covid?
    c. What changes did your facility make?

NOW WE WANT TO FOCUS ON QUESTIONS REGARDING CHILDREN

3. Tell me how the lockdown in year 2020 affected service provision at your facility
    a. How did it affect services you provide for children?
    a. How did it affect care seeking for sick under-five?
        - Probe severity of illness at presentation/late presentation
        - Was the PHC the first point of call?

4. Thinking about this time last year, before covid/EndSARS, is there any differences? What is different (numbers, type of presentation, services provided, resources), what is the same?
5. Now that lockdown is over, have things normalized the way it used to be before COVID-19? What has normalized? What is yet to normalize. What about number of under-five that you see, any difference compared to last year in terms of number, type of presentation
6. Late in last year, there was Endsars protest. How did it affect service delivery in your facility?
7. Currently, there is second wave of Covid-19 in Nigeria. How has it affected service delivery in your facility?
8. What can you say about the care seeking behavior of caregivers of sick children you have attended to in recent times?
9. Between Covid-19 lockdown, End-Sars protest and current economic hardship, which one has affected care seeking for sick children most? Why? Short term consequences? Any long term consequences?
10. Finally, the federal government is making plans to procure Covid-19 vaccines for Nigerians. How willing are you to receive the vaccine? Why/why not? What about for your child/children? Why/why not? Will you tell others to take it? Why?
11. Do you have any other things to say?

For peer review only

Appendix III: In – Depth interview guide for caregiver of under-five with recent illness episode

1. Tell me about your family.
   Probe to get information on:
   a. Who lives with the participant
   b. Participant's job
   c. Where participant's extended family live
   d. Involvement in child's care
2. How will you summarize year 2020?
   Probe:
   a. How did it affect you and your family?
   b. Could you say these changes were due to impact of covid-19 pandemic?
      1. If yes, why? In what other ways have covid-19 affected you and your household
      2. If no, why not?
   c. Have you noticed changes in the price of commodities?
      i. How does this affect you and your household?
         1. House rent
         2. Transport cost
         3. Food items

Now we want to talk about child health services, particularly care seeking for sick under-five

3. Your child was recently sick; I would like to know more about the illness.
   a. How did it start? Who first noticed the symptoms?
   b. What did you do first? When did you do that?
   c. What next did you do?
      i. How did you decide?
      ii. Why did you do that? Could you have done something else?
      iii. What treatment were given? Was your child asked to do some tests? Could you afford all the test?
      iv. Were you referred?
         1. If yes, did you honour the referral? Why?
         2. How did you feel with the referral?
         3. If no, why? What did you do next? Why did you do that?
         4. Was your child asked to do some tests? Could you afford all the test?
         5. What about medications? Did you buy all the medication?
      v. Like how much did it cost you to treat your child? Would the cost have been cheaper if not for current situations? How did you cover the cost of treatments for your child?
         1. Personal money/savings?
         2. Support from father?
         3. Support family and friends?
         4. Did you have to borrow or sell any items?

4. Overall, has covid-19 affected your decisions and steps when your child was sick?

   i. If yes, how?
   ii. If no, what affected your decisions and steps?
      i. Endsars protest?

ii. Insecurity?
iii. Current economic hardship?
iv. Could you have taken different actions/steps (*relate this to previous answers*) What about fears of catching covid at the hospital?

5. Finally, the federal government has indicated that by Jan 2021 the country will have covid-19 vaccine. How willing are you to receive the vaccine? Why/why not? What about for your child/children? Why/why not?
6. Do you have other things to say or bring to my attention? Thank you for your time!

For peer review only

Appendix IV: IDI guide for No illness episode

1. Tell me about your family.
   Probe to get information on:
   e. Who lives with the participant.
   f. Participant's job
   g. Where participant's extended family live
   h. Involvement in child's care
2. How will you summarize year 2020?
   Probe:
   a. How has it affected you and your family?
   b. Were these covid-19 related?
      i. If yes, why and how? In what **other** ways have covid-19 affected you and your household
      ii. If no, what do you think is responsible?
   c. Have you noticed changes in the price of commodities?
      i. How does this affect you and your household?
         1. House rent
         2. Transport cost
         3. Food items
         4. Fuel price
3. Now we want to talk about care seeking for under-five. What actions do mothers/caregivers take when their child develops illness?
      i. Why do they do that?
         1. Could it be because of trust/distrust in health care workers?
         2. Could cost have influenced their decision? How?
         3. What else could have influenced their action?
      ii. Think about the last time your child (or that of someone close to you) fell sick
         1. What was wrong? What did you do? How did you decide on what to do? Who did you talk to? What alternatives were considered? What were your concerns?
         2. Was your child referred?
         3. Did you honour the referral?
         4. If yes, why? If no, why?
         5. If it happens this period, could you or they have taken different action? Why?
      iii. During the covid-pandemic in Nigeria, do you think covid-19 affected decisions taken by caregivers when their child was sick? if yes, why and how? If no, why?
      iv. What about now? Do covid-19 affect actions taken by mothers when their child falls sick?
      v. Between covid-19 and current economic hardship, which one has greater influence on actions taken by caregivers when their child is sick?
         1. Why and how?
4. Finally, the federal government has indicated that by Jan 2021 the country will have covid-19 vaccine. How willing are you to receive the vaccine? Why/why not? What about for your child/children? Why/why not?
5. Do you have any other thing to tell me?

Thank you for your time!

Appendix V Outpatient attendance for under-five children in the 7 flagship facilities in Ikorodu LGA (January-June 2020)*

Year	Flagship PHCs													
2020	**Ikorodu**		**Igbogbo**		**Odonla**		**Agbede**		**Ipakodo**		**Imota**		**Oke-Eletu¥**	
	**Diagnosis**	**Number**	**Diagnosis**	**Number**	**Diagnosis**	**Number**	**Diagnosis**	**Number**	**Diagnosis**	**Number**	**Diagnosis**	**Number**	**Diagnosis**	**Number**
**January—March**	Pneumonia	9	Pneumonia	3	Pneumonia	1	Pneumonia	0	Pneumonia	3	Pneumonia	19	Pneumonia	-
	LRTI	7	LRTI	12	LRTI	1	LRTI	0	LRTI	1	LRTI	2	LRTI	-
	URTI	133	URTI	290	URTI	89	URTI	0	URTI	102	URTI	47	URTI	-
	ARTI	0	ARTI	0	ARTI	0	ARTI	0	ARTI	5	ARTI	0	ARTI	-
	Malaria	511	Malaria	275	Malaria	149	Malaria	129	Malaria	234	Malaria	125	Malaria	-
	Sepsis	43	Sepsis	97	Sepsis	243	Sepsis	21	Sepsis	42	Sepsis	33	Sepsis	-
	Others	274	Others	374	Others	406	Others	115	Others	211	Others	252	Others	-
	**Total**	**977**	**Total**	**1051**	**Total**	**892**	**Total**	**265**	**Total**	**589**	**Total**	**478**	**Total**	**-**
**April—June**	Pneumonia	1	Pneumonia	0	Pneumonia	2	Pneumonia	0	Pneumonia	0	Pneumonia	1	Pneumonia	0
	LRTI	3	LRTI	1	LRTI	0	LRTI	0	LRTI	0	LRTI	0	LRTI	0
	URTI	10	URTI	55	URTI	5	URTI	1	URTI	25	URTI	2	URTI	34
	ARTI	0	ARTI	0	ARTI	0	ARTI	0	ARTI	0	ARTI	0	ARTI	0
	Malaria	215	Malaria	183	Malaria	64	Malaria	23	Malaria	39	Malaria	52	Malaria	42
	Sepsis	22	Sepsis	26	Sepsis	55	Sepsis	2	Sepsis	9	Sepsis	8	Sepsis	63
	Others	113	Others	39	Others	159	Others	5	Others	99	Others	125	Others	164
	**Total**	**364**	**Total**	**304**	**Total**	**285**	**Total**	**31**	**Total**	**172**	**Total**	**188**	**Total**	**303**

*Lagos placed on lockdown on the 30 March 2020

¥ Facility register not found

For peer review only

International Journal for Quality in Health Care; Volume 19, Number 6: pp. 349–357 10.1093/intqhc/mzm042
Advance Access Publication: 14 September 2007

Consolidated criteria for reporting qualitative research (COREQ): a 32-item checklist for interviews and focus groups

ALLISON TONG[1,2], PETER SAINSBURY[1,3] AND JONATHAN CRAIG[1,2]

[1]School of Public Health, University of Sydney, NSW 2006, Australia, [2]Centre for Kidney Research, The Children's Hospital at Westmead, NSW 2145, Australia, and [3]Population Health, Sydney South West Area Health Service, NSW 2170, Australia

Abstract

Background. Qualitative research explores complex phenomena encountered by clinicians, health care providers, policy makers and consumers. Although partial checklists are available, no consolidated reporting framework exists for any type of qualitative design.

Objective. To develop a checklist for explicit and comprehensive reporting of qualitative studies (indepth interviews and focus groups).

Methods. We performed a comprehensive search in Cochrane and Campbell Protocols, Medline, CINAHL, systematic reviews of qualitative studies, author or reviewer guidelines of major medical journals and reference lists of relevant publications for existing checklists used to assess qualitative studies. Seventy-six items from 22 checklists were compiled into a comprehensive list. All items were grouped into three domains: (i) research team and reflexivity, (ii) study design and (iii) data analysis and reporting. Duplicate items and those that were ambiguous, too broadly defined and impractical to assess were removed.

Results. Items most frequently included in the checklists related to sampling method, setting for data collection, method of data collection, respondent validation of findings, method of recording data, description of the derivation of themes and inclusion of supporting quotations. We grouped all items into three domains: (i) research team and reflexivity, (ii) study design and (iii) data analysis and reporting.

Conclusions. The criteria included in COREQ, a 32-item checklist, can help researchers to report important aspects of the research team, study methods, context of the study, findings, analysis and interpretations.

Keywords: focus groups, interviews, qualitative research, research design

For peer review only

Qualitative research explores complex phenomena encountered by clinicians, health care providers, policy makers and consumers in health care. Poorly designed studies and inadequate reporting can lead to inappropriate application of qualitative research in decision-making, health care, health policy and future research.

Formal reporting guidelines have been developed for randomized controlled trials (CONSORT) [1], diagnostic test studies (STARD), meta-analysis of RCTs (QUOROM) [2], observational studies (STROBE) [3] and meta-analyses of observational studies (MOOSE) [4]. These aim to improve the quality of reporting these study types and allow readers to better understand the design, conduct, analysis and findings of published studies. This process allows users of published research to be more fuller informed when they critically appraise studies relevant to each checklist and decide upon applicability of research findings to their local settings. Empiric studies have shown that the use of the CONSORT statement is associated with improvements in the quality of reports of randomized controlled trials [5]. Systematic reviews of qualitative research almost always show that key aspects of study design are not reported, and so there is a clear need for a CONSORT-equivalent for qualitative research [6].

The Uniform Requirements for Manuscripts Submitted to Biomedical Journals published by the International Committee of Medical Journal Editors (ICMJE) do not provide reporting guidelines for qualitative studies. Of all the mainstream biomedical journals (Fig. 1), only the British Medical Journal (BMJ) has criteria for reviewing qualitative research. However, the guidelines for authors specifically record that the checklist is not routinely used. In addition, the checklist is not comprehensive and does not provide specific guidance to assess some of the criteria. Although checklists for critical appraisal are available for qualitative research, there is no widely endorsed reporting framework for any type of qualitative research [7].

We have developed a formal reporting checklist for in-depth interviews and focus groups, the most common methods for data collection in qualitative health research.

Address reprint requests to: Allison Tong, Centre for Kidney Research, The Children's Hospital at Westmead, NSW 2145, Australia. Tel: +61-2-9845-1482; Fax: +61-2-9845-1491; E-mail: allisont@health.usyd.edu.au, allisont@chw.edu.au

© The Author 2007. Published by Oxford University Press on behalf of International Society for Quality in Health Care; all rights reserved

For peer review only - http://bmjopen.bmj.com/site/about/guidelines.xhtml

Downloaded from https://academic.oup.com/intqhc/article/19/6/349/1791966 by guest on 31 October 2021

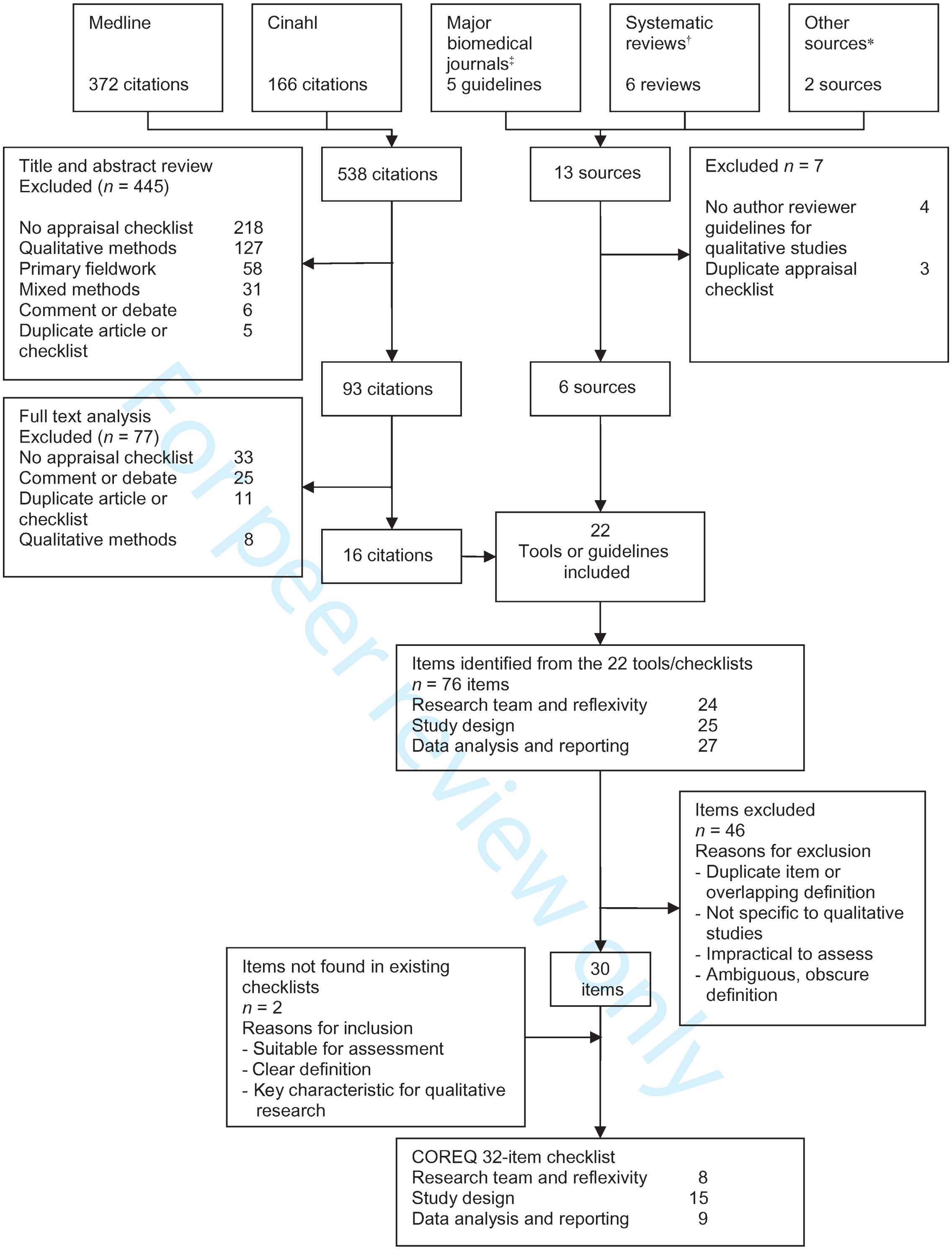

Figure 1 Development of the COREQ Checklist. *References [26, 27], †References [6, 28–32], ‡Author and reviewer guidelines provided by BMJ, JAMA, Lancet, Annals of Internal Medicine, NEJM.

These two methods are particularly useful for eliciting patient and consumer priorities and needs to improve the quality of health care [8]. The checklist aims to promote complete and transparent reporting among researchers and indirectly improve the rigor, comprehensiveness and credibility of interview and focus-group studies.

Basic definitions

Qualitative studies use non-quantitative methods to contribute new knowledge and to provide new perspectives in health care. Although qualitative research encompasses a broad range of study methods, most qualitative research

For peer review only

Downloaded from https://academic.oup.com/intqhc/article/19/6/349/1791966 by guest on 31 October 2021

For peer review only - http://bmjopen.bmj.com/site/about/guidelines.xhtml

publications in health care describe the use of interviews and focus groups [8].

Interviews

In-depth and semi-structured interviews explore the experiences of participants and the meanings they attribute to them. Researchers encourage participants to talk about issues pertinent to the research question by asking open-ended questions, usually in one-to-one interviews. The interviewer might re-word, re-order or clarify the questions to further investigate topics introduced by the respondent. In qualitative health research, in-depth interviews are often used to study the experiences and meanings of disease, and to explore personal and sensitive themes. They can also help to identify potentially modifiable factors for improving health care [9].

Focus groups

Focus groups are semi-structured discussions with groups of 4–12 people that aim to explore a specific set of issues [10]. Moderators often commence the focus group by asking broad questions about the topic of interest, before asking the focal questions. Although participants individually answer the facilitator's questions, they are encouraged to talk and interact with each other [11]. This technique is built on the notion that the group interaction encourages respondents to explore and clarify individual and shared perspectives [12]. Focus groups are used to explore views on health issues, programs, interventions and research.

Methods

Development of a checklist

Search strategy. We performed a comprehensive search for published checklists used to assess or review qualitative studies, and guidelines for reporting qualitative studies in: Medline (1966—Week 1 April 2006), CINAHL (1982—Week 3 April 2006), Cochrane and Campbell protocols, systematic reviews of qualitative studies, author or reviewer guidelines of major medical journals and reference lists of relevant publications. We identified the terms used to index the relevant articles already in our possession and performed a broad search using those search terms. The electronic databases were searched using terms and text words for research (standards), health services research (standards) and qualitative studies (evaluation). Duplicate checklists and detailed instructions for conducting and analysing qualitative studies were excluded.

Data extraction. From each of the included publications, we extracted all criteria for assessing or reporting qualitative studies. Seventy-six items from 22 checklists were compiled into a comprehensive list. We recorded the frequency of each item across all the publications. Items most frequently included in the checklists related to sampling method, setting for data collection, method of data collection, respondent validation of findings, method of recording data, description of the derivation of themes and inclusion of supporting quotations. We grouped all items into three domains: (i) research team and reflexivity, (ii) study design and (iii) data analysis and reporting. (see Tables 2–4)

Within each domain we simplified all relevant items by removing duplicates and those that were ambiguous, too broadly defined, not specific to qualitative research, or impractical to assess. Where necessary, the remaining items were rephrased for clarity. Based upon consensus among the authors, two new items that were considered relevant for reporting qualitative research were added. The two new items were identifying the authors who conducted the interview or focus group and reporting the presence of non-participants during the interview or focus group. The COREQ checklist for explicit and comprehensive reporting of qualitative studies consists of 32 criteria, with a descriptor to supplement each item (Table 1).

COREQ: content and rationale (see Tables 1)

Domain 1: research team and reflexivity

(i) Personal characteristics: Qualitative researchers closely engage with the research process and participants and are therefore unable to completely avoid personal bias. Instead researchers should recognize and clarify for readers their identity, credentials, occupation, gender, experience and training. Subsequently this improves the credibility of the findings by giving readers the ability to assess how these factors might have influenced the researchers' observations and interpretations [13–15].

(ii) Relationship with participants: The relationship and extent of interaction between the researcher and their participants should be described as it can have an effect on the participants' responses and also on the researchers' understanding of the phenomena [16]. For example, a clinician–researcher may have a deep understanding of patients' issues but their involvement in patient care may inhibit frank discussion with patient–participants when patients believe that their responses will affect their treatment. For transparency, the investigator should identify and state their assumptions and personal interests in the research topic.

Domain 2: study design

(i) Theoretical framework: Researchers should clarify the theoretical frameworks underpinning their study so readers can understand how the researchers explored their research questions and aims. Theoretical frameworks in qualitative research include: grounded theory, to build theories from the data; ethnography, to understand the culture of groups with shared characteristics; phenomenology, to describe the meaning and significance of experiences; discourse analysis, to analyse linguistic expression; and content analysis, to systematically organize data into a structured format [10].

For peer review only

Downloaded from https://academic.oup.com/intqhc/article/19/6/349/1791966 by guest on 31 October 2021

Table 1 Consolidated criteria for reporting qualitative studies (COREQ): 32-item checklist

No	Item	Guide questions/description	
**Domain 1: Research team and reflexivity**			
Personal Characteristics			
1.	Interviewer/facilitator	Which author/s conducted the interview or focus group?	Page 7
2.	Credentials	What were the researcher's credentials? *E.g. PhD, MD*	Page 7
3.	Occupation	What was their occupation at the time of the study?	Page 7
4.	Gender	Was the researcher male or female?	Page 7
5.	Experience and training	What experience or training did the researcher have?	Page 7
Relationship with participants			
6.	Relationship established	Was a relationship established prior to study commencement?	Page 7
7.	Participant knowledge of the interviewer	What did the participants know about the researcher? e.g. *personal goals, reasons for doing the research*	Page 7
8.	Interviewer characteristics	What characteristics were reported about the interviewer/facilitator? e.g. *Bias, assumptions, reasons and interests in the research topic*	
**Domain 2: study design**			
Theoretical framework			
9.	Methodological orientation and Theory	What methodological orientation was stated to underpin the study? *e.g. grounded theory, discourse analysis, ethnography, phenomenology, content analysis*	
Participant selection			
10.	Sampling	How were participants selected? *e.g. purposive, convenience, consecutive, snowball*	Page 6
11.	Method of approach	How were participants approached? e.*g. face-to-face, telephone, mail, email*	Page 6
12.	Sample size	How many participants were in the study?	Page 6
13.	Non-participation	How many people refused to participate or dropped out? Reasons?	
Setting			
14.	Setting of data collection	Where was the data collected? e.*g. home, clinic, workplace*	Page 6
15.	Presence of non-participants	Was anyone else present besides the participants and researchers?	
16.	Description of sample	What are the important characteristics of the sample? *e.g. demographic data, date*	Pages 6,7
Data collection			
17.	Interview guide	Were questions, prompts, guides provided by the authors? Was it pilot tested?	Supplemeental materials
18.	Repeat interviews	Were repeat interviews carried out? If yes, how many?	
19.	Audio/visual recording	Did the research use audio or visual recording to collect the data?	Page 7
20.	Field notes	Were field notes made during and/or after the interview or focus group?	
21.	Duration	What was the duration of the interviews or focus group?	Page 7
22.	Data saturation	Was data saturation discussed?	
23.	Transcripts returned	Were transcripts returned to participants for comment and/or correction?	
**Domain 3: analysis and findings**z			
Data analysis			
24.	Number of data coders	How many data coders coded the data?	Page 8
25.	Description of the coding tree	Did authors provide a description of the coding tree?	
26.	Derivation of themes	Were themes identified in advance or derived from the data?	Page 8
27.	Software	What software, if applicable, was used to manage the data?	Page 8
28.	Participant checking	Did participants provide feedback on the findings?	
Reporting			
29.	Quotations presented	Were participant quotations presented to illustrate the themes / findings? Was each quotation identified? e.*g. participant number*	Pages 11 and 12
30.	Data and findings consistent	Was there consistency between the data presented and the findings?	Pages 11 and 12
31.	Clarity of major themes	Were major themes clearly presented in the findings?	Pages 11 and 12
32.	Clarity of minor themes	Is there a description of diverse cases or discussion of minor themes?	

(ii) Participant selection: Researchers should report how participants were selected. Usually purposive sampling is used which involves selecting participants who share particular characteristics and have the potential to provide rich, relevant and diverse data pertinent to the research question [13, 17]. Convenience sampling is less optimal because it may fail to capture important perspectives from difficult-to-reach people [16]. Rigorous attempts to recruit participants and reasons for non-participation should be stated to reduce the likelihood of making unsupported statements [18].

For peer review only

Downloaded from https://academic.oup.com/intqhc/article/19/6/349/1791966 by guest on 31 October 2021

For peer review only - http://bmjopen.bmj.com/site/about/guidelines.xhtml

Table 2 Items included in 22 published checklists: Research team and reflexivity domain

Item	References																					
	[26][a]	[27][a]	[6][b]	[28][b]	[32][b]	[13]	[15]	[14]	[17]	[33]	[34]	[35]	[16]	[19]	[36]	[7]	[37]	[23]	[38]	[39]	[22]	BMJ
Research team and reflexivity																						
Nature of relationship between the researcher and participants		•		•	•		•		•						•				•			
Examination of role, bias, influence	•	•			•	•	•	•							•						•	
Description of role		•		•					•	•				•	•					•	•	
Identity of the interviewer		•		•		•					•		•		•							
Continued and prolonged engagement		•				•							•	•					•	•		
Response to events	•	•				•	•	•														
Prior assumptions and experience		•						•									•			•		
Professional status		•					•								•							
Journal, record of personal experience		•								•				•								
Effects of research on researcher		•				•	•															
Qualifications		•													•							
Training of the interviewer/facilitator			•		•																	
Expertise demonstrated		•																	•			
Perception of research at inception								•						•								
Age							•															
Gender							•															
Social class							•															
Reasons for conducting study		•																				
Sufficient contact													•									
Too close to participants													•									
Empathy																	•					
Distance between researcher and participants							•															
Background								•														
Familiarity with setting																					•	

[a]Other publications, [b]Systematic review of qualitative studies; BMJ, British Medical Journal—editor's checklist for appraising qualitative research); •, item included in the checklist.

Downloaded from https://academic.oup.com/intqhc/article/19/6/349/1791966 by guest on 31 October 2021

Table 3 Items included in 22 published checklists: Study design

Item	[26][a]	[27][a]	[6][b]	[28][b]	[32][b]	[13]	[15]	[14]	[17]	[33]	[34]	[35]	[16]	[19]	[36]	[7]	[37]	[23]	[38]	[39]	[22]	BMJ
Study design																						
Methodological orientation, ontological or epistemological basis		•		•				•	•					•					•	•	•	•
Sampling—convenience, purposive	•	•			•	•	•	•	•	•	•	•	•	•		•		•	•	•	•	•
Setting		•		•	•			•			•				•					•		
Characteristics and description of sample		•			•			•			•		•	•								
Reasons for participant selection	•	•				•		•			•											
Non-participation	•	•		•	•																	
Inclusion and exclusion, criteria		•			•	•													•			
Identity of the person responsible for recruitment				•	•						•				•							
Sample size		•		•	•						•										•	
Method of approach		•									•					•						
Description of explanation of research to participants	•				•										•							
Level and type of participation														•								
Method of data collection, e.g. focus group, in-depth interview	•	•	•	•	•	•		•	•		•	•	•	•		•				•	•	
Audio and visual recording	•	•	•	•	•	•				•	•		•						•		•	•
Transcripts			•	•	•	•			•		•		•						•			•
Setting and location	•	•		•	•		•		•		•				•						•	•
Saturation of data	•	•	•			•			•				•	•							•	
Use of a topic guide, tools, questions	•	•	•								•					•			•	•		
Field notes			•	•	•	•													•			•
Changes and modifications	•	•		•	•														•		•	
Duration of interview, focus group		•				•					•									•		
Sensitive to participant language and views		•										•		•								
Number of interviews, focus groups		•				•																
Time span																					•	
Time and resources available to the study		•																				

[a]Other publications, [b]Systematic review of qualitative studies; BMJ, British Medical Journal—editor's checklist for appraising qualitative research; •, item included in the checklist.

Downloaded from https://academic.oup.com/intqhc/article/19/6/349/1791966 by guest on 31 October 2021

For peer review only

Table 4 Items included in 22 published checklists: Analysis and reporting

Item	References																					
	[26][a]	[27][a]	[6][b]	[28][b]	[32][b]	[13]	[15]	[14]	[17]	[33]	[34]	[35]	[16]	[19]	[36]	[7]	[37]	[23]	[38]	[39]	[22]	BMJ
Respondent validation	•	•	•		•		•		•	•			•	•			•	•	•	•		
Limitations and generalizability	•	•		•	•		•		•		•		•	•				•	•			
Triangulation	•	•		•	•	•	•	•	•					•			•		•			
Original data, quotation		•	•	•	•			•	•		•			•		•				•	•	•
Derivation of themes explicit	•	•	•	•	•		•	•			•								•			•
Contradictory, diverse, negative cases	•	•		•	•		•			•				•					•			•
Number of data analysts	•	•	•			•			•			•	•						•			•
In-depth description of analysis	•			•	•			•			•			•							•	•
Sufficient supporting data presented	•	•		•	•		•				•					•						
Data, interpretation and conclusions linked and integrated		•		•	•							•		•						•		
Retain context of data		•					•	•						•					•			
Explicit findings, presented clearly	•	•		•					•	•												
Outside checks													•	•				•	•			
Software used		•				•													•			•
Discussion both for and against the researchers' arguments	•	•		•	•																	
Development of theories, explanations		•					•			•		•										
Numerical data		•									•							•				•
Coding tree or coding system		•					•												•		•	
Inter-observer reliability		•									•										•	
Sufficient insight into meaning/perceptions of participants		•								•												
Reasons for selection of data to support findings		•			•																	
New insight		•						•														
Results interpreted in credible, innovative way									•													
Eliminate other theories													•									
Range of views														•								
Distinguish between researcher and participant voices								•														
Proportion of data taken into account														•								

[a]Other publications, [b]Systematic review of qualitative studies; BMJ, British Medical Journal—editor's checklist for appraising qualitative research, •, item included in the checklist.

Downloaded from https://academic.oup.com/intqhc/article/19/6/349/1791966 by guest on 31 October 2021

For peer review only

Researchers should report the sample size of their study to enable readers to assess the diversity of perspectives included.

(iii) Setting: Researchers should describe the context in which the data were collected because it illuminates why participants responded in a particular way. For instance, participants might be more reserved and feel disempowered talking in a hospital setting. The presence of non-participants during interviews or focus groups should be reported as this can also affect the opinions expressed by participants. For example, parent interviewees might be reluctant to talk on sensitive topics if their children are present. Participant characteristics, such as basic demographic data, should be reported so readers can consider the relevance of the findings and interpretations to their own situation. This also allows readers to assess whether perspectives from different groups were explored and compared, such as patients and health care providers [13, 19].

(iv) Data collection: The questions and prompts used in data collection should be provided to enhance the readers' understanding of the researcher's focus and to give readers the ability to assess whether participants were encouraged to openly convey their viewpoints. Researchers should also report whether repeat interviews were conducted as this can influence the rapport developed between the researcher and participants and affect the richness of data obtained. The method of recording the participants' words should be reported. Generally, audio recording and transcription more accurately reflect the participants' views than contemporaneous researcher notes, more so if participants checked their own transcript for accuracy [19–21]. Reasons for not audio recording should be provided. In addition, field notes maintain contextual details and non-verbal expressions for data analysis and interpretation [19, 22]. Duration of the interview or focus group should be reported as this affects the amount of data obtained. Researchers should also clarify whether participants were recruited until no new relevant knowledge was being obtained from new participants (data saturation) [23, 24].

Domain 3: analysis and findings

(i) Data analysis: Specifying the use of multiple coders or other methods of researcher triangulation can indicate a broader and more complex understanding of the phenomenon. The credibility of the findings can be assessed if the process of coding (selecting significant sections from participant statements), and the derivation and identification of themes are made explicit. Descriptions of coding and memoing demonstrate how the researchers perceived, examined and developed their understanding of the data [17, 19]. Researchers sometimes use software packages to assist with storage, searching and coding of qualitative data. In addition, obtaining feedback from participants on the research findings adds validity to the researcher's interpretations by ensuring that the participants' own meanings and perspectives are represented and not curtailed by the researchers' own agenda and knowledge [23].

(ii) Reporting: If supporting quotations are provided, researchers should include quotations from different participants to add transparency and trustworthiness to their findings and interpretations of the data [17]. Readers should be able to assess the consistency between the data presented and the study findings, including the both major and minor themes. Summary findings, interpretations and theories generated should be clearly presented in qualitative research publications.

Discussion

The COREQ checklist was developed to promote explicit and comprehensive reporting of qualitative studies (interviews and focus groups). The checklist consists of items specific to reporting qualitative studies and precludes generic criteria that are applicable to all types of research reports. COREQ is a comprehensive checklist that covers necessary components of study design, which should be reported. The criteria included in the checklist can help researchers to report important aspects of the research team, study methods, context of the study, findings, analysis and interpretations.

At present, we acknowledge there is no empiric basis that shows that the introduction of COREQ will improve the quality of reporting of qualitative research. However this is no different than when CONSORT, QUOROM and other reporting checklists were introduced. Subsequent research has shown that these checklists have improved the quality of reporting of study types relevant to each checklist [5, 25], and we believe that the effect of COREQ is likely to be similar. Despite differences in the objectives and methods of quantitative and qualitative methods, the underlying aim of transparency in research methods and, at the least, the theoretical possibility of the reader being able to duplicate the study methods should be the aims of both methodological approaches. There is a perception among research funding agencies, clinicians and policy makers, that qualitative research is 'second class' research. Initiatives like COREQ are designed to encourage improvement in the quality of reporting of qualitative studies, which will indirectly lead to improved conduct, and greater recognition of qualitative research as inherently equal scientific endeavor compared with quantitative research that is used to assess the quality and safety of health care. We invite readers to comment on COREQ to improve the checklist.

References

1. Moher D, Schulz KF, Altman D. The CONSORT statement: revised recommendations for improving the quality of reports of parallel-group randomized trials. *JAMA* 2001;**285**:1987–91.

2. Moher D, Cook DJ, Eastwood S *et al.* Improving the quality of reports of meta-analyses of randomised controlled trials: the QUOROM statement. Quality of Reporting of Meta-analyses. *Lancet* 1999;**354**:1896–900.

Downloaded from https://academic.oup.com/intqhc/article/19/6/349/1791966 by guest on 31 October 2021

For peer review only

3. STROBE Statement: Strengthening the reporting of observational studies in epidemiology. http://www.strobe-statement.org/Checkliste.html

4. Stroup DF, Berlin JA, Morton SC *et al.* Meta-analysis of observational studies in epidemiology: a proposal for reporting. Meta-analysis Of Observational Studies in Epidemiology (MOOSE) group. *JAMA* 2000;**283**:2008–12.

5. Moher D, Jones A, Lepage L. Use of the CONSORT Statement and quality of reports of randomized trials. A comparative before-and-after evaluation. *JAMA* 2001;**285**:1992–5.

6. Mills E, Jadad AR, Ross C *et al.* Systematic review of qualitative studies exploring parental beliefs and attitudes toward childhood vaccination identified common barriers to vaccination. *J Clin Epidemiol* 2005;**58**:1081–8.

7. Knafl KA, Howard NJ. Interpreting and reporting qualitative research. *Res Nurs Health* 1984;**7**:7–14.

8. Sofaer S. Qualitative research methods. *Int J Qual Health Care* 2002;**14**:329–36.

9. Wright EB, Holcombe C, Salmon P. Doctor's communication of trust, care, and respect in breast cancer: qualitative study. *BMJ* 2004;**328**:864–8.

10. Liamputtong P, Ezzy D. *Qualitative Research Methods*. 2nd edn. Melbourne, Victoria: Oxford University Press, 2005.

11. Krueger RA, Casey MA. *Focus Groups. A Practical Guide for Applied Research.* Thousand Oaks CA: Sage Publications, 2000.

12. Morgan DL. *Focus Groups as Qualitative Research.* Newbury Park, California: Sage, 1988.

13. Giacomini MK, Cook DJ. Users' guides to the medical literature XXIII. Qualitative research in health care. A. Are the results of the study valid? *JAMA* 2000;**284**:357–62.

14. Malterud K. Qualitative research:standards challenges guidelines. *Lancet* 2001;**358**:483–8.

15. Mays N, Pope C. Qualitative research in health care: assessing quality in qualitative research. *BMJ* 2000;**320**:50–2.

16. Elder NC, William L. Reading and evaluating qualitative research studies. *J Fam Pract* 1995;**41**:279–85.

17. Cote L, Turgeon J. Appraising qualitative research articles in medicine and medical education. *Med Teach* 2005;**27**:71–5.

18. Altheide D, Johnson J. Criteria for assessing interpretive validity in qualitative research. In Denzin N, Lincoln Y (eds). *Handbook of Qualitative Research.* Thousand Oaks CA: Sage Publications, 1994.

19. Fossey E, Harvey C, McDermott F *et al.* Understanding and evaluating qualitative research. *Aust N Z J Psychiatry* 2002;**36**:717–32.

20. Seale C, Silverman S. Ensuring rigour in qualitative research. *Eur J Public Health* 1997;**7**:379–84.

21. Scheff T. Single case analysis in the health sciences. *Eur J Public Health* 1995;**5**:72–4.

22. Bluff R. Evaluating qualitative research. *Br J Midwifery* 1997;**5**:232–5.

23. Popay J, Rogers A, Williams G. Rationale and standards for the systematic review of qualitative literature in health services research. *Qual Health Res* 1998;**8**:341–51.

24. Blumer H. *Critiques of Research, in the Social Sciences.* New Brunswick, NJ: Transaction Books, 1979.

25. Delaney A, Bagshaw SM, Ferland A *et al.* A systematic evaluation of the quality of meta-anlyses in the critical care literature. *Crit Care* 2005;**9**:575–82.

26. Critical Skills Appraisal Programme (CASP) 10 Questions to help you make sense of qualitative research: Milton Keynes Primary Care Trust, 2002.

27. Spencer L, Ritchie J, Lewis J *et al. Quality in Qualitative Evaluation: A Framework for Assessing Research Evidence.* London: Cabinet Office. Government Chief Social Researcher's Office, 2003.

28. Campbell R, Pound P, Pope C *et al.* Evaluating meta-ethnography: a synthesis of qualitative research on lay experience of diabetes and diabetes care. *Soc Sci Med* 2003;**56**:671–84.

29. Feder GS, Hutson M, Ramsay I *et al.* Women exposed to intimate partner violence: expectations and experiences when they encounter health care professionals: a meta-analysis of qualitative studies. *Arch Intern Med* 2006;**166**:22–37.

30. Pound P, Britten N, Morgan M *et al.* Resisting medicines: a synthesis of qualitative studies of medicine taking. *Soc Sci Med* 2005;**61**:133–55.

31. Smith LK, Pope C, Botha JL. Patients' help-seeking experiences and delay in cancer presentation: a qualitative synthesis. *Lancet* 2005;**366**:825–31.

32. Walter FM, Emery J, Braithwaite D *et al.* Lay understanding of familial risk of common chronic diseases: a systematic review and synthesis of qualitative research. *Ann Fam Med* 2004;**2**:583–94.

33. Inui TS, Frankel RM. Evaluating the quality of qualitative research: a proposal pro-term. *J Gen Intern Med* 1991;**6**:485–6.

34. Boulton M, Fitzpatrick R, Swinburn C. Qualitative research in health care: II A structured review and evaluation of studies. *J Eval Clin Pract* 1996;**2**:171–9.

35. Dixon-Woods M, Shaw RL, Agarwal S *et al.* The problem of appraising qualitative research. *Qual Saf Health Care* 2004;**13**:223–5.

36. Hoddinott P, Pill R. A review of recently published qualitative research in general practice. More methodological questions than answers? *Fam Pract* 1997;**14**:313–9.

37. Kuzel AJ, Engel JD, Addison RB *et al.* Desirable features of qualitative research. *Fam Pract Res J* 1994;**14**:369–78.

38. Treloar C, Champness S, Simpson PL *et al.* Critical appraisal checklist for qualitative research studies. *Indian J Pediatr* 2000;**67**:347–51.

39. Cesario S, Morin K, Santa-Donato A. Evaluating the level of evidence in qualitative research. *J Obstet Gynecol Neonatal Nurs* 2001;**31**:708–14.

Accepted for publication 7 July 2007

For peer review only

Downloaded from https://academic.oup.com/intqhc/article/19/6/349/1791966 by guest on 31 October 2021

BMJ Open

Care seeking for under-five children and vaccine perceptions during the first two waves of the COVID-19 pandemic in Lagos State, Nigeria: a qualitative exploratory study

Journal:	*BMJ Open*
Manuscript ID	bmjopen-2022-069294.R1
Article Type:	Original research
Date Submitted by the Author:	16-Jan-2023
Complete List of Authors:	Bakare, Ayobami; Karolinska Institutet, Department of Global Public Health; University College Hospital, Department of Community Medicine<br>Olojede, Omotayo; University College Hospital Ibadan, Department of Paediatrics<br>King, Carina; Karolinska Institute<br>Graham, Hamish; Centre for International Child Health, University of Melbourne, MCRI, Royal Children's Hospital; University College Hospital Ibadan, Department of Paediatrics<br>Uchendu, Obioma; University College Hospital Ibadan, Department of Community Medicine; University of Ibadan College of Medicine, Department of Community Medicine<br>Colbourn, Timothy; UCL Institute for Global Health<br>Falade, Adegoke; University College Hospital Ibadan, Department of Paediatrics; University of Ibadan College of Medicine, Department of Paediatrics<br>Alvesson, Helle; Karolinska Universitetssjukhuset, Global Public Health
**Primary Subject Heading**:	Public health
Secondary Subject Heading:	Qualitative research
Keywords:	COVID-19, Community child health < PAEDIATRICS, PREVENTIVE MEDICINE, PRIMARY CARE, PUBLIC HEALTH

SCHOLARONE™
Manuscripts

I, the Submitting Author has the right to grant and does grant on behalf of all authors of the Work (as defined in the below author licence), an exclusive licence and/or a non-exclusive licence for contributions from authors who are: i) UK Crown employees; ii) where BMJ has agreed a CC-BY licence shall apply, and/or iii) in accordance with the terms applicable for US Federal Government officers or employees acting as part of their official duties; on a worldwide, perpetual, irrevocable, royalty-free basis to BMJ Publishing Group Ltd ("BMJ") its licensees and where the relevant Journal is co-owned by BMJ to the co-owners of the Journal, to publish the Work in this journal and any other BMJ products and to exploit all rights, as set out in our licence.

The Submitting Author accepts and understands that any supply made under these terms is made by BMJ to the Submitting Author unless you are acting as an employee on behalf of your employer or a postgraduate student of an affiliated institution which is paying any applicable article publishing charge ("APC") for Open Access articles. Where the Submitting Author wishes to make the Work available on an Open Access basis (and intends to pay the relevant APC), the terms of reuse of such Open Access shall be governed by a Creative Commons licence – details of these licences and which Creative Commons licence will apply to this Work are set out in our licence referred to above.

Other than as permitted in any relevant BMJ Author's Self Archiving Policies, I confirm this Work has not been accepted for publication elsewhere, is not being considered for publication elsewhere and does not duplicate material already published. I confirm all authors consent to publication of this Work and authorise the granting of this licence.

For peer review only

Title: Care seeking for under-five children and vaccine perceptions during the first two waves of the COVID-19 pandemic in Lagos State, Nigeria: a qualitative exploratory study

Authors: Ayobami A Bakare[1,2]*, Omotayo E Olojede[3], Carina King[2], Hamish R Graham[4], Obioma Uchendu[1,5], Tim Colbourn[6], Adegoke G Falade[3,7], Helle Mölsted Alvesson[2] on behalf of the INSPIRING Project Consortium**

Affiliations

[1] Department of Community Medicine, University College Hospital, Ibadan, Nigeria.

[2] Department of Global Public Health, Karolinska Institutet, Stockholm, Sweden.

[3] Department of Paediatrics, University College Hospital, Ibadan, Nigeria.

[4] Centre for International Child Health, Murdoch Children's Research Institute, University of Melbourne, Royal Children's Hospital, Parkville, Victoria, Australia.

[5] Department of Community Medicine, College of Medicine, University of Ibadan, Ibadan, Nigeria.

[6] Institute for Global Health, University College London, London, UK.

[7] Department of Paediatrics, College of Medicine, University of Ibadan, Ibadan, Nigeria.

***Corresponding author**

bakare.ayobami.adebayo@ki.se

Department of Global Public Health, Karolinska Institutet, Stockholm.

****Full INSPIRING consortium:** Carina King (Karolinska Institutet); Tim Colbourn, Rochelle Ann Burgess, Agnese Iuliano (UCL); Hamish Graham (Melbourne); Eric McCollum (Johns Hopkins); Tahlil Ahmed, Samy Ahmar, Christine Cassar, Paula Valentine (Save the Children UK); Adamu Isah, Adams Osebi, Magama Abdullahi, Ibrahim Haruna (Save the Children Nigeria); Temitayo Folorunso Olowookere (GSK Nigeria); Matt MacCalla (GSK UK); Adegoke Gbadegesin Falade, Ayobami Adebayo Bakare, Obioma Uchendu, Julius Salako, Funmilayo Shittu and Damola Bakare, Omotayo Olojede (University of Ibadan).

Keywords: COVID-19, lockdown, under-five children, care-seeking, Nigeria

Word count: 4703 words (excluding declarations, references and tables)

For peer review only - http://bmjopen.bmj.com/site/about/guidelines.xhtml

ABSTRACT (279/300)

Objective

To explore health care-seeking practices for children and the context-specific direct and indirect effects of public health interventions during the first two waves of COVID-19 in Lagos State, Nigeria. We also explored decision making around vaccine acceptance at the start of COVID-19 vaccine roll-out in Nigeria

Design, setting and participants

A qualitative explorative study involving 19 semi-structured interviews with healthcare providers from public and private primary health facilities and 32 interviews with caregivers of under-five children in Lagos, from December 2020 to March 2021. Participants were purposively selected from healthcare facilities to include community health workers, nurses, and doctors, and interviews were conducted in quiet locations at facilities. A data-driven reflexive thematic analysis according to Braun & Clark 2019 was conducted.

Findings

Two themes were developed: appropriating COVID-19 in belief systems, and ambiguity about COVID-19 preventive measures. The interpretation of COVID-19 disease ranged from fearful to considering it as a 'scam' or 'falsification from the government'. Underlying distrust in government fueled COVID-19 misperceptions. Care seeking for children under-five was affected, as facilities were seen as contagious places for COVID-19. Caregivers resorted to alternative care and self-management of childhood illnesses. COVID-19 vaccine hesitancy was a major concern among healthcare providers compared to community members at the time of vaccine roll-out in Lagos, Nigeria. Indirect impacts of COVID-19 lockdown included diminished household income, worsening food insecurity, mental health challenges for caregivers and reduced clinic visits for immunization.

Conclusion: The first wave of the COVID-19 pandemic in Lagos was associated with reductions in care seeking for children, clinic attendance for childhood immunizations, and household income. Strengthening health and social support systems with context-specific interventions and containing misinformation is crucial to building adaptive capacity for response to future pandemics.

For peer review only - http://bmjopen.bmj.com/site/about/guidelines.xhtml

Strengths and limitations of this study

- A key strength of this study was the inclusion of perspectives from both caregivers and healthcare providers in private and public health facilities, and the recruitment of various cadres of healthcare providers
- The use of semi-structured interviews, conducted while the pandemic was on-going, provided the opportunity to understand individual perspectives and experiences
- Perspectives captured in this study may have missed some negative impacts of COVID-19 on care seeking given caregivers were recruited from health facilities, and may therefore differ from the wider community
- Findings from this study may not reflect all aspects considered important to the participants as communities and healthcare workers were not consulted in the design of the interview guides

For peer review only

For peer review only - http://bmjopen.bmj.com/site/about/guidelines.xhtml

INTRODUCTION

The COVID-19 pandemic was declared a public health emergency of international concern in January 2020 by the World Health Organization.[1] Differential negative impacts have been reported across the globe due to the COVID-19 pandemic. While some countries have reported a high number of deaths due to COVID-19, others particularly in sub-Sahara Africa have reported low mortality,[2] but have suffered significant social and economic impacts with recovery, likely to take a protracted course.[3] As of March 27, 2022, over eight million cases and 170,000 deaths had been reported in Africa, although estimates of actual cases (505.6 million) and deaths (439,500) in the region are much higher.[4,5] Within Africa, Nigeria reported the fourth highest number of COVID-19 cases in 2020-2021, with 215,164 reported cases (3.4% of the African total) and 92 million estimated cases.[6]Lagos State was the epicentre of the COVID-19 pandemic in Nigeria during this period, accounting for more than 30% of Nigeria's reported cases, with the first cases identified in late February 2020. [7,8]

The pandemic has been a major stressor to health systems, exposing and exacerbating pre-existing fragility and inequities within the system. [9,10] Given the absence of effective and widely available COVID-19 treatments during the first and second waves—February-October 2020 and November 2020-April 2021 respectively, [11] containment measures were based on public health measures like movement and travel restrictions (i.e. 'lockdowns'), physical distancing, personal hygiene and use of personal protective equipment (PPE). [12] Negative impacts of these containment measures on social life and mental well-being, education, economy, health service delivery and utilization have been reported, but mostly from non-empirical data and outside the African context.[13–18] Early predictions of Africa being worst hit by the COVID-19 pandemic did not come to fruition,[19] underscoring the need for context-specific empirical data. While the direct clinical impact of COVID-19 has affected adults more directly in this period, children are not exempt from indirect effects of mitigations, although observed data from Africa is lacking. [20,21]

In March 2020, the Nigerian government imposed several public health measures. The initial COVID-19 pandemic wave in Nigeria was characterized by fear, confusion and instability in the existing social structures, with misinformation fueled by social media reports and lockdown measures imposed by the government.[7,22–25] These may have had knock-on effects on healthcare service utilization and delivery. While multiple studies, largely from high-income contexts, have reported reductions in child illnesses and hospital admissions during periods of COVID-19 restrictions, fewer have explored the role of changes in care-seeking behaviour for children during this period and their implications for future public health responses to disease outbreaks.[16,26]

In Nigeria, under-five mortality remains high, and is not on-track to meet the 2030 Sustainable Development Goal global target of having less than 25 deaths per 1000 live births.[27] Pneumonia, malaria and diarrhoea are leading causes of under-five deaths in the country, responsible for almost 40% of under-five deaths in 2018.[28] Nigeria also experiences multiple outbreaks of diseases of public health significance annually, including meningococcal disease, Yellow fever, and Lassa fever. [29] Given the existing burden of pneumonia, malaria, and diarrhoea among children, the magnitude of the COVID-19 pandemic and response, and the frequency of disease outbreaks requiring public health response which may require mass vaccination, it is important to understand how the COVID-19 pandemic affected care-seeking for under-five children as well as decision making around vaccine introduction for outbreak control. We therefore aimed to understand care-seeking practices for young children and the context-specific direct and indirect effects of public health interventions during the first two waves of COVID-19 pandemic and decision making around vaccine acceptance at the start of COVID-19 vaccine roll-out in Lagos State, Nigeria.

For peer review only - http://bmjopen.bmj.com/site/about/guidelines.xhtml

METHODS

Study design

This was an exploratory qualitative study using reflexive thematic analysis according to Braun & Clark.[30] We conducted semi-structured interviews with caregivers of children under-five and healthcare providers to gather perspectives on care-seeking practices during the first two waves of the COVID-19 pandemic in Lagos State, Nigeria (February-October 2020 and November 2020-April 2021). The study was conducted as part of the process evaluation of the Lagos INSPIRING project, which is evaluating a child pneumonia health system intervention (study registration: ACTRN12621001071819). We followed the Consolidated Criteria for Reporting Qualitative Research (COREQ) guidelines for reporting.[31]

Study setting

The study was conducted in Ikorodu Local Government Area (LGA) in Lagos State. Lagos is the most populous state in Nigeria with an estimated population of 24.6 million people in 2022, [32] and is an economic hub in West Africa. Ikorodu is one of five administrative divisions of Lagos. It is a peri-urban area, with fishing as the predominant economic activity in the rural parts of the LGA, and small and medium scale entrepreneurship as the major economic activity in the urban parts of the LGA. The LGA is served by two government-owned secondary health facilities (General Hospitals), 28 primary healthcare centers (PHCs) and over one hundred private facilities. Of the 28 PHCs, seven are designated as 'flagship' facilities by the Lagos State government, as they have more personnel and equipment and run 24-hour services for children and adults. There is at least one flagship PHC in each of Ikorodu's six Local Council Development Areas (LCDAs) and all of them remained open during the first two waves of the pandemic. The flagship PHCs also acted as COVID-19 vaccination centres, except one facility which did not have a medical doctor.

As part of the public health measures, Lagos was placed on lockdown by the Federal Government of Nigeria on the 30th March 2020.[7] The lockdown lasted 35 days and included a ban on social and economic activities, restriction of all non-essential movements, suspension of commuter services, closure of schools and retail shops and prohibition of mass gatherings except for funeral services.[33] Unlike PHCs and private health facilities, service provisions were limited to emergency cases in the public secondary-level facilities A gradual easing of the lockdown commenced from the 4th May 2020 with no re-instatement of movement restrictions during the second wave (see Appendix I).[7] In addition, there was a period of civil unrest in Lagos, including Ikorodu LGA (the 'EndSARS' protests against police brutality [34]), between 8th and 22nd October 2020, when a curfew was imposed.

Study participants and sampling

We purposively selected healthcare providers who attended to sick children from the seven flagship PHCs and six nearby private facilities (Table 1). To ensure representation of each cadre of healthcare provider, the categories of staff targeted for recruitment (nurse, community health workers, and doctors) was adapted to each facility. We recruited caregivers of children under-five years presenting at the outpatient departments (i.e. with an illness) or immunization clinics (i.e. healthy children) of seven flagship PHCs and one secondary hospital. Caregivers were recruited by female clinical project staff, who screened every child brought to outpatient departments of the facilities for pneumonia. In each facility, we used convenience sampling to recruit four caregivers of under-five children at random (n=32): two caregivers of an acutely unwell child (from outpatients) and two caregivers of a child with no current illness episode (from the immunization clinic). This sample size was based on practical considerations of the time needed to recruit participants and the expectation that it would be sufficient numbers to achieve saturation. All participants approached for the study agreed to take part.

For peer review only - http://bmjopen.bmj.com/site/about/guidelines.xhtml

Table 1 Summary of participants' characteristics

**Caregivers n=32**		**Healthcare providers n=19**	
**Gender**		**Gender**	
Male	0 (0.0)	Male	5 (26.0)
Female	32 (100.0)	Female	14 (74.0)
Mean age (±SD)	31±5.0 years	Mean age (±SD)	38±8.1 years
**Median no of children (range)**	2 (1-5)	**Median year of experience**	11 (2-40)
**Educational level**		**Educational level**	
Primary	2 (6.3)	Diploma	9 (47.4)
Secondary	13 (40.6)	Tertiary	9 (47.4)
Tertiary	17 (53.1)	Postgraduate	1 (5.2)
**Religion**		**Religion**	
Christianity	25 (78.1)	Christianity	15 (78.9)
Islam	7 (21.9)	Islam	4 (21.1)
**Occupation/Cadre**		**Occupation/Cadre**	
Self-employed	21 (64.5)	Doctor	7 (36.8)
Employed	5 (16.1)	Nurse	6 (31.6)
No employment	6 (19.4)	CHEW	6 (31.6)

CHEW, Community Health Extension Worker;

Data collection

Interviews were conducted from 10 December 2020 to 18 March 2021. The semi-structured interview guides were based on the literature on care-seeking practices and knowledge about COVID-19 during the INSPIRING project formative phase and revised to capture the emerging COVID-19 vaccine programme roll-out in Nigeria. The interview guide for caregiver interviews had three sections, focused on: participants' family and socio-demographic information, their experiences of 2020 in light of COVID-19 including their perception of the illness and economic impacts, and care-seeking practices for children under-five years. The interview guide for healthcare provider interviews had three sections focusing on: service provision, facility adaptation to the COVID-19 pandemic, and care seeking for sick under-five children (Appendices II-IV).

The research team was comprises of pediatricians, social science and public health specialists. The interviews were conducted by OEO, a male Master's student from Nigeria with experience of the local context, with support from the female clinical study staff who recruited participants based at each facility.. Interviews were conducted in English or Yoruba (the indigenous local language in Ikorodu LGA), depending on the participant preference. The interviewer lived in Ikorodu before and during the COVID-19 pandemic and had previously visited the participating health facilities for other data collection activities.[35] Caregivers' interviews were conducted at the health facility or in another convenient place agreed by the participants. Providers' interviews were held at the facility. Each interview lasted between

For peer review only - http://bmjopen.bmj.com/site/about/guidelines.xhtml

30-40 minutes and no repeat interviews were carried out. All interviews were voice-recorded, transcribed and translated into English, before being stored in a secure cloud platform with access granted to only research team members. No transcripts were returned to the participants for review.

Data analysis

After cross-checking of the transcripts, the analysis team (AAB, OEO, HMA and CK) conducted a data-driven thematic analysis to develop themes and subthemes.[36] AAB and OEO independently reviewed all the transcripts to identify initial codes which were reconciled in NVivo. [37] Healthcare provider and caregiver interviews were initially coded separately, and then reviewed by the analysis team to identify common themes and sub-themes, which were refined in subsequent analysis meetings. The process continued till the patterns of meaning were clear. The unit of analysis was COVID-19 related responses in the interviews.

Patient and Public Involvement

The overarching study was designed through a co-design workshop involving representatives from the Nigerian governments, community-based organizations, professionals, Save the Children and evaluation partners. However, patients were not involved in the design of this study. Findings from this study were not discussed with the participants, but will be incorporated into the final report that will be disseminated to the relevant stakeholders including healthcare providers and community-based organizations. [38]

FINDINGS

We identified two overarching themes which were common to caregivers and healthcare workers: appropriating COVID-19 in the belief systems, and ambiguity towards preventive measures (Table 2). When the findings differ between healthcare providers and caregivers, this is specifically noted in the text.

Table 2: Summary of themes and sub-themes

**Organizing themes**	**Themes**	**Sub-themes**
Appropriating COVID-19 in the belief systems	Political placement of COVID-19	Disbelief in the virus' existence
		Misinformation and misconceptions about COVID-19
	Socio-theological placement of COVID-19	Religious explanation for COVID-19
		Social placement of COVID-19
	Medical placement of	COVID-19 infection is real

For peer review only - http://bmjopen.bmj.com/site/about/guidelines.xhtml

**Organizing themes**	**Themes**	**Sub-themes**
	COVID-19	Healthcare as a source of infection
Ambiguity about COVID-19 preventive measures	Unappealing lockdown experiences and associated adaptive mechanisms	Direct impact of lockdown
		Indirect impact of lockdown
		Health system adaption and its consequences
	Drivers of COVID-19 vaccine hesitancy	Misinformation and conspiracy theories about COVID-19 vaccine
		Fear and worries about COVID-19 vaccines
		Distrust in government efforts regarding COVID-19 vaccines
		Media influence on COVID-19
	Drivers of COVID-19 vaccine uptake	Motivation to accept COVID-19 vaccine among healthcare providers
		Motivation to accept COVID-19 among community members or caregivers

For peer review only - http://bmjopen.bmj.com/site/about/guidelines.xhtml

Appropriating COVID-19 in the belief systems

This first theme elucidates plurality in the placement of COVID-19 within the context of existing belief systems. Caregivers and healthcare providers ascribed various causes to the emergence of COVID-19 including political, religious, social and geographical dimensions.

From the healthcare providers interviews, social and political placements of COVID-19 emergence were commonly reported. To some healthcare providers, COVID-19 was not perceived as a public health problem in Nigeria.

"Except that they would say that I am a medical practitioner but I still have the impression that there is no COVID in Nigeria. Don't mind me, it's just my own belief. (Doctor—male, public facility)

The COVID-19 pandemic was framed through a political lens, with distrust in the government shaping disbelief in the disease. This distrust in government provided an opening for misinformation about the virus and control measures with participants describing COVID-19 as "a lie" and "a deceit from the government". The distrust also fed into caregivers' perceptions about COVID-19 surveillance, with some caregivers reportedly delaying care seeking to avoid being automatically added to the COVID-19 daily government case list. The disbelief of the existence of COVID-19 had social associations with participants believing that the disease would not affect 'the poor' or 'black man'.

"There were some people that were like nothing is happening, we've not seen someone with it here, none of our relatives had it so it's just a scam. They don't believe it, most people don't believe it". (CHEW—female, public facility)

To some caregivers, COVID-19 was symbolic and they offered religious explanations, describing it as a test of faith, signs of the 'end of time', a "punishment from God" or the "work of the devil", but this was not apparent among healthcare providers

"It's just like God wanted to deliberately punish people for their bad behaviours [...]. Before, when one is sick, they'll say they should carry the individual, if it's our governors, they'll take flight and fly them out of the country. But when COVID-19 came, no one can come inside or go outside. Everyone is static (immobile in lockdown), so it's not COVID-19 again. It's God's judgement on us." (Mother—sick child, 1 child)

Other participants believed that COVID-19 existed as a symptomatic disease caused by a medical germ. Healthcare facilities were described as "contagious" - a source of infection, and hospital avoidance during the acute phase of the pandemic was reported by both caregivers and healthcare providers. Given health facilities were considered high-risk places, this perception resulted in (i) no care-seeking practices for some sick under-five children as caregivers resorted to self-treatment of their child's illness by seeking care from drug sellers instead, and (ii) delayed presentation at health facilities when the child's condition had worsened. Similarly, when caregivers identified COVID-19 signs in their child they avoided hospital for fear of COVID-19 diagnosis or referral to isolation.

They didn't come. A lot of people were practicing self-medication. People who had cough for example, they didn't come for treatment for fear of being told they had COVID. They kept managing it at home. (CHEW—female, public facility)

"Like one of my neighbours when her baby was running a temperature, she could not bring the baby to the hospital because she said when she goes to the hospital - now they will say her baby have this thing high fever, they should take him to isolation center. Because of that she now went to the pharmacy and brought some (medicine)" (Mother—healthy child, 3 children)

Both caregivers and healthcare workers reported being extra-careful in hospital settings, and sometimes this led to inaccessibility of care if healthcare providers suspected COVID-19 or had inadequate protective equipment. In contrast, one healthcare provider noted that service delivery for children did not change, stating that COVID-19 infections in children are not as severe as that of adults, and it would be unethical to deny children access to healthcare.

For peer review only - http://bmjopen.bmj.com/site/about/guidelines.xhtml

Ambiguity about COVID-19 preventive measures

This theme details various responses, experiences, and effects of recommended COVID-19 preventive measures and associated adaptations.

The lockdown was perceived as an unpleasant and difficult period as participants were restricted to indoor livelihoods with little or no access to transportation. Caregivers reported indirect effects of lockdown that could affect care-seeking, including diminished household incomes which necessitated loan acquisition or seeking help from family members. Household food insecurity was exacerbated, and caregivers reported reducing their consumption to save food for their children. There was avoidance of social functions, mental health challenges and a focus on basic needs:

"I have two teachers in my compound, not government teachers but private teachers. When the lockdown started then, the man is a teacher in private school, the woman is a teacher in a private school. As the school was not open, no salary, no money, nothing, nothing. For them to feed was problem, [never mind] if the baby falls sick, and now there is no money to take the baby to hospital. Sometimes, they will go and do herbal, this thing agbo (herbal concoction)" (Mother—healthy child, 3 children)

Health facilities made adjustments to ensure continuous service delivery without undermining safety. Face-masking, physical distancing, and improved personal hygiene were adopted; however, they created additional problems such as discomfort (face-masking), denied access to care, or seeking medical advice from people without medical training. Caregivers complied with the rule although there were reports of anger and verbal assaults on healthcare providers when these measures were enforced at the health facilities.

There was a continuation of routine vaccination services during the lockdown, but caregivers' incorrect assumption of PHC closures during the lockdown (secondary facilities were closed to non-emergency cases), compliance with the lockdown order and fear of COVID-19 partly contributed to reduced attendance at the immunization clinic as reported by a CHEW:

"If you remember even on social media (mass media), it was broadcasted that if what you want to do at the hospital is not very important, stay indoors and stay safe. So people adhered to that rule, to the extent that when we went for outreach services, we asked them why they haven't been coming for immunization. Then they will say it's because of the lockdown, and then "corona" stopped us from coming out. They would also claim they don't know that the facility still runs its services" (CHEW—female, public facility)

When COVID-19 vaccines became available in Nigeria, there were mixed perceptions and ambiguity towards them. Among some caregivers, the vaccine was regarded as "a mark of the beast", or a depopulation strategy from Western countries. Religious belief, misinformation and fear of side effects were reasons identified by caregivers for COVID-19 vaccine hesitancy. Healthcare providers, in contrast, expressed distrust in the government and were concerned about vaccine safety, quality, short timeline for vaccine development and the government's aggression towards COVID-19. They believed the vaccines were not tested very well in Nigeria before being approved.

"*That thing (COVID-19 vaccine) is not well tested that's my point. It's supposed to go through a series of tests before allowing it to come into this country. So I cannot even advise anyone to take it.*" (Nurse—female, private hospital)

Social media (WhatsApp, Facebook, Instagram) was identified as a source of misinformation about the vaccine. One healthcare provider queried the decision of the government to accept donated vaccines that are being rejected by other countries, as reported on social media. Similarly, vaccines sent to Nigeria were presumed to be of sub-optimal quality compared to the ones used abroad but this was linked to distrust in governments.

"Some people (healthcare providers) don't want to take it because of the things we have seen on social media that if you take it, it can cause this and that" (CHEW—female, public facility)

For peer review only - http://bmjopen.bmj.com/site/about/guidelines.xhtml

However, some healthcare providers and caregivers had positive perceptions of the vaccine, describing it as beneficial to the recipients, such as preventing sudden death and protecting against the virus. Others also showed trust in the government believing that the government cannot bring vaccines if they are harmful. Some caregivers also expressed willingness to receive the vaccine given that they are utilizing an existing routine immunization programme.

"If the vaccine comes, we know there's a reason why the government brought it. It has a work it wants to accomplish, which is why they want to bring it; we will take it" (Mother—sick child, 4 children)

Perceived higher risk of infection, the possibility of vaccines becoming scarce, a sense of responsibility to clients, motivation from senior colleagues or health managers, and later positive testimonies from recipients, were identified as drivers of uptake among healthcare providers. Being a requirement for overseas travel or pilgrimage, counselling, and public awareness were reported by healthcare providers as drivers of vaccine uptake among community members. Few healthcare providers who had taken the vaccine identified self-reflection and personal inquiry as ways they dealt with the misinformation about the vaccine.

"I heard they were cloning the vaccine in some European countries. That was my fear but when I did my own research. I found out that there is no issue." (Doctor—female, public facility)

Despite the fear and negative perceptions, community members turned out en masse to receive the vaccine, and turnout exceeded expectations, making the supply inadequate.

*We were even surprised. I wasn't expecting people to come out. It was supposed to be a 10 day program […]but we extended further for four weeks or thereabout. People were still coming, we had to tell them that there was no more vaccination. (*Doctor—male, public facility).

DISCUSSION

It is important to understand both community and healthcare workers' perceptions and experiences during the initial COVID-19 waves to adapt the provision of health care services to children during future pandemics. In the Nigerian context, participants reported both direct and indirect effects on care seeking for children, especially during the acute lockdown periods. Both groups of participants interpreted the COVID-19 pandemic through medical, political, social and economic lenses; however religious interpretation of the pandemic was more prominent among caregivers. Care seeking for children under-five was affected in part due to the perception of healthcare settings being contagious, fear of COVID-19 diagnosis, and limited access to transportation. Adapting to seek care from alternative sources for sick children was reported by both groups. COVID-19 vaccine hesitancy was a major issue among healthcare providers, but less so among community members at the time of vaccine roll-out in Lagos. The motivations for vaccine uptake differed between the groups, and social media seemed to play a crucial role in shaping acceptability of the COVID-19 vaccine.

Our study suggests that COVID-19 related misinformation, rooted in a general distrust of government and cutting across every aspect of the COVID-19 response (including vaccine roll-out), had negative influences on care-seeking for children. This resonates with findings elsewhere in Africa and globally that misinformation and misleading interpretations of health information (e.g. daily reporting of cases and deaths from COVID-19 and fear of being counted as a COVID-19 case, assumption of facility closure during the lockdown) contributed to hospital avoidance, [16,39,40] and therefore requires consideration and active management in future outbreaks. [41] Conversely, the diversity in COVID-19 placement could conceivably have positive influences on care seeking. For instance, religious beliefs relating to COVID-19 may provide emotional resilience and motivate caregivers to do everything possible to protect their children.[42] Fear of COVID-19 may similarly motivate caregivers to seek care early and get vaccinated, and even a disbelief in COVID-19 may motivate caregivers to go about business as usual.

For peer review only - http://bmjopen.bmj.com/site/about/guidelines.xhtml

While there were people who did not believe in COVID-19 and/or did not seek care to avoid being caught up in the response (e.g. wanting to avoid isolation centres), some took it seriously and many integrated religious interpretations into their understanding of the disease. A study conducted in Nigeria found that religion and religious institutions, focused on Christianity, could have a negative influence on illness perception and behaviour, but that most Nigerian Christians comfortably integrated religious and physical health domains.[43]Additionally, some religious organizations actively encouraged adherence to COVID-19 preventive measures.[43] These findings highlight the dynamic process of classifying new diseases, as seen in the emergence of Ebola disease,[44] and the need for socio-cultural considerations and community participation in public health planning and communication, as well as active feedback and management of rumours and misinformation during the response. [45,46]

When caregivers decided to seek care for their children, lack of transportation due to lockdown inhibited access. Our finding agrees with an online survey conducted in Nigeria,[47] but contrasts with a study conducted in the Netherlands which reported parental non-deterrence in care seeking for a sick child. [48] Though the nature of illness could have been responsible for this contrasting finding, given the different epidemiological profiles, differences in health systems, COVID-19 related public health measures, as well as better health literacy around COVID-19, also have modulating effects. As reported in the UK, positive experiences from the National Health Service and support from others were positive influencers of care seeking, whereas fear driven by media and community were barriers to parental care seeking. [49] Worsened household income and food security reported during the acute phase of COVID-19 are in keeping with findings in other African countries, and these have the potential to exacerbate child malnutrition and mortality. [50,51] Like in other settings,[52–55] we found evidence suggesting decreased childhood immunization during the lockdown but the extent is unclear as healthcare providers reported using outreach services to vaccinate defaulters.

Healthcare services being considered as high-risk settings for infection influenced care seeking practices for children. Similar to reports in Nigeria and elsewhere, caregivers were avoiding hospitals for fear of contracting COVID-19. [49,56–58] The resultant self-management of childhood illness and decreased healthcare service utilization are in keeping with other studies from Europe and Africa. [57–60] Studies within and outside Nigeria have also reported increased self-medication practice for the prevention and treatment of COVID-19 related symptoms but did not focus on self-medication for children during the pandemic.[61–63] A study conducted in Uganda also found higher neonatal mortality and morbidity during the lockdown.[64] Estimating the impacts of reduced hospital visits, seeking care from alternative sources, delayed hospital visits and increased self-medication for sick children was outside the scope of this study but will be crucial for understanding the indirect effects of COVID-19 public health measures. Nevertheless, our study supports the need for intelligent health communication and flexible approaches to increasing service delivery capacity, such as mobile outreach clinics to maintain health care access for children. [20,65] A study conducted in the UK hypothesized that decreased incidence of childhood illness during the lockdown period contributed to low paediatric admission for common and severe childhood illness during the lockdown; [66] however, hospital avoidance, care seeking from alternative sources and delayed presentation should not be dismissed.

The underlying distrust in government influenced COVID-19 perceptions, and provided the platform for the growing misinformation about the pandemic and this in turn shaped vaccine hesitancy.[67,68] Our findings are in agreement with studies in Nigeria which found that non-adherence to recommended preventive measures for COVID-19 was centered on political distrust, stemming from decades of perceived bad governance. [68,69] The mixed perception towards COVID-19 in Nigeria was therefore not surprising and similar controversies have been reported across several regions globally.[70] In times of uncertainty, a coping strategy is to use religion to provide explanations for strange events, [71]and these may conflict with emerging scientific evidence (particularly as conclusions change with new data) and frustrate containment measures. [72] Our findings support the need for inclusive risk communication for epidemic preparedness and control. Moreover, intervention adaptation to suit local contexts is essential during emergency response to

For peer review only - http://bmjopen.bmj.com/site/about/guidelines.xhtml

epidemics. [45] Early reported cases of COVID-19 in the country were among foreigners and high-profile politicians. Linking COVID-19 results to known public officers could have been responsible for the perception that COVID-19 is a disease of the elite. In addition, limited testing capacity could have driven the perception that COVID-19 is not real, as up to 80% of infected individuals had been reported as mild or asymptomatic.[73]

Interestingly, the demand for COVID-19 vaccine was reportedly higher than anticipated among community members despite negative media reports and conspiracy theories. This finding is consistent with a study conducted by Julio et al. which found higher willingness to receive COVID-19 vaccine in low-and-middle-income countries compared to high income countries in which the survey was done.[74] Our findings support the call for vaccine equity, the need for sustained global partnership, and continuous post-vaccination surveillance to achieve effective global vaccination for COVID-19.[75] The concern about the unprecedented short period to vaccine production and licensing underscores the need for sustained and increased efforts toward control of other communicable diseases like tuberculosis, HIV/AIDS, and pneumonia—not neglecting other diseases because of COVID-19. Considering the background mistrust in government, donation of substandard vaccines, and vaccines with short expiry dates or not valid for travel as well as conditional donation of vaccines feeds into public narratives of lack of trust in COVID-19 vaccines and reinforces conspiracy theories about COVID-19. [76–78] Meanwhile, vaccine hesitancy among healthcare providers requires attention for increased and sustained COVID-19 vaccine coverage in the long term. [79]

This study had limitations, firstly we recruited caregivers from PHCs only and did not gather perspectives from other community members. This may mean that the perspectives captured here underestimates negative effects on care-seeking. More so, given that participants were not consulted in the design of the interview guide, we acknowledge that finding from this study may not reflect all aspects considered important to the participants. Review of facility data shows a considerable decrease in out-patient attendance for children (Appendix V). Our findings have provided context-specific understanding of the indirect and direct effects of COVID-related public health measures and may inform future public health responses to disease outbreaks. Though the implementation of lockdown is context-specific, findings from our study may be transferrable to other low and middle-income countries with a similar weak health system and where distrust of government has been a problem.

CONCLUSION

The interpretation of the emergence of a new disease classification is dynamic and multi-faceted. The COVID-19 pandemic in Lagos had both direct and indirect effects on care-seeking for children. It is plausible that these had negative impacts on morbidity and mortality. Subsequent disease outbreak response requires active management of misinformation and intelligent health communication, including context-specific understanding of social-media messaging and the role of religious institutions. Strengthening health and social support system interventions, notably around ensuring access to healthcare is not negatively affected, is crucial to building adaptive capacity for future disease outbreaks, pandemics and building public trust.

For peer review only

Acknowledgments

We thank the clinical data collectors and facility heads for their support during the data collection, and the caregivers and healthcare workers for giving us their time.

Authors contributions

AAB, OEO, CK and HMA conceived of the study and TC, CK and AGF are grant holders. AAB designed the study. OEO collected the data with oversight from AAB and OCU. AAB and OEO led the analysis, with support from HMA, CK and HG. The manuscript was drafted by AAB with support from OEO, CK and HMA. All authors contributed to revisions and approved the final manuscript.

Competing Interests

SA, TA, CC and PV are employed by Save the Children UK who are part of the partnership funding the research. TFO, MM are employees of GSK, a multinational for-profit pharmaceutical company that produces pharmaceutical products for childhood pneumonia, including a SARS-CoV-2 vaccine, and no direct financial interests in oxygen or pulse oximeter products.

Funding

This work was funded through the GlaxoSmithKline (GSK)-Save the Children Partnership (grant reference: 82603743). Employees of both GSK and Save the Children UK contributed to the design and oversight of the wider INSPIRING study as part of a co-design process but did not take part directly in this sub-study. Any views or opinions presented are solely those of the author/publisher and do not necessarily represent those of Save the Children UK or GSK, unless otherwise specifically stated.

Data Availability Statement

Data are available upon reasonable request. Transcripts of interviews conducted are available in English may be shared based on nature of request to bakare.ayobami.adebayo@ki.se

Ethics Approval

We obtained ethical approvals from the following ethics committees: Lagos State Primary Health Care Board (ref: LS/PHCB/MS/1128/VOL.V1/005), University of Ibadan/University College Hospital (Ref: UI/EC/19/0551) and the University College London (Ref: 3433/005). We obtained informed oral consent from all the participants and conducted the interviews under strict adherence to the study COVID-19 prevention protocol.

For peer review only

References

1. World Health Organization (WHO). Statement on the second meeting of the International Health Regulations (2005) Emergency Committee regarding the outbreak of novel coronavirus (2019-nCoV). Geneva, Switzerland. Published 2020. Accessed December 22, 2022. https://www.who.int/news/item/30-01-2020-statement-on-the-second-meeting-of-the-international-health-regulations-(2005)-emergency-committee-regarding-the-outbreak-of-novel-coronavirus-(2019-ncov)
2. Adams J, MacKenzie MJ, Amegah AK, et al. The conundrum of low covid-19 mortality burden in sub-saharan africa: Myth or reality? *Glob Health Sci Pract*. 2021;9(3):433-443. doi:10.9745/GHSP-D-21-00172
3. Sub-Saharan Africa Exits Recession in 2021 but Recovery Still Vulnerable. Accessed December 8, 2021. https://www.worldbank.org/en/news/press-release/2021/10/06/sub-saharan-africa-exits-recession-in-2021-but-recovery-still-vulnerable
4. World Health Organization. *COVID-19 Weekly Epidemiological Update*. 85th ed.; 2022. Accessed April 2, 2022. https://www.who.int/publications/m/item/weekly-epidemiological-update-on-covid-19---29-march-2022
5. Waogodo Cabore J, Cyprian Karamagi H, Kipchumba Kipruto H, et al. Articles COVID-19 in the 47 countries of the WHO African region: a modelling analysis of past trends and future patterns. *Lancet Glob Health*. Published online 2022. doi:10.1016/S2214-109X(22)00233-9
6. COVID19_Cases. Accessed December 9, 2021. https://who.maps.arcgis.com/apps/dashboards/0c9b3a8b68d0437a8cf28581e9c063a9
7. Lanre R, Bello K, Olatunde O. Easing of lockdown measures in Nigeria: Implications for the healthcare system. *Health Policy Technol*. 2020;9(January):399-404. https://www.ncbi.nlm.nih.gov/pmc/articles/PMC7490626/
8. Amzat J, Aminu K, Kolo VI, Akinyele AA, Ogundairo JA, Danjibo MC. Coronavirus outbreak in Nigeria: Burden and socio-medical response during the first 100 days. *International Journal of Infectious Diseases*. 2020;98(January):218-224. doi:10.1016/j.ijid.2020.06.067
9. Esmé B, Nick G, Max L, Pablo ARM, Anjela T, and Diego AVP. *The Inequality Virus: Bringing Together a World Torn Apart by coronavirus through a Fair, Just and sustainable Economy*. Vol 1. Oxfam GB; 2021.
10. Jensen N, Kelly AH, Avendano M. The COVID-19 pandemic underscores the need for an equity-focused global health agenda. *Humanit Soc Sci Commun*. 2021;8(1). doi:10.1057/s41599-020-00700-x
11. Akande OW, Elimian KO, Igumbor E, et al. Epidemiological comparison of the first and second waves of the COVID-19 pandemic in Nigeria, February 2020–April 2021. *BMJ Glob Health*. 2021;6(11):e007076. doi:10.1136/BMJGH-2021-007076
12. Odusanya OO, Odugbemi BA, Odugbemi TO, Ajisegiri WS. COVID-19: A Review of the Effectiveness of Non-Pharmacological Interventions. *NIgerian Postgraduate Medical Journal*. 2020;27(4):1-7. doi:10.4103/npmj.npmj
13. Verschuur J, Koks EE, Hall JW. Global economic impacts of COVID-19 lockdown measures stand out in highfrequency shipping data. *PLoS One*. 2021;16(4 April):1-16. doi:10.1371/journal.pone.0248818
14. Ozili PK. COVID-19 Pandemic and Economic Crisis: The Nigerian Experience and Structural Causes. *SSRN Electronic Journal*. Published online 2020:1-19. doi:10.2139/ssrn.3567419

For peer review only - http://bmjopen.bmj.com/site/about/guidelines.xhtml

15. Chakraborty I, Maity P. COVID-19 outbreak: Migration, effects on society, global environment and prevention. *Science of the Total Environment*. 2020;728:138882. doi:10.1016/j.scitotenv.2020.138882
16. Conlon C, McDonnell T, Barrett M, et al. The impact of the COVID-19 pandemic on child health and well-being : Are children " slipping through the net " ? A qualitative study of frontline emergency care staff. *BMC Health Serv Res*. 2021;9(1):1-29. https://bmchealthservres.biomedcentral.com/articles/10.1186/s12913-021-06284-9
17. Impact of COVID-19 on people's livelihoods, their health and our food systems. Accessed March 15, 2022. https://www.who.int/news/item/13-10-2020-impact-of-covid-19-on-people%27s-livelihoods-their-health-and-our-food-systems
18. Passavanti M, Argentieri A, Barbieri DM, et al. The psychological impact of COVID-19 and restrictive measures in the world. *J Affect Disord*. 2021;283(September 2020):36-51. doi:10.1016/j.jad.2021.01.020
19. Berhan Y. Will Africa be Devastated by Covid-19 as Many Predicted? Perspective and Prospective. *Ethiop J Health Sci*. 2020;30(3):459-467. doi:10.4314/ejhs.v30i3.17
20. United Nations. *Policy Brief: The Impacts of COVID-19 on Children*. Vol 109.; 2020. doi:10.1111/apa.15484
21. Ashikkali L, Carroll W, Johnson C. The indirect impact of COVID-19 on child health. *Paediatr Child Health*. 2020;30(12):430-437. doi:10.1016/j.paed.2020.09.004
22. Abayomi O. Olaseni, Akinsola OS, Agberotimi SF, Rotimi Oguntayo. Psychological distress experiences of Nigerians during Covid-19 pandemic; the gender difference. *www.archbronconeumol.org Original*. 2020;(January).
23. Ogoina D. Covid-19 and the rest of us. *Niger Delta Medical Journal*. 2020;4(1):6-8.
24. Obi-Ani NA, Anikwenze C, Isiani MC. Social media and the Covid-19 pandemic: Observations from Nigeria. *Cogent Arts Humanit*. 2020;7(1). doi:10.1080/23311983.2020.1799483
25. Johnson OA, Olaniyi SF, John S, et al. "Infodemic" in a pandemic: COVID-19 Conspiracy Theories in an African Country. *Social Health and Behavior*. Published online 2020:19-24. doi:10.4103/SHB.SHB
26. Scaramuzza A, Tagliaferri F, Bonetti L, et al. Changing admission patterns in paediatric emergency departments during the COVID-19 pandemic. *Arch Dis Child*. 2020;105(7):704-706. doi:10.1136/archdischild-2020-319397
27. United Nations. *Transforming Our World: The 2030 Agenda for Sustainble Development*.; 2016.
28. WHO and Maternal and Child Epidemiology Estimation Group (MCEE). Global and Regional Child Deaths by Cause. 2018.
29. Testimony J Olumade, Oluwafolajimi A Adesanya, Iyanuoluwa J Fred-Akintunwa, et al. Infectious disease outbreak preparedness and response in Nigeria_ history, limitations and recommendations for global health policy and practice _ Enhanced Reader. *AIMS Public Health*. 2020;7(4):736-757.
30. Braun V, Clarke V. Reflecting on reflexive thematic analysis. *Qual Res Sport Exerc Health*. 2019;11(4):589-597. doi:10.1080/2159676X.2019.1628806
31. Tong A, Sainsbury P, Craig J. Consolidated criteria for reporting qualitative research (COREQ): a 32-item checklist for interviews and focus groups. 2007;19(6):349-357.
32. About Lagos – Lagos State Government. Accessed February 24, 2022. https://lagosstate.gov.ng/about-lagos/

For peer review only - http://bmjopen.bmj.com/site/about/guidelines.xhtml

33. Presidential task force on COVID-19. *Implementation Guidance for Lockdown Policy*. Vol 5.; 2020. http://www.akrabjuara.com/index.php/akrabjuara/article/view/919
34. Lawal RA, Ibrahim K. *#ENDSARS: Effecting Positive Change in Governance in Nigeria BEYOND M a y 2 0 2 1*. www.bbforpeace.org
35. Graham HR, Olojede OE, Bakare AA, et al. Measuring oxygen access: Lessons from health facility assessments in Lagos, Nigeria. *BMJ Glob Health*. 2021;6(8). doi:10.1136/bmjgh-2021-006069
36. Braun V, Clarke V. Qualitative Research in Psychology Using thematic analysis in psychology Using thematic analysis in psychology. *Qual Res Psychol*. 2006;3(2):77-101. http://www.tandfonline.com/action/journalInformation?journalCode=uqrp20%5Cnhttp://www.tandfonline.com/action/journalInformation?journalCode=uqrp20
37. QSR International Pty Ltd. (2020) NVivo (released in March 2020).
38. Graham HR, Olojede OE, Bakare AAA, et al. Pulse oximetry and oxygen services for the care of children with pneumonia attending frontline health facilities in Lagos, Nigeria (INSPIRING-Lagos): Study protocol for a mixed-methods evaluation. *BMJ Open*. 2022;12(5). doi:10.1136/bmjopen-2021-058901
39. Nigeria records chloroquine poisoning after Trump endorses it for coronavirus treatment - CNN. Accessed January 5, 2022. https://edition.cnn.com/2020/03/23/africa/chloroquine-trump-nigeria-intl/index.html
40. What are the myths about the coronavirus in Africa? | World Economic Forum. Accessed January 5, 2022. https://www.weforum.org/agenda/2020/04/debunking-9-popular-myths-doing-the-rounds-in-africa-about-the-coronavirus/
41. Winters M, Oppenheim B, Sengeh P, et al. Debunking highly prevalent health misinformation using audio dramas delivered by WhatsApp: Evidence from a randomised controlled trial in Sierra Leone. *BMJ Glob Health*. 2021;6(11). doi:10.1136/bmjgh-2021-006954
42. Roberto A, Sellon A, Cherry ST, Hunter-Jones J, Winslow H. Impact of spirituality on resilience and coping during the COVID-19 crisis: A mixed-method approach investigating the impact on women. *https://doi.org/101080/0739933220201832097*. 2020;41(11-12):1313-1334. doi:10.1080/07399332.2020.1832097
43. Ossai EC. 'It is the antichrist. Can't you see?' Perceptions of COVID-19 among Nigeria's Christians and the Religion—Health Debate. *https://doi.org/103366/swc20210325*. 2021;27(1):48-64. doi:10.3366/SWC.2021.0325
44. Barry S Hewlett, Richard P. Amola. Cultural Contexts of Ebola in Northern Uganda. *Emerg Infect Dis*. 2003;9(10).
45. Owoyemi A, Okolie EA, Omitiran K, et al. Importance of community-level interventions during the COVID-19 pandemic: Lessons from sub-saharan Africa. *American Journal of Tropical Medicine and Hygiene*. 2021;105(4):879-883. doi:10.4269/ajtmh.20-1533
46. Dash S, Parray AA, de Freitas L, et al. Combating the COVID-19 infodemic: A three-level approach for low and middle-income countries. *BMJ Glob Health*. 2021;6(1). doi:10.1136/bmjgh-2020-004671
47. Briggs D, Kattey K. COVID-19 : Parents ' Healthcare-Seeking Behaviour for their Sick Children in COVID-19 : Parents ' Healthcare-Seeking Behaviour for their Sick Children in Nigeria - An Online Survey. 2020;(September). doi:10.9734/IJTDH/2020/v41i1330344

For peer review only - http://bmjopen.bmj.com/site/about/guidelines.xhtml

48. Tan CD, Lutgert EK, Neill S, et al. Parents ' experiences with a sick or injured child during the COVID- lockdown : an online survey in the Netherlands. Published online 2021:1-7. doi:10.1136/bmjopen-2021-055811
49. Watson G, Pickard L, Williams B, Hargreaves D, Blair M. Do I, don't I?' A qualitative study addressing parental perceptions about seeking healthcare during the COVID-19 pandemic. *Arch Dis Child*. 2021;106(11):1118-1124. doi:10.1136/archdischild-2020-321260
50. Kansiime MK, Tambo JA, Mugambi I, Bundi M, Kara A, Owuor C. COVID-19 implications on household income and food security in Kenya and Uganda: Findings from a rapid assessment. *World Dev*. 2021;137. doi:10.1016/j.worlddev.2020.105199
51. Osendarp S, Akuoku JK, Black RE, et al. The COVID-19 crisis will exacerbate maternal and child undernutrition and child mortality in low- and middle-income countries. *Nat Food*. 2021;2(7):476-484. doi:10.1038/s43016-021-00319-4
52. Emilia Connolly, Emma J Boley, Donald Luke Fejfar, et al. Childhood immunization during the COVID-19 pandemic: experiences in Haiti, Lesotho, Liberia and Malawi. *Bull World Health Organization*. Published online 2022.
53. Zhong Y, Clapham HE, Aishworiya R, et al. Childhood vaccinations: Hidden impact of COVID-19 on children in Singapore. *Vaccine*. 2021;39(5):780-785. doi:10.1016/J.VACCINE.2020.12.054
54. Chandir S, Siddiqi DA, Setayesh H, Khan AJ. Impact of COVID-19 lockdown on routine immunisation in Karachi, Pakistan. *Lancet Glob Health*. 2020;8(9):e1118-e1120. doi:10.1016/S2214-109X(20)30290-4/ATTACHMENT/620A1068-4BDB-41AC-9C3C-14F0A5E00589/MMC1.PDF
55. McDonald HI, Tessier E, White JM, et al. Early impact of the coronavirus disease (COVID-19) pandemic and physical distancing measures on routine childhood vaccinations in England, January to April 2020. *Eurosurveillance*. 2020;25(19):2000848. doi:10.2807/1560-7917.ES.2020.25.19.2000848/CITE/PLAINTEXT
56. Iwuoha VC, Aniche ET, Obiora CA, UMeifekwem UT. Citizens lack access to healthcare facilities_ How COVID-19 lockdown and social distancing policies boost roadside chemist businesses in South-Eastern Nigeria _ Enhanced Reader. *International Journal of Health Planning Management*. Published online 2021.
57. Davis AL, Sunderji A, Marneni SR, et al. Caregiver-reported delay in presentation to pediatric emergency departments for fear of contracting COVID-19: a multi-national cross-sectional study. *Canadian Journal of Emergency Medicine*. 2021;23(6):778-786. doi:10.1007/s43678-021-00174-z
58. Lazzerini M, Barbi E, Apicella A, Marchetti F, Cardinale F, Trobia G. Delayed access or provision of care in Italy resulting from fear of COVID-19. *Lancet Child Adolesc Health*. 2020;4(5):e10-e11. doi:10.1016/S2352-4642(20)30108-5
59. Ciacchini B, Tonioli F, Marciano C, et al. Reluctance to seek pediatric care during the COVID-19 pandemic and the risks of delayed diagnosis. *Ital J Pediatr*. 2020;46(1):1-4. doi:10.1186/s13052-020-00849-w
60. Singh DR, Sunuwar DR, Shah SK, et al. Impact of COVID-19 on health services utilization in Province-2 of Nepal: a qualitative study among community members and stakeholders. *BMC Health Serv Res*. 2021;21(1):1-14. doi:10.1186/s12913-021-06176-y
61. Wegbom AI, Edet CK, Raimi O, Fagbamigbe AF, Kiri VA. Self-Medication Practices and Associated Factors in the Prevention and/or Treatment of COVID-19 Virus: A Population-Based Survey in Nigeria. *Front Public Health*. 2021;9(June):1-9. doi:10.3389/fpubh.2021.606801

For peer review only - http://bmjopen.bmj.com/site/about/guidelines.xhtml

62. Onchonga D, Omwoyo J, Nyamamba D. Assessing the prevalence of self-medication among healthcare workers before and during the 2019 SARS-CoV-2 (COVID-19) pandemic in Kenya. *Saudi Pharmaceutical Journal*. 2020;28(10):1149-1154. doi:10.1016/j.jsps.2020.08.003
63. Osaigbovo L, Ogboghodo E, Obaseki D, et al. Pattern of Drug Sales At Community Pharmacies in Edo State As Evidence of Self-Medication During the Covid-19 Pandemic: Implications for Policy Implementtation. *J Chem Inf Model*. 2020;20(04):150-158.
64. Hedstrom A, Mubiri P, Nyonyintono J, et al. Impact of the early COVID-19 pandemic on outcomes in a rural Ugandan neonatal unit: A retrospective cohort study. *PLoS One*. 2021;16(12 December). doi:10.1371/JOURNAL.PONE.0260006
65. *Maintaining the Provision and Use of Services for Maternal, during the COVID-19 Pandemic: Lessons Learned from 19 Countries*.; 2021. licence: CC BY-NC-SA 3.0 IGO.
66. Kadambari S, Goldacre R, Morris E, Goldacre MJ, Pollard AJ. Indirect effects of the covid-19 pandemic on childhood infection in England: population based observational study. *BMJ*. Published online 2022. doi:10.1136/bmj-2021-067519
67. Agbo UM, Nche GC. Suspecting the Figures: What Church Leaders Think About Government's Commitment to Combating COVID-19 in Nigeria. *J Asian Afr Stud*. Published online 2022. doi:10.1177/00219096211069645
68. Wonodi C, Obi-Jeff C, Adewumi F, et al. Conspiracy theories and misinformation about COVID-19 in Nigeria: Implications for vaccine demand generation communications. *Vaccine*. 2022;40(13):2114-2121. doi:10.1016/J.VACCINE.2022.02.005
69. Ezeibe CC, Ilo C, Ezeibe EN, et al. Political distrust and the spread of COVID-19 in Nigeria. *Glob Public Health*. 2020;15(12):1753-1766. doi:10.1080/17441692.2020.1828987
70. Hardy LJ. *Connection, Contagion, and COVID-19*.
71. Adiyoso W, Kanegae H. The Preliminary Study of the Role of Islamic Teaching in the Disaster Risk Reduction (A Qualitative Case Study of Banda Aceh, Indonesia). *Procedia Environ Sci*. 2013;17:918-927. doi:10.1016/j.proenv.2013.02.110
72. Yau EKB, Ping NPT, Shoesmith WD, James S, Hadi NMN, Lin LJ. The behaviour changes in response to COVID-19 pandemic within Malaysia. *Malaysian Journal of Medical Sciences*. 2020;27(2):45-50. doi:10.21315/mjms2020.27.2.5
73. Wu Z, McGoogan J. Characteristics of and Important Lessons From the Coronavirus Disease 2019 (COVID-19) Outbreak in China: Summary of a Report of 72 314 Cases From the Chinese Center for Disease Control and Prevention. *JAMA*. Published online 2020.
74. Solís Arce JS, Warren SS, Meriggi NF, et al. COVID-19 vaccine acceptance and hesitancy in low- and middle-income countries. *Nat Med*. 2021;27(8):1385-1394. doi:10.1038/s41591-021-01454-y
75. Forman R, Shah S, Jeurissen P, Jit M, Mossialos E. COVID-19 vaccine challenges: What have we learned so far and what remains to be done? *Health Policy (New York)*. 2021;125(5):553-567. doi:10.1016/j.healthpol.2021.03.013
76. Statement on Covishield and the European Union (EU) Digital COVID Certificate "Green Pass" – Africa CDC. Accessed June 27, 2022. https://africacdc.org/download/statement-on-covishield-and-the-european-union-eu-digital-covid-certificate-green-pass/
77. Joint Statement on Dose Donations of COVID-19 Vaccines to African Countries. Accessed June 27, 2022. https://www.who.int/news/item/29-11-2021-joint-statement-on-dose-donations-of-covid-19-vaccines-to-african-countries

For peer review only - http://bmjopen.bmj.com/site/about/guidelines.xhtml

78. Lucien Hordijk, Priti Patnaik. Covid-19-How Europe’s vaccine donations went tragically wrong. doi:10.1136/bmj.o1286

79. Ackah M, Ameyaw L, Gazali M, et al. COVID-19 vaccine acceptance among health care workers in Africa: A systematic review and meta-analysis. *PLoS One*. 2022;17(5):e0268711. doi:10.1371/JOURNAL.PONE.0268711

For peer review only

Appendix I: -Ease of COVID – 19 Lockdown in Nigeria

	Phase 1	Phase 2	Phase 3	Phase 4	Phase 5
**Start date**<br><br>**End date**	4th May 2020<br><br>1st June 2020	2nd June 2020<br><br>18th October 2020	19th October 2020	11th May 2021	2nd April 2022
**Land travel (Interstate)**	Banned except for essential services and movement of goods and services only	Opened under strict conditions:<br><br>Allowed for essential services and movement of goods and services only	Opened	Opened	Opened
**Land travel (Intrastate)**	Limited to 6 am-6 pm with a 50% reduction in bus occupancy	Opened	Opened	Opened	Opened
**Airspace**	Closed<br><br>Opened to cargo and specially approved flights only	Opened for domestic flights, limited for essential international flights until August 26	Opened (domestic and international flights)	Opened for domestic and international	Opened
**Movement**	Curfew from 8pm to 6am	Curfew from 10pm – 4am	12am to 4am	Curfew from 12am – 4am	No restrictions
**Working hours**	9 am to 2 pm	9 am – 2 pm for Government/other corporate offices	All government staff on grade level 12 and below to continue staying at home<br><br>No limit for private and other corporate bodies	All government staff on grade level 12 and below to continue staying at home until 11th June 2021<br><br>No limit for private and other corporate bodies	No limit
**Workspace**	50% staff occupancy or less	75% staff occupancy or less<br>50% for clients	100% occupancy	No limits but virtual meetings and work from home encouraged	No limit
**Entertainment activities**	Banned	Banned	Opened	Open with some restrictions (bars, night clubs, pubs remained closed)	Opened at 50% capacity
**Mass gathering**	Limited to 20 people or less	Limited to 20 people or less	Limited to 50 people or less	Limited to 50 people or less except	Opened

				with permission from the state government	
**Religious gathering**	Restricted	Restricted	Restricted (subject to the protocol from the state government and the federal capital territory	Limited to less than 50% capacity<br><br>Gathering more than 50 people must be held outdoors only	Opened
**Schools**	Closed	Closed, but special consideration for graduation exams	Opened	Opened	Opened
**Markets**	Partial closure, open only on designated days weekly between 8.00 am-3.00 pm	Controlled access by local authorities	Open	Open	Open
**Face masks**	Mandatory for all persons in public spaces	Mandatory for all persons in public spaces	Mandatory for all persons in public spaces	Mandatory for all persons in public spaces	Mandatory for indoor activities only, but at individual discretion for outdoor activities
**Banks and other financial institutions**	Limit staff physically to between 30%-50%	Limit staff physically to 75% or less. To operate normal working hours	To operate normal working hours	To operate normal working hours	To operate normal working hours

Source: NCDC Coronavirus COVID-19 Microsite. Accessed June 29, 2022. https://covid19.ncdc.gov.ng/guideline/

- *There was a total lockdown of economic activities in the FCT, Lagos and Ogun states for 35days from 30th March 2020. This was coupled with a total ban on non-essential interstate travels*
- *From the third phase, the end dates were assumed as the onset of the next phase*
- *Data collection was done during the phase 3*
- *The second wave of infection and vaccine rollout started during the phase 3*

For peer review only

Appendix II: In-depth interview guide for healthcare provider's interviews

1. Tell me about the facility you work in?
    a. What type of services do you offer children?
    b. Tell me specifically about this week in your clinic
2. Think about last 8 months, has things been typical? Why? Why not?
    a. When did you first hear about covid?
    b. When did you make adaptation or adjustment in your facility as a result of covid?
    c. What changes did your facility make?

NOW WE WANT TO FOCUS ON QUESTIONS REGARDING CHILDREN

3. Tell me how the lockdown in year 2020 affected service provision at your facility
    a. How did it affect services you provide for children?
    a. How did it affect care seeking for sick under-five?
        - Probe severity of illness at presentation/late presentation
        - Was the PHC the first point of call?

4. Thinking about this time last year, before covid/EndSARS, is there any differences? What is different (numbers, type of presentation, services provided, resources), what is the same?
5. Now that lockdown is over, have things normalized the way it used to be before COVID-19? What has normalized? What is yet to normalize. What about number of under-five that you see, any difference compared to last year in terms of number, type of presentation
6. Late in last year, there was Endsars protest. How did it affect service delivery in your facility?
7. Currently, there is second wave of Covid-19 in Nigeria. How has it affected service delivery in your facility?
8. What can you say about the care seeking behavior of caregivers of sick children you have attended to in recent times?
9. Between Covid-19 lockdown, End-Sars protest and current economic hardship, which one has affected care seeking for sick children most? Why? Short term consequences? Any long term consequences?
10. Finally, the federal government is making plans to procure Covid-19 vaccines for Nigerians. How willing are you to receive the vaccine? Why/why not? What about for your child/children? Why/why not? Will you tell others to take it? Why?
11. Do you have any other things to say?

For peer review only

Appendix III: In – Depth interview guide for caregiver of under-five with recent illness episode

1. Tell me about your family.
   Probe to get information on:
   a. Who lives with the participant
   b. Participant's job
   c. Where participant's extended family live
   d. Involvement in child's care
2. How will you summarize year 2020?
   Probe:
   a. How did it affect you and your family?
   b. Could you say these changes were due to impact of covid-19 pandemic?
      1. If yes, why? In what other ways have covid-19 affected you and your household
      2. If no, why not?
   c. Have you noticed changes in the price of commodities?
      i. How does this affect you and your household?
         1. House rent
         2. Transport cost
         3. Food items

Now we want to talk about child health services, particularly care seeking for sick under-five

3. Your child was recently sick; I would like to know more about the illness.
   a. How did it start? Who first noticed the symptoms?
   b. What did you do first? When did you do that?
   c. What next did you do?
      i. How did you decide?
      ii. Why did you do that? Could you have done something else?
      iii. What treatment were given? Was your child asked to do some tests? Could you afford all the test?
      iv. Were you referred?
         1. If yes, did you honour the referral? Why?
         2. How did you feel with the referral?
         3. If no, why? What did you do next? Why did you do that?
         4. Was your child asked to do some tests? Could you afford all the test?
         5. What about medications? Did you buy all the medication?
      v. Like how much did it cost you to treat your child? Would the cost have been cheaper if not for current situations? How did you cover the cost of treatments for your child?
         1. Personal money/savings?
         2. Support from father?
         3. Support family and friends?
         4. Did you have to borrow or sell any items?

4. Overall, has covid-19 affected your decisions and steps when your child was sick?

   i. If yes, how?
   ii. If no, what affected your decisions and steps?
      i. Endsars protest?

ii. Insecurity?

iii. Current economic hardship?

iv. Could you have taken different actions/steps (*relate this to previous answers*) What about fears of catching covid at the hospital?

5. Finally, the federal government has indicated that by Jan 2021 the country will have covid-19 vaccine. How willing are you to receive the vaccine? Why/why not? What about for your child/children? Why/why not?
6. Do you have other things to say or bring to my attention? Thank you for your time!

For peer review only

Appendix IV: IDI guide for No illness episode

1. Tell me about your family.
   Probe to get information on:
   e. Who lives with the participant.
   f. Participant's job
   g. Where participant's extended family live
   h. Involvement in child's care
2. How will you summarize year 2020?
   Probe:
   a. How has it affected you and your family?
   b. Were these covid-19 related?
      i. If yes, why and how? In what **other** ways have covid-19 affected you and your household
      ii. If no, what do you think is responsible?
   c. Have you noticed changes in the price of commodities?
      i. How does this affect you and your household?
         1. House rent
         2. Transport cost
         3. Food items
         4. Fuel price
3. Now we want to talk about care seeking for under-five. What actions do mothers/caregivers take when their child develops illness?
      i. Why do they do that?
         1. Could it be because of trust/distrust in health care workers?
         2. Could cost have influenced their decision? How?
         3. What else could have influenced their action?
      ii. Think about the last time your child (or that of someone close to you) fell sick
         1. What was wrong? What did you do? How did you decide on what to do? Who did you talk to? What alternatives were considered? What were your concerns?
         2. Was your child referred?
         3. Did you honour the referral?
         4. If yes, why? If no, why?
         5. If it happens this period, could you or they have taken different action? Why?
      iii. During the covid-pandemic in Nigeria, do you think covid-19 affected decisions taken by caregivers when their child was sick? if yes, why and how? If no, why?
      iv. What about now? Do covid-19 affect actions taken by mothers when their child falls sick?
      v. Between covid-19 and current economic hardship, which one has greater influence on actions taken by caregivers when their child is sick?
         1. Why and how?
4. Finally, the federal government has indicated that by Jan 2021 the country will have covid-19 vaccine. How willing are you to receive the vaccine? Why/why not? What about for your child/children? Why/why not?
5. Do you have any other thing to tell me?

Thank you for your time!

For peer review only

Appendix V Outpatient attendance for under-five children in the 7 flagship facilities in Ikorodu LGA (January-June 2020)*

**Year**	**Flagship PHCs**													
2020	**Ikorodu**		**Igbogbo**		**Odonla**		**Agbede**		**Ipakodo**		**Imota**		**Oke-Eletu¥**	
**January—March**	**Diagnosis**	**Number**	**Diagnosis**	**Number**	**Diagnosis**	**Number**	**Diagnosis**	**Number**	**Diagnosis**	**Number**	**Diagnosis**	**Number**	**Diagnosis**	**Number**
	Pneumonia	9	Pneumonia	3	Pneumonia	1	Pneumonia	0	Pneumonia	3	Pneumonia	19	Pneumonia	-
	LRTI	7	LRTI	12	LRTI	1	LRTI	0	LRTI	1	LRTI	2	LRTI	-
	URTI	133	URTI	290	URTI	89	URTI	0	URTI	102	URTI	47	URTI	-
	ARTI	0	ARTI	0	ARTI	0	ARTI	0	ARTI	5	ARTI	0	ARTI	-
	Malaria	511	Malaria	275	Malaria	149	Malaria	129	Malaria	234	Malaria	125	Malaria	-
	Sepsis	43	Sepsis	97	Sepsis	243	Sepsis	21	Sepsis	42	Sepsis	33	Sepsis	-
	Others	274	Others	374	Others	406	Others	115	Others	211	Others	252	Others	-
	**Total**	**977**	**Total**	**1051**	**Total**	**892**	**Total**	**265**	**Total**	**589**	**Total**	**478**	**Total**	-
**April—June**	Pneumonia	1	Pneumonia	0	Pneumonia	2	Pneumonia	0	Pneumonia	0	Pneumonia	1	Pneumonia	0
	LRTI	3	LRTI	1	LRTI	0	LRTI	0	LRTI	0	LRTI	0	LRTI	0
	URTI	10	URTI	55	URTI	5	URTI	1	URTI	25	URTI	2	URTI	34
	ARTI	0	ARTI	0	ARTI	0	ARTI	0	ARTI	0	ARTI	0	ARTI	0
	Malaria	215	Malaria	183	Malaria	64	Malaria	23	Malaria	39	Malaria	52	Malaria	42
	Sepsis	22	Sepsis	26	Sepsis	55	Sepsis	2	Sepsis	9	Sepsis	8	Sepsis	63
	Others	113	Others	39	Others	159	Others	5	Others	99	Others	125	Others	164
	**Total**	**364**	**Total**	**304**	**Total**	**285**	**Total**	**31**	**Total**	**172**	**Total**	**188**	**Total**	**303**

*Lagos placed on lockdown on the 30 March 2020

¥ Facility register not found

For peer review only

International Journal for Quality in Health Care; Volume 19, Number 6: pp. 349–357 10.1093/intqhc/mzm042
Advance Access Publication: 14 September 2007

Consolidated criteria for reporting qualitative research (COREQ): a 32-item checklist for interviews and focus groups

ALLISON TONG[1,2], PETER SAINSBURY[1,3] AND JONATHAN CRAIG[1,2]

[1]School of Public Health, University of Sydney, NSW 2006, Australia, [2]Centre for Kidney Research, The Children's Hospital at Westmead, NSW 2145, Australia, and [3]Population Health, Sydney South West Area Health Service, NSW 2170, Australia

Abstract

Background. Qualitative research explores complex phenomena encountered by clinicians, health care providers, policy makers and consumers. Although partial checklists are available, no consolidated reporting framework exists for any type of qualitative design.

Objective. To develop a checklist for explicit and comprehensive reporting of qualitative studies (indepth interviews and focus groups).

Methods. We performed a comprehensive search in Cochrane and Campbell Protocols, Medline, CINAHL, systematic reviews of qualitative studies, author or reviewer guidelines of major medical journals and reference lists of relevant publications for existing checklists used to assess qualitative studies. Seventy-six items from 22 checklists were compiled into a comprehensive list. All items were grouped into three domains: (i) research team and reflexivity, (ii) study design and (iii) data analysis and reporting. Duplicate items and those that were ambiguous, too broadly defined and impractical to assess were removed.

Results. Items most frequently included in the checklists related to sampling method, setting for data collection, method of data collection, respondent validation of findings, method of recording data, description of the derivation of themes and inclusion of supporting quotations. We grouped all items into three domains: (i) research team and reflexivity, (ii) study design and (iii) data analysis and reporting.

Conclusions. The criteria included in COREQ, a 32-item checklist, can help researchers to report important aspects of the research team, study methods, context of the study, findings, analysis and interpretations.

Keywords: focus groups, interviews, qualitative research, research design

Qualitative research explores complex phenomena encountered by clinicians, health care providers, policy makers and consumers in health care. Poorly designed studies and inadequate reporting can lead to inappropriate application of qualitative research in decision-making, health care, health policy and future research.

Formal reporting guidelines have been developed for randomized controlled trials (CONSORT) [1], diagnostic test studies (STARD), meta-analysis of RCTs (QUOROM) [2], observational studies (STROBE) [3] and meta-analyses of observational studies (MOOSE) [4]. These aim to improve the quality of reporting these study types and allow readers to better understand the design, conduct, analysis and findings of published studies. This process allows users of published research to be more fuller informed when they critically appraise studies relevant to each checklist and decide upon applicability of research findings to their local settings. Empiric studies have shown that the use of the CONSORT statement is associated with improvements in the quality of reports of randomized controlled trials [5]. Systematic reviews of qualitative research almost always show that key aspects of study design are not reported, and so there is a clear need for a CONSORT-equivalent for qualitative research [6].

The Uniform Requirements for Manuscripts Submitted to Biomedical Journals published by the International Committee of Medical Journal Editors (ICMJE) do not provide reporting guidelines for qualitative studies. Of all the mainstream biomedical journals (Fig. 1), only the British Medical Journal (BMJ) has criteria for reviewing qualitative research. However, the guidelines for authors specifically record that the checklist is not routinely used. In addition, the checklist is not comprehensive and does not provide specific guidance to assess some of the criteria. Although checklists for critical appraisal are available for qualitative research, there is no widely endorsed reporting framework for any type of qualitative research [7].

We have developed a formal reporting checklist for in-depth interviews and focus groups, the most common methods for data collection in qualitative health research.

Address reprint requests to: Allison Tong, Centre for Kidney Research, The Children's Hospital at Westmead, NSW 2145, Australia. Tel: +61-2-9845-1482; Fax: +61-2-9845-1491; E-mail: allisont@health.usyd.edu.au, allisont@chw.edu.au

© The Author 2007. Published by Oxford University Press on behalf of International Society for Quality in Health Care; all rights reserved

For peer review only - http://bmjopen.bmj.com/site/about/guidelines.xhtml

For peer review only

Downloaded from https://academic.oup.com/intqhc/article/19/6/349/1791966 by guest on 31 October 2021

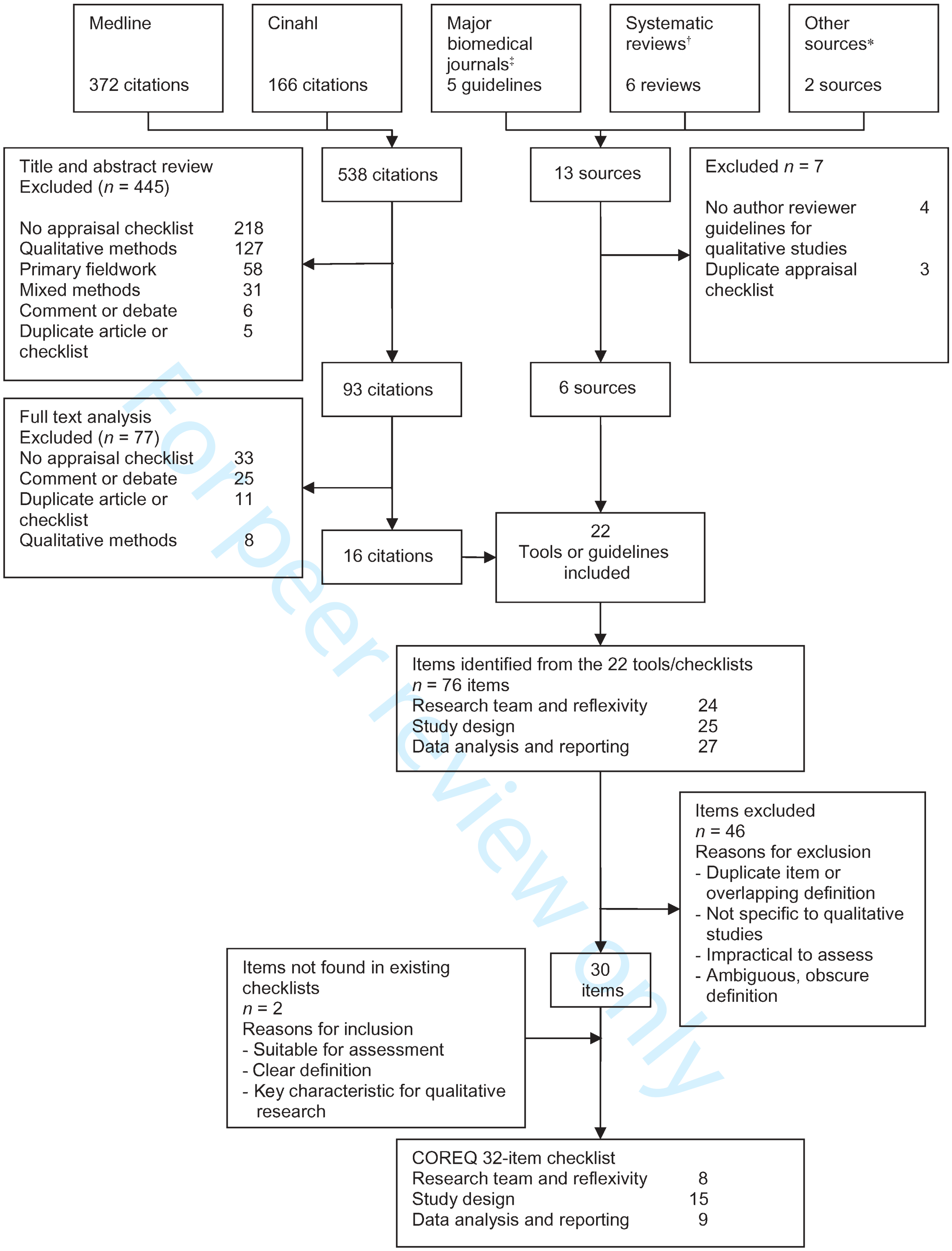

Figure 1 Development of the COREQ Checklist. *References [26, 27], †References [6, 28–32], ‡Author and reviewer guidelines provided by BMJ, JAMA, Lancet, Annals of Internal Medicine, NEJM.

These two methods are particularly useful for eliciting patient and consumer priorities and needs to improve the quality of health care [8]. The checklist aims to promote complete and transparent reporting among researchers and indirectly improve the rigor, comprehensiveness and credibility of interview and focus-group studies.

Basic definitions

Qualitative studies use non-quantitative methods to contribute new knowledge and to provide new perspectives in health care. Although qualitative research encompasses a broad range of study methods, most qualitative research

For peer review only

Downloaded from https://academic.oup.com/intqhc/article/19/6/349/1791966 by guest on 31 October 2021

publications in health care describe the use of interviews and focus groups [8].

Interviews

In-depth and semi-structured interviews explore the experiences of participants and the meanings they attribute to them. Researchers encourage participants to talk about issues pertinent to the research question by asking open-ended questions, usually in one-to-one interviews. The interviewer might re-word, re-order or clarify the questions to further investigate topics introduced by the respondent. In qualitative health research, in-depth interviews are often used to study the experiences and meanings of disease, and to explore personal and sensitive themes. They can also help to identify potentially modifiable factors for improving health care [9].

Focus groups

Focus groups are semi-structured discussions with groups of 4–12 people that aim to explore a specific set of issues [10]. Moderators often commence the focus group by asking broad questions about the topic of interest, before asking the focal questions. Although participants individually answer the facilitator's questions, they are encouraged to talk and interact with each other [11]. This technique is built on the notion that the group interaction encourages respondents to explore and clarify individual and shared perspectives [12]. Focus groups are used to explore views on health issues, programs, interventions and research.

Methods

Development of a checklist

Search strategy. We performed a comprehensive search for published checklists used to assess or review qualitative studies, and guidelines for reporting qualitative studies in: Medline (1966—Week 1 April 2006), CINAHL (1982—Week 3 April 2006), Cochrane and Campbell protocols, systematic reviews of qualitative studies, author or reviewer guidelines of major medical journals and reference lists of relevant publications. We identified the terms used to index the relevant articles already in our possession and performed a broad search using those search terms. The electronic databases were searched using terms and text words for research (standards), health services research (standards) and qualitative studies (evaluation). Duplicate checklists and detailed instructions for conducting and analysing qualitative studies were excluded.

Data extraction. From each of the included publications, we extracted all criteria for assessing or reporting qualitative studies. Seventy-six items from 22 checklists were compiled into a comprehensive list. We recorded the frequency of each item across all the publications. Items most frequently included in the checklists related to sampling method, setting for data collection, method of data collection, respondent validation of findings, method of recording data, description of the derivation of themes and inclusion of supporting quotations. We grouped all items into three domains: (i) research team and reflexivity, (ii) study design and (iii) data analysis and reporting. (see Tables 2–4)

Within each domain we simplified all relevant items by removing duplicates and those that were ambiguous, too broadly defined, not specific to qualitative research, or impractical to assess. Where necessary, the remaining items were rephrased for clarity. Based upon consensus among the authors, two new items that were considered relevant for reporting qualitative research were added. The two new items were identifying the authors who conducted the interview or focus group and reporting the presence of non-participants during the interview or focus group. The COREQ checklist for explicit and comprehensive reporting of qualitative studies consists of 32 criteria, with a descriptor to supplement each item (Table 1).

COREQ: content and rationale (see Tables 1)

Domain 1: research team and reflexivity

(i) Personal characteristics: Qualitative researchers closely engage with the research process and participants and are therefore unable to completely avoid personal bias. Instead researchers should recognize and clarify for readers their identity, credentials, occupation, gender, experience and training. Subsequently this improves the credibility of the findings by giving readers the ability to assess how these factors might have influenced the researchers' observations and interpretations [13–15].

(ii) Relationship with participants: The relationship and extent of interaction between the researcher and their participants should be described as it can have an effect on the participants' responses and also on the researchers' understanding of the phenomena [16]. For example, a clinician–researcher may have a deep understanding of patients' issues but their involvement in patient care may inhibit frank discussion with patient–participants when patients believe that their responses will affect their treatment. For transparency, the investigator should identify and state their assumptions and personal interests in the research topic.

Domain 2: study design

(i) Theoretical framework: Researchers should clarify the theoretical frameworks underpinning their study so readers can understand how the researchers explored their research questions and aims. Theoretical frameworks in qualitative research include: grounded theory, to build theories from the data; ethnography, to understand the culture of groups with shared characteristics; phenomenology, to describe the meaning and significance of experiences; discourse analysis, to analyse linguistic expression; and content analysis, to systematically organize data into a structured format [10].

For peer review only

Downloaded from https://academic.oup.com/intqhc/article/19/6/349/1791966 by guest on 31 October 2021

Table 1 Consolidated criteria for reporting qualitative studies (COREQ): 32-item checklist

No	Item	Guide questions/description
**Domain 1: Research team and reflexivity**		
Personal Characteristics		
1.	Interviewer/facilitator	Which author/s conducted the interview or focus group? Page 7
2.	Credentials	What were the researcher's credentials? *E.g. PhD, MD* Page 7
3.	Occupation	What was their occupation at the time of the study? Page 7
4.	Gender	Was the researcher male or female? Page 7
5.	Experience and training	What experience or training did the researcher have? Page 7
Relationship with participants		
6.	Relationship established	Was a relationship established prior to study commencement? Page 7
7.	Participant knowledge of the interviewer	What did the participants know about the researcher? e.*g. personal goals, reasons for doing the research* Page 7
8.	Interviewer characteristics	What characteristics were reported about the interviewer/facilitator? e.g. *Bias, assumptions, reasons and interests in the research topic* Page 7
**Domain 2: study design**		
Theoretical framework		
9.	Methodological orientation and Theory	What methodological orientation was stated to underpin the study? *e.g. grounded theory, discourse analysis, ethnography, phenomenology, content analysis* Page 5
Participant selection		
10.	Sampling	How were participants selected? *e.g. purposive, convenience, consecutive, snowball* Page 6
11.	Method of approach	How were participants approached? e.*g. face-to-face, telephone, mail, email* Page 6
12.	Sample size	How many participants were in the study? Page 6
13.	Non-participation	How many people refused to participate or dropped out? Reasons? Page 7
Setting		
14.	Setting of data collection	Where was the data collected? e.*g. home, clinic, workplace* Page 6
15.	Presence of non-participants	Was anyone else present besides the participants and researchers? Page 7
16.	Description of sample	What are the important characteristics of the sample? *e.g. demographic data, date* Pages 6,7
Data collection		
17.	Interview guide	Were questions, prompts, guides provided by the authors? Was it pilot tested? Yes, see supplementary materials
18.	Repeat interviews	Were repeat interviews carried out? If yes, how many? Page 8
19.	Audio/visual recording	Did the research use audio or visual recording to collect the data? Page 7
20.	Field notes	Were field notes made during and/or after the interview or focus group? Page 7
21.	Duration	What was the duration of the interviews or focus group? Page 8
22.	Data saturation	Was data saturation discussed? Page 8
23.	Transcripts returned	Were transcripts returned to participants for comment and/or correction? Page 8
**Domain 3: analysis and findings**z		
Data analysis		
24.	Number of data coders	How many data coders coded the data? Page 8
25.	Description of the coding tree	Did authors provide a description of the coding tree? page 7
26.	Derivation of themes	Were themes identified in advance or derived from the data? Page 8
27.	Software	What software, if applicable, was used to manage the data? Page 8
28.	Participant checking	Did participants provide feedback on the findings? Page 8
Reporting		
29.	Quotations presented	Were participant quotations presented to illustrate the themes / findings? Was each quotation identified? e.*g. participant number* Pages 11 and 12
30.	Data and findings consistent	Was there consistency between the data presented and the findings? Pages 11 and 12
31.	Clarity of major themes	Were major themes clearly presented in the findings? Pages 11 and 12
32.	Clarity of minor themes	Is there a description of diverse cases or discussion of minor themes? Pages 11 and 12

(ii) Participant selection: Researchers should report how participants were selected. Usually purposive sampling is used which involves selecting participants who share particular characteristics and have the potential to provide rich, relevant and diverse data pertinent to the research question [13, 17]. Convenience sampling is less optimal because it may fail to capture important perspectives from difficult-to-reach people [16]. Rigorous attempts to recruit participants and reasons for non-participation should be stated to reduce the likelihood of making unsupported statements [18].

For peer review only

Downloaded from https://academic.oup.com/intqhc/article/19/6/349/1791966 by guest on 31 October 2021

Table 2 Items included in 22 published checklists: Research team and reflexivity domain

Item	References																					
	[26][a]	[27][a]	[6][b]	[28][b]	[32][b]	[13]	[15]	[14]	[17]	[33]	[34]	[35]	[16]	[19]	[36]	[7]	[37]	[23]	[38]	[39]	[22]	BMJ
Research team and reflexivity																						
Nature of relationship between the researcher and participants		•		•	•		•		•						•				•			
Examination of role, bias, influence	•	•			•	•	•	•							•						•	
Description of role		•		•					•	•				•	•					•	•	
Identity of the interviewer		•		•		•					•		•		•							
Continued and prolonged engagement		•				•							•	•					•	•		
Response to events	•	•				•	•	•														
Prior assumptions and experience		•						•									•			•		
Professional status		•					•								•							
Journal, record of personal experience		•								•				•								
Effects of research on researcher		•				•	•															
Qualifications		•													•							
Training of the interviewer/facilitator			•		•																	
Expertise demonstrated		•																	•			
Perception of research at inception								•						•								
Age							•															
Gender							•															
Social class							•															
Reasons for conducting study		•																				
Sufficient contact													•									
Too close to participants													•									
Empathy																	•					
Distance between researcher and participants							•															
Background								•														
Familiarity with setting																					•	

[a]Other publications, [b]Systematic review of qualitative studies; BMJ, British Medical Journal—editor's checklist for appraising qualitative research); •, item included in the checklist.

Downloaded from https://academic.oup.com/intqhc/article/19/6/349/1791966 by guest on 31 October 2021

For peer review only

Downloaded from https://academic.oup.com/intqhc/article/19/6/349/1791966 by guest on 31 October 2021

Table 3 Items included in 22 published checklists: Study design

Item	References																					
	[26][a]	[27][a]	[6][b]	[28] [b]	[32][b]	[13]	[15]	[14]	[17]	[33]	[34]	[35]	[16]	[19]	[36]	[7]	[37]	[23]	[38]	[39]	[22]	BMJ
Study design																						
Methodological orientation, ontological or epistemological basis		•		•				•	•					•					•	•	•	•
Sampling—convenience, purposive	•	•			•	•	•	•	•	•	•	•	•	•		•		•	•	•	•	•
Setting		•		•	•			•			•				•					•		
Characteristics and description of sample		•			•			•			•		•	•								
Reasons for participant selection	•	•				•		•			•											
Non-participation	•	•		•	•																	
Inclusion and exclusion, criteria		•			•	•													•			
Identity of the person responsible for recruitment				•	•						•				•							
Sample size		•		•	•						•										•	
Method of approach		•									•					•						
Description of explanation of research to participants	•				•										•							
Level and type of participation														•								
Method of data collection, e.g. focus group, in-depth interview	•	•	•	•	•	•		•	•		•	•	•	•		•				•	•	
Audio and visual recording	•	•	•	•	•	•				•	•		•						•		•	•
Transcripts			•	•	•	•			•		•		•						•			•
Setting and location	•	•		•	•		•		•		•				•						•	•
Saturation of data	•	•	•			•			•				•	•							•	
Use of a topic guide, tools, questions	•	•	•								•					•			•	•		
Field notes			•	•	•	•													•			•
Changes and modifications	•	•		•	•														•		•	
Duration of interview, focus group		•				•					•									•		
Sensitive to participant language and views		•										•		•								
Number of interviews, focus groups		•				•																
Time span																					•	
Time and resources available to the study		•																				

[a]Other publications, [b]Systematic review of qualitative studies; BMJ, British Medical Journal—editor's checklist for appraising qualitative research; •, item included in the checklist.

Table 4 Items included in 22 published checklists: Analysis and reporting

Item	References																					
	[26][a]	[27][a]	[6][b]	[28][b]	[32][b]	[13]	[15]	[14]	[17]	[33]	[34]	[35]	[16]	[19]	[36]	[7]	[37]	[23]	[38]	[39]	[22]	BMJ
Respondent validation	•	•	•		•		•		•	•			•	•			•	•	•	•		
Limitations and generalizability	•	•		•	•		•		•		•		•	•				•	•			
Triangulation	•	•		•	•	•	•	•	•					•			•		•			
Original data, quotation		•	•	•	•			•	•		•			•		•				•	•	•
Derivation of themes explicit	•	•	•	•	•		•	•			•								•			•
Contradictory, diverse, negative cases	•	•		•	•		•			•				•					•			•
Number of data analysts	•	•	•			•			•			•	•						•			•
In-depth description of analysis	•			•	•			•			•			•							•	•
Sufficient supporting data presented	•	•		•	•		•				•					•						
Data, interpretation and conclusions linked and integrated		•		•	•							•		•						•		
Retain context of data		•					•	•						•					•			
Explicit findings, presented clearly	•	•		•					•	•												
Outside checks													•	•				•	•			
Software used		•				•													•			•
Discussion both for and against the researchers' arguments	•	•		•	•																	
Development of theories, explanations		•					•			•		•										
Numerical data		•									•							•				•
Coding tree or coding system		•					•												•		•	
Inter-observer reliability		•									•										•	
Sufficient insight into meaning/perceptions of participants		•								•												
Reasons for selection of data to support findings		•			•																	
New insight		•						•														
Results interpreted in credible, innovative way									•													
Eliminate other theories													•									
Range of views														•								
Distinguish between researcher and participant voices								•														
Proportion of data taken into account														•								

[a]Other publications, [b]Systematic review of qualitative studies; BMJ, British Medical Journal—editor's checklist for appraising qualitative research, •, item included in the checklist.

For peer review only

Downloaded from https://academic.oup.com/intqhc/article/19/6/349/1791966 by guest on 31 October 2021

Researchers should report the sample size of their study to enable readers to assess the diversity of perspectives included.

(iii) Setting: Researchers should describe the context in which the data were collected because it illuminates why participants responded in a particular way. For instance, participants might be more reserved and feel disempowered talking in a hospital setting. The presence of non-participants during interviews or focus groups should be reported as this can also affect the opinions expressed by participants. For example, parent interviewees might be reluctant to talk on sensitive topics if their children are present. Participant characteristics, such as basic demographic data, should be reported so readers can consider the relevance of the findings and interpretations to their own situation. This also allows readers to assess whether perspectives from different groups were explored and compared, such as patients and health care providers [13, 19].

(iv) Data collection: The questions and prompts used in data collection should be provided to enhance the readers' understanding of the researcher's focus and to give readers the ability to assess whether participants were encouraged to openly convey their viewpoints. Researchers should also report whether repeat interviews were conducted as this can influence the rapport developed between the researcher and participants and affect the richness of data obtained. The method of recording the participants' words should be reported. Generally, audio recording and transcription more accurately reflect the participants' views than contemporaneous researcher notes, more so if participants checked their own transcript for accuracy [19–21]. Reasons for not audio recording should be provided. In addition, field notes maintain contextual details and non-verbal expressions for data analysis and interpretation [19, 22]. Duration of the interview or focus group should be reported as this affects the amount of data obtained. Researchers should also clarify whether participants were recruited until no new relevant knowledge was being obtained from new participants (data saturation) [23, 24].

Domain 3: analysis and findings

(i) Data analysis: Specifying the use of multiple coders or other methods of researcher triangulation can indicate a broader and more complex understanding of the phenomenon. The credibility of the findings can be assessed if the process of coding (selecting significant sections from participant statements), and the derivation and identification of themes are made explicit. Descriptions of coding and memoing demonstrate how the researchers perceived, examined and developed their understanding of the data [17, 19]. Researchers sometimes use software packages to assist with storage, searching and coding of qualitative data. In addition, obtaining feedback from participants on the research findings adds validity to the researcher's interpretations by ensuring that the participants' own meanings and perspectives are represented and not curtailed by the researchers' own agenda and knowledge [23].

(ii) Reporting: If supporting quotations are provided, researchers should include quotations from different participants to add transparency and trustworthiness to their findings and interpretations of the data [17]. Readers should be able to assess the consistency between the data presented and the study findings, including the both major and minor themes. Summary findings, interpretations and theories generated should be clearly presented in qualitative research publications.

Discussion

The COREQ checklist was developed to promote explicit and comprehensive reporting of qualitative studies (interviews and focus groups). The checklist consists of items specific to reporting qualitative studies and precludes generic criteria that are applicable to all types of research reports. COREQ is a comprehensive checklist that covers necessary components of study design, which should be reported. The criteria included in the checklist can help researchers to report important aspects of the research team, study methods, context of the study, findings, analysis and interpretations.

At present, we acknowledge there is no empiric basis that shows that the introduction of COREQ will improve the quality of reporting of qualitative research. However this is no different than when CONSORT, QUOROM and other reporting checklists were introduced. Subsequent research has shown that these checklists have improved the quality of reporting of study types relevant to each checklist [5, 25], and we believe that the effect of COREQ is likely to be similar. Despite differences in the objectives and methods of quantitative and qualitative methods, the underlying aim of transparency in research methods and, at the least, the theoretical possibility of the reader being able to duplicate the study methods should be the aims of both methodological approaches. There is a perception among research funding agencies, clinicians and policy makers, that qualitative research is 'second class' research. Initiatives like COREQ are designed to encourage improvement in the quality of reporting of qualitative studies, which will indirectly lead to improved conduct, and greater recognition of qualitative research as inherently equal scientific endeavor compared with quantitative research that is used to assess the quality and safety of health care. We invite readers to comment on COREQ to improve the checklist.

References

1. Moher D, Schulz KF, Altman D. The CONSORT statement: revised recommendations for improving the quality of reports of parallel-group randomized trials. *JAMA* 2001;**285**:1987–91.
2. Moher D, Cook DJ, Eastwood S *et al.* Improving the quality of reports of meta-analyses of randomised controlled trials: the QUOROM statement. Quality of Reporting of Meta-analyses. *Lancet* 1999;**354**:1896–900.

Downloaded from https://academic.oup.com/intqhc/article/19/6/349/1791966 by guest on 31 October 2021

For peer review only

3. STROBE Statement: Strengthening the reporting of observational studies in epidemiology. http://www.strobe-statement.org/Checkliste.html
4. Stroup DF, Berlin JA, Morton SC *et al.* Meta-analysis of observational studies in epidemiology: a proposal for reporting. Meta-analysis Of Observational Studies in Epidemiology (MOOSE) group. *JAMA* 2000;**283**:2008–12.
5. Moher D, Jones A, Lepage L. Use of the CONSORT Statement and quality of reports of randomized trials. A comparative before-and-after evaluation. *JAMA* 2001;**285**:1992–5.
6. Mills E, Jadad AR, Ross C *et al.* Systematic review of qualitative studies exploring parental beliefs and attitudes toward childhood vaccination identified common barriers to vaccination. *J Clin Epidemiol* 2005;**58**:1081–8.
7. Knafl KA, Howard NJ. Interpreting and reporting qualitative research. *Res Nurs Health* 1984;**7**:7–14.
8. Sofaer S. Qualitative research methods. *Int J Qual Health Care* 2002;**14**:329–36.
9. Wright EB, Holcombe C, Salmon P. Doctor's communication of trust, care, and respect in breast cancer: qualitative study. *BMJ* 2004;**328**:864–8.
10. Liamputtong P, Ezzy D. *Qualitative Research Methods*. 2nd edn. Melbourne, Victoria: Oxford University Press, 2005.
11. Krueger RA, Casey MA. *Focus Groups. A Practical Guide for Applied Research*. Thousand Oaks CA: Sage Publications, 2000.
12. Morgan DL. *Focus Groups as Qualitative Research*. Newbury Park, California: Sage, 1988.
13. Giacomini MK, Cook DJ. Users' guides to the medical literature XXIII. Qualitative research in health care. A. Are the results of the study valid? *JAMA* 2000;**284**:357–62.
14. Malterud K. Qualitative research:standards challenges guidelines. *Lancet* 2001;**358**:483–8.
15. Mays N, Pope C. Qualitative research in health care: assessing quality in qualitative research. *BMJ* 2000;**320**:50–2.
16. Elder NC, William L. Reading and evaluating qualitative research studies. *J Fam Pract* 1995;**41**:279–85.
17. Cote L, Turgeon J. Appraising qualitative research articles in medicine and medical education. *Med Teach* 2005;**27**:71–5.
18. Altheide D, Johnson J. Criteria for assessing interpretive validity in qualitative research. In Denzin N, Lincoln Y (eds). *Handbook of Qualitative Research*. Thousand Oaks CA: Sage Publications, 1994.
19. Fossey E, Harvey C, McDermott F *et al.* Understanding and evaluating qualitative research. *Aust N Z J Psychiatry* 2002;**36**:717–32.
20. Seale C, Silverman S. Ensuring rigour in qualitative research. *Eur J Public Health* 1997;**7**:379–84.
21. Scheff T. Single case analysis in the health sciences. *Eur J Public Health* 1995;**5**:72–4.
22. Bluff R. Evaluating qualitative research. *Br J Midwifery* 1997;**5**:232–5.
23. Popay J, Rogers A, Williams G. Rationale and standards for the systematic review of qualitative literature in health services research. *Qual Health Res* 1998;**8**:341–51.
24. Blumer H. *Critiques of Research, in the Social Sciences*. New Brunswick, NJ: Transaction Books, 1979.
25. Delaney A, Bagshaw SM, Ferland A *et al.* A systematic evaluation of the quality of meta-anlyses in the critical care literature. *Crit Care* 2005;**9**:575–82.
26. Critical Skills Appraisal Programme (CASP) 10 Questions to help you make sense of qualitative research: Milton Keynes Primary Care Trust, 2002.
27. Spencer L, Ritchie J, Lewis J *et al. Quality in Qualitative Evaluation: A Framework for Assessing Research Evidence*. London: Cabinet Office. Government Chief Social Researcher's Office, 2003.
28. Campbell R, Pound P, Pope C *et al.* Evaluating meta-ethnography: a synthesis of qualitative research on lay experience of diabetes and diabetes care. *Soc Sci Med* 2003;**56**:671–84.
29. Feder GS, Hutson M, Ramsay I *et al.* Women exposed to intimate partner violence: expectations and experiences when they encounter health care professionals: a meta-analysis of qualitative studies. *Arch Intern Med* 2006;**166**:22–37.
30. Pound P, Britten N, Morgan M *et al.* Resisting medicines: a synthesis of qualitative studies of medicine taking. *Soc Sci Med* 2005;**61**:133–55.
31. Smith LK, Pope C, Botha JL. Patients' help-seeking experiences and delay in cancer presentation: a qualitative synthesis. *Lancet* 2005;**366**:825–31.
32. Walter FM, Emery J, Braithwaite D *et al.* Lay understanding of familial risk of common chronic diseases: a systematic review and synthesis of qualitative research. *Ann Fam Med* 2004; **2**:583–94.
33. Inui TS, Frankel RM. Evaluating the quality of qualitative research: a proposal pro-term. *J Gen Intern Med* 1991;**6**:485–6.
34. Boulton M, Fitzpatrick R, Swinburn C. Qualitative research in health care: II A structured review and evaluation of studies. *J Eval Clin Pract* 1996;**2**:171–9.
35. Dixon-Woods M, Shaw RL, Agarwal S *et al.* The problem of appraising qualitative research. *Qual Saf Health Care* 2004;**13**:223–5.
36. Hoddinott P, Pill R. A review of recently published qualitative research in general practice. More methodological questions than answers? *Fam Pract* 1997;**14**:313–9.
37. Kuzel AJ, Engel JD, Addison RB *et al.* Desirable features of qualitative research. *Fam Pract Res J* 1994;**14**:369–78.
38. Treloar C, Champness S, Simpson PL *et al.* Critical appraisal checklist for qualitative research studies. *Indian J Pediatr* 2000;**67**:347–51.
39. Cesario S, Morin K, Santa-Donato A. Evaluating the level of evidence in qualitative research. *J Obstet Gynecol Neonatal Nurs* 2001;**31**:708–14.

Accepted for publication 7 July 2007

For peer review only

Downloaded from https://academic.oup.com/intqhc/article/19/6/349/1791966 by guest on 31 October 2021

BMJ Open

Care seeking for under-five children and vaccine perceptions during the first two waves of the COVID-19 pandemic in Lagos State, Nigeria: a qualitative exploratory study

Journal:	*BMJ Open*
Manuscript ID	bmjopen-2022-069294.R2
Article Type:	Original research
Date Submitted by the Author:	18-Feb-2023

Complete List of Authors:	Bakare, Ayobami; Karolinska Institutet, Department of Global Public Health; University College Hospital, Department of Community Medicine<br>Olojede, Omotayo; University College Hospital Ibadan, Department of Paediatrics<br>King, Carina; Karolinska Institute<br>Graham, Hamish; Centre for International Child Health, University of Melbourne, MCRI, Royal Children's Hospital; University College Hospital Ibadan, Department of Paediatrics<br>Uchendu, Obioma; University College Hospital Ibadan, Department of Community Medicine; University of Ibadan College of Medicine, Department of Community Medicine<br>Colbourn, Timothy; UCL Institute for Global Health<br>Falade, Adegoke; University College Hospital Ibadan, Department of Paediatrics; University of Ibadan College of Medicine, Department of Paediatrics<br>Alvesson, Helle; Karolinska Universitetssjukhuset, Global Public Health

**Primary Subject Heading**:	Public health
Secondary Subject Heading:	Qualitative research
Keywords:	COVID-19, Community child health < PAEDIATRICS, PREVENTIVE MEDICINE, PRIMARY CARE, PUBLIC HEALTH

SCHOLARONE™ Manuscripts

I, the Submitting Author has the right to grant and does grant on behalf of all authors of the Work (as defined in the below author licence), an exclusive licence and/or a non-exclusive licence for contributions from authors who are: i) UK Crown employees; ii) where BMJ has agreed a CC-BY licence shall apply, and/or iii) in accordance with the terms applicable for US Federal Government officers or employees acting as part of their official duties; on a worldwide, perpetual, irrevocable, royalty-free basis to BMJ Publishing Group Ltd ("BMJ") its licensees and where the relevant Journal is co-owned by BMJ to the co-owners of the Journal, to publish the Work in this journal and any other BMJ products and to exploit all rights, as set out in our licence.

The Submitting Author accepts and understands that any supply made under these terms is made by BMJ to the Submitting Author unless you are acting as an employee on behalf of your employer or a postgraduate student of an affiliated institution which is paying any applicable article publishing charge ("APC") for Open Access articles. Where the Submitting Author wishes to make the Work available on an Open Access basis (and intends to pay the relevant APC), the terms of reuse of such Open Access shall be governed by a Creative Commons licence – details of these licences and which Creative Commons licence will apply to this Work are set out in our licence referred to above.

Other than as permitted in any relevant BMJ Author's Self Archiving Policies, I confirm this Work has not been accepted for publication elsewhere, is not being considered for publication elsewhere and does not duplicate material already published. I confirm all authors consent to publication of this Work and authorise the granting of this licence.

For peer review only

Care seeking for under-five children and vaccine perceptions during the first two waves of the COVID-19 pandemic in Lagos State, Nigeria: a qualitative exploratory study

Ayobami A Bakare[1,2]*, Omotayo E Olojede[3], Carina King[2], Hamish R Graham[4], Obioma Uchendu[1,5], Tim Colbourn[6], Adegoke G Falade[3,7], Helle Mölsted Alvesson[2] on behalf of the INSPIRING Project Consortium**

Affiliations

[1] Department of Community Medicine, University College Hospital, Ibadan, Nigeria.

[2] Department of Global Public Health, Karolinska Institutet, Stockholm, Sweden.

[3] Department of Paediatrics, University College Hospital, Ibadan, Nigeria.

[4] Centre for International Child Health, Murdoch Children's Research Institute, University of Melbourne, Royal Children's Hospital, Parkville, Victoria, Australia.

[5] Department of Community Medicine, College of Medicine, University of Ibadan, Ibadan, Nigeria.

[6] Institute for Global Health, University College London, London, UK.

[7] Department of Paediatrics, College of Medicine, University of Ibadan, Ibadan, Nigeria.

***Correspondence to:**
Ayobami A Bakare
Department of Global Public Health, Karolinska Institutet, Stockholm, Sweden
bakare.ayobami.adebayo@ki.se

****Full INSPIRING consortium:** Carina King (Karolinska Institutet); Tim Colbourn, Rochelle Ann Burgess, Agnese Iuliano (UCL); Hamish Graham (Melbourne); Eric McCollum (Johns Hopkins); Tahlil Ahmed, Samy Ahmar, Christine Cassar, Paula Valentine (Save the Children UK); Adamu Isah, Adams Osebi, Magama Abdullahi, Ibrahim Haruna (Save the Children Nigeria); Temitayo Folorunso Olowookere (GSK Nigeria); Matt MacCalla (GSK UK); Adegoke Gbadegesin Falade, Ayobami Adebayo Bakare, Obioma Uchendu, Julius Salako, Funmilayo Shittu and Damola Bakare, Omotayo Olojede (University of Ibadan).

Keywords: COVID-19, lockdown, under-five children, care-seeking, Nigeria

Word count: 4703 words (excluding declarations, references and tables)

For peer review only - http://bmjopen.bmj.com/site/about/guidelines.xhtml

ABSTRACT

Objective

To explore health care-seeking practices for children and the context-specific direct and indirect effects of public health interventions during the first two waves of COVID-19 in Lagos State, Nigeria. We also explored decision making around vaccine acceptance at the start of COVID-19 vaccine roll-out in Nigeria

Design, setting and participants

A qualitative explorative study involving 19 semi-structured interviews with healthcare providers from public and private primary health facilities and 32 interviews with caregivers of under-five children in Lagos, from December 2020 to March 2021. Participants were purposively selected from healthcare facilities to include community health workers, nurses, and doctors, and interviews were conducted in quiet locations at facilities. A data-driven reflexive thematic analysis according to Braun & Clark 2019 was conducted.

Findings

Two themes were developed: appropriating COVID-19 in belief systems, and ambiguity about COVID-19 preventive measures. The interpretation of COVID-19 disease ranged from fearful to considering it as a 'scam' or 'falsification from the government'. Underlying distrust in government fueled COVID-19 misperceptions. Care seeking for children under-five was affected, as facilities were seen as contagious places for COVID-19. Caregivers resorted to alternative care and self-management of childhood illnesses. COVID-19 vaccine hesitancy was a major concern among healthcare providers compared to community members at the time of vaccine roll-out in Lagos, Nigeria. Indirect impacts of COVID-19 lockdown included diminished household income, worsening food insecurity, mental health challenges for caregivers and reduced clinic visits for immunization.

Conclusion

The first wave of the COVID-19 pandemic in Lagos was associated with reductions in care seeking for children, clinic attendance for childhood immunizations, and household income. Strengthening health and social support systems with context-specific interventions and correcting misinformation is crucial to building adaptive capacity for response to future pandemics.

For peer review only

Strengths and limitations of this study

- A key strength of this study was the inclusion of perspectives from both caregivers and healthcare providers in private and public health facilities, and the recruitment of various cadres of healthcare providers.
- The use of semi-structured interviews, conducted while the pandemic was on-going, provided the opportunity to understand individual perspectives and experiences.
- Perspectives captured in this study may have missed some negative impacts of COVID-19 on care seeking given caregivers were recruited from health facilities and may therefore differ from the wider community.
- Findings from this study may not reflect all aspects considered important to the participants as communities and healthcare workers were not consulted in the design of the interview guides.

For peer review only

For peer review only - http://bmjopen.bmj.com/site/about/guidelines.xhtml

INTRODUCTION

The COVID-19 pandemic was declared a public health emergency of international concern in January 2020 by the World Health Organization.[1] Differential negative impacts have been reported across the globe due to the COVID-19 pandemic. While some countries have reported a high number of deaths due to COVID-19, others particularly in sub-Sahara Africa have reported low mortality,[2] but have suffered significant social and economic impacts with recovery, likely to take a protracted course.[3] As of March 27, 2022, over eight million cases and 170,000 deaths had been reported in Africa, although estimates of actual cases (505.6 million) and deaths (439,500) in the region are much higher.[4,5] Within Africa, Nigeria reported the fourth highest number of COVID-19 cases in 2020-2021, with 215,164 reported cases (3.4% of the African total) and 92 million estimated cases.[6]Lagos State was the epicentre of the COVID-19 pandemic in Nigeria during this period, accounting for more than 30% of Nigeria's reported cases, with the first cases identified in late February 2020.[7,8]

The pandemic has been a major stressor to health systems, exposing and exacerbating pre-existing fragility and inequities within the system. [9,10] Given the absence of effective and widely available COVID-19 treatments during the first and second waves—February-October 2020 and November 2020-April 2021 respectively, [11] containment measures were based on public health measures like movement and travel restrictions (i.e. 'lockdowns'), physical distancing, personal hygiene and use of personal protective equipment (PPE).[12] Negative impacts of these containment measures on social life and mental well-being, education, economy, health service delivery and utilization have been reported, but mostly from non-empirical data and outside the African context.[13–18] Early predictions of Africa being worst hit by the COVID-19 pandemic did not come to fruition,[19] underscoring the need for context-specific empirical data. While the direct clinical impact of COVID-19 has affected adults more directly in this period, children are not exempt from indirect effects of mitigations, although observed data from Africa is lacking.[20,21]

In March 2020, the Nigerian government imposed several public health measures. The initial COVID-19 pandemic wave in Nigeria was characterized by fear, confusion and instability in the existing social structures, with misinformation fueled by social media reports and lockdown measures imposed by the government.[7,22–25] These may have had knock-on effects on healthcare service utilization and delivery. While multiple studies, largely from high-income contexts, have reported reductions in child illnesses and hospital admissions during periods of COVID-19 restrictions, fewer have explored the role of changes in care-seeking behaviour for children during this period and their implications for future public health responses to disease outbreaks.[16,26]

In Nigeria, under-five mortality remains high, and is not on-track to meet the 2030 Sustainable Development Goal global target of having less than 25 deaths per 1000 live births.[27] Pneumonia, malaria and diarrhoea are leading causes of under-five deaths in the country, responsible for almost 40% of under-five deaths in 2018.[28] Nigeria also experiences multiple outbreaks of diseases of public health significance annually, including meningococcal disease, Yellow fever, and Lassa fever. [29] Given the existing burden of pneumonia, malaria, and diarrhoea among children, the magnitude of the COVID-19 pandemic and response, and the frequency of disease outbreaks requiring public health response which may require mass vaccination, it is important to understand how the COVID-19 pandemic affected care-seeking for under-five children as well as decision making around vaccine introduction for outbreak control. We therefore aimed to understand care-seeking practices for young children and the context-specific direct and indirect effects of public health interventions during the first two waves of COVID-19 pandemic and decision making around vaccine acceptance at the start of COVID-19 vaccine roll-out in Lagos State, Nigeria.

For peer review only

For peer review only - http://bmjopen.bmj.com/site/about/guidelines.xhtml

METHODS

Study design

This was an exploratory qualitative study using reflexive thematic analysis according to Braun & Clark.[30] We conducted semi-structured interviews with caregivers of children under-five and healthcare providers to gather perspectives on care-seeking practices during the first two waves of the COVID-19 pandemic in Lagos State, Nigeria (February-October 2020 and November 2020-April 2021). The study was conducted as part of the process evaluation of the Lagos INSPIRING project, which is evaluating a child pneumonia health system intervention (study registration: ACTRN12621001071819). We followed the Consolidated Criteria for Reporting Qualitative Research (COREQ) guidelines for reporting.[31]

Setting

The study was conducted in Ikorodu Local Government Area (LGA) in Lagos State. Lagos is the most populous state in Nigeria with an estimated population of 24.6 million people in 2022, [32] and is an economic hub in West Africa. Ikorodu is one of five administrative divisions of Lagos. It is a peri-urban area, with fishing as the predominant economic activity in the rural parts of the LGA, and small and medium scale entrepreneurship as the major economic activity in the urban parts of the LGA. The LGA is served by two government-owned secondary health facilities (General Hospitals), 28 primary healthcare centers (PHCs) and over one hundred private facilities. Of the 28 PHCs, seven are designated as 'flagship' facilities by the Lagos State government, as they have more personnel and equipment and run 24-hour services for children and adults. There is at least one flagship PHC in each of Ikorodu's six Local Council Development Areas (LCDAs) and all of them remained open during the first two waves of the pandemic. The flagship PHCs also acted as COVID-19 vaccination centres, except one facility which did not have a medical doctor.

As part of the public health measures, Lagos was placed on lockdown by the Federal Government of Nigeria on the 30th March 2020.[7] The lockdown lasted 35 days and included a ban on social and economic activities, restriction of all non-essential movements, suspension of commuter services, closure of schools and retail shops and prohibition of mass gatherings except for funeral services.[33] Unlike PHCs and private health facilities, service provisions were limited to emergency cases in the public secondary-level facilities. A gradual easing of the lockdown commenced from the 4th May 2020 with no re-instatement of movement restrictions during the second wave (see Appendix I).[7] In addition, there was a period of civil unrest in Lagos, including Ikorodu LGA (the 'EndSARS' protests against police brutality [34]), between 8th and 22nd October 2020, when a curfew was imposed.

Participants and sampling

We purposively selected healthcare providers who attended to sick children from the seven flagship PHCs and six nearby private facilities (Table 1). To ensure representation of each cadre of healthcare provider, the categories of staff targeted for recruitment (nurse, community health workers, and doctors) was adapted to each facility. We recruited caregivers of children under-five years presenting at the outpatient departments (i.e. with an illness) or immunization clinics (i.e. healthy children) of seven flagship PHCs and one secondary hospital. Caregivers were recruited by female clinical project staff, who screened every child brought to outpatient departments of the facilities for pneumonia. In each facility, we used convenience sampling to recruit four caregivers of under-five children at random (n=32): two caregivers of an acutely unwell child (from outpatients) and two caregivers of a child with no current illness episode (from the immunization clinic). This sample size was based on practical considerations of the time needed to recruit participants and the expectation that it would be sufficient numbers to achieve saturation. All participants approached for the study agreed to take part.

For peer review only - http://bmjopen.bmj.com/site/about/guidelines.xhtml

Table 1. Summary of participants' characteristics

Caregivers n=32		Healthcare providers n=19	
**Gender**		**Gender**	
Male	0 (0.0)	Male	5 (26.0)
Female	32 (100.0)	Female	14 (74.0)
Mean age (±SD)	31±5.0 years	Mean age (±SD)	38±8.1 years
**Median no of children (range)**	2 (1-5)	**Median year of experience**	11 (2-40)
**Educational level**		**Educational level**	
Primary	2 (6.3)	Diploma	9 (47.4)
Secondary	13 (40.6)	Tertiary	9 (47.4)
Tertiary	17 (53.1)	Postgraduate	1 (5.2)
**Religion**		**Religion**	
Christianity	25 (78.1)	Christianity	15 (78.9)
Islam	7 (21.9)	Islam	4 (21.1)
**Occupation/Cadre**		**Occupation/Cadre**	
Self-employed	21 (64.5)	Doctor	7 (36.8)
Employed	5 (16.1)	Nurse	6 (31.6)
No employment	6 (19.4)	CHEW	6 (31.6)

CHEW: Community Health Extension Worker.

Data collection

Interviews were conducted from 10 December 2020 to 18 March 2021. The semi-structured interview guides were based on the literature on care-seeking practices and knowledge about COVID-19 during the INSPIRING project formative phase and revised to capture the emerging COVID-19 vaccine programme roll-out in Nigeria. The interview guide for caregiver interviews had three sections, focused on: participants' family and socio-demographic information, their experiences of 2020 in light of COVID-19 including their perception of the illness and economic impacts, and care-seeking practices for children under-five years. The interview guide for healthcare provider interviews had three sections focusing on: service provision, facility adaptation to the COVID-19 pandemic, and care seeking for sick under-five children (Appendices II-IV).

The research team was comprises of pediatricians, social science and public health specialists. The interviews were conducted by OEO, a male Master's student from Nigeria with experience of the local context, with support from the female clinical study staff who recruited participants based at each facility.. Interviews were conducted in English or Yoruba (the indigenous local language in Ikorodu LGA), depending on the participant preference. The interviewer lived in Ikorodu before and during the COVID-19 pandemic and had previously visited the participating health facilities for other data collection activities. [35] Caregivers' interviews were conducted at the health facility or in another convenient place agreed by the participants. Providers' interviews were held at the facility. Each interview lasted between

For peer review only - http://bmjopen.bmj.com/site/about/guidelines.xhtml

30-40 minutes and no repeat interviews were carried out. All interviews were voice-recorded, transcribed and translated into English, before being stored in a secure cloud platform with access granted to only research team members. No transcripts were returned to the participants for review.

Data analysis

After cross-checking of the transcripts, the analysis team (AAB, OEO, HMA and CK) conducted a data-driven thematic analysis to develop themes and subthemes.[36] AAB and OEO independently reviewed all the transcripts to identify initial codes which were reconciled in NVivo.[37] Healthcare provider and caregiver interviews were initially coded separately, and then reviewed by the analysis team to identify common themes and sub-themes, which were refined in subsequent analysis meetings. The process continued till the patterns of meaning were clear. The unit of analysis was COVID-19 related responses in the interviews.

Patient and public involvement

The overarching study was designed through a co-design workshop involving representatives from the Nigerian governments, community-based organizations, professionals, Save the Children and evaluation partners. However, patients were not involved in the design of this study. Findings from this study were not discussed with the participants, but will be incorporated into the final report that will be disseminated to the relevant stakeholders including healthcare providers and community-based organizations.[38]

FINDINGS

We identified two overarching themes which were common to caregivers and healthcare workers: appropriating COVID-19 in the belief systems, and ambiguity towards preventive measures (Table 2). When the findings differ between healthcare providers and caregivers, this is specifically noted in the text.

Table 2. Summary of themes and sub-themes

Organizing themes	Themes	Sub-themes
Appropriating COVID-19 in the belief systems	Political placement of COVID-19	Disbelief in the virus' existence
		Misinformation and misconceptions about COVID-19
	Socio-theological placement of COVID-19	Religious explanation for COVID-19
		Social placement of COVID-19
	Medical placement of COVID-19	COVID-19 infection is real
		Healthcare as a source of infection

For peer review only - http://bmjopen.bmj.com/site/about/guidelines.xhtml

**Organizing themes**	**Themes**	**Sub-themes**
Ambiguity about COVID-19 preventive measures	Unappealing lockdown experiences and associated adaptive mechanisms	Direct impact of lockdown
		Indirect impact of lockdown
		Health system adaption and its consequences
	Drivers of COVID-19 vaccine hesitancy	Misinformation and conspiracy theories about COVID-19 vaccine
		Fear and worries about COVID-19 vaccines
		Distrust in government efforts regarding COVID-19 vaccines
		Media influence on COVID-19
	Drivers of COVID-19 vaccine uptake	Motivation to accept COVID-19 vaccine among healthcare providers
		Motivation to accept COVID-19 among community members or caregivers

For peer review only

For peer review only - http://bmjopen.bmj.com/site/about/guidelines.xhtml

Appropriating COVID-19 in belief systems

This first theme elucidates plurality in the placement of COVID-19 within the context of existing belief systems. Caregivers and healthcare providers ascribed various causes to the emergence of COVID-19 including political, religious, social and geographical dimensions.

From the healthcare providers interviews, social and political placements of COVID-19 emergence were commonly reported. To some healthcare providers, COVID-19 was not perceived as a public health problem in Nigeria.

"Except that they would say that I am a medical practitioner but I still have the impression that there is no COVID in Nigeria. Don't mind me, it's just my own belief. (Doctor—male, public facility)

The COVID-19 pandemic was framed through a political lens, with distrust in the government shaping disbelief in the disease. This distrust in government provided an opening for misinformation about the virus and control measures with participants describing COVID-19 as "a lie" and "a deceit from the government". The distrust also fed into caregivers' perceptions about COVID-19 surveillance, with some caregivers reportedly delaying care seeking to avoid being automatically added to the COVID-19 daily government case list. The disbelief of the existence of COVID-19 had social associations with participants believing that the disease would not affect 'the poor' or 'black man'.

"There were some people that were like nothing is happening, we've not seen someone with it here, none of our relatives had it so it's just a scam. They don't believe it, most people don't believe it". (CHEW—female, public facility)

To some caregivers, COVID-19 was symbolic and they offered religious explanations, describing it as a test of faith, signs of the 'end of time', a "punishment from God" or the "work of the devil", but this was not apparent among healthcare providers

"It's just like God wanted to deliberately punish people for their bad behaviours […]. Before, when one is sick, they'll say they should carry the individual, if it's our governors, they'll take flight and fly them out of the country. But when COVID-19 came, no one can come inside or go outside. Everyone is static (immobile in lockdown), so it's not COVID-19 again. It's God's judgement on us." (Mother—sick child, 1 child)

Other participants believed that COVID-19 existed as a symptomatic disease caused by a medical germ. Healthcare facilities were described as "contagious" - a source of infection, and hospital avoidance during the acute phase of the pandemic was reported by both caregivers and healthcare providers. Given health facilities were considered high-risk places, this perception resulted in (i) no care-seeking practices for some sick under-five children as caregivers resorted to self-treatment of their child's illness by seeking care from drug sellers instead, and (ii) delayed presentation at health facilities when the child's condition had worsened. Similarly, when caregivers identified COVID-19 signs in their child they avoided hospital for fear of COVID-19 diagnosis or referral to isolation.

They didn't come. A lot of people were practicing self-medication. People who had cough for example, they didn't come for treatment for fear of being told they had COVID. They kept managing it at home. (CHEW—female, public facility)

"Like one of my neighbours when her baby was running a temperature, she could not bring the baby to the hospital because she said when she goes to the hospital - now they will say her baby have this thing high fever, they should take him to isolation center. Because of that she now went to the pharmacy and brought some (medicine)" (Mother—healthy child, 3 children)

Both caregivers and healthcare workers reported being extra-careful in hospital settings, and sometimes this led to inaccessibility of care if healthcare providers suspected COVID-19 or had inadequate protective equipment. In contrast, one healthcare provider noted that service delivery for children did not change, stating that COVID-19 infections in children are not as severe as that of adults, and it would be unethical to deny children access to healthcare.

For peer review only - http://bmjopen.bmj.com/site/about/guidelines.xhtml

Ambiguity about COVID-19 preventive measures

This theme details various responses, experiences, and effects of recommended COVID-19 preventive measures and associated adaptations.

The lockdown was perceived as an unpleasant and difficult period as participants were restricted to indoor livelihoods with little or no access to transportation. Caregivers reported indirect effects of lockdown that could affect care-seeking, including diminished household incomes which necessitated loan acquisition or seeking help from family members. Household food insecurity was exacerbated, and caregivers reported reducing their consumption to save food for their children. There was avoidance of social functions, mental health challenges and a focus on basic needs:

"l have two teachers in my compound, not government teachers but private teachers. When the lockdown started then, the man is a teacher in private school, the woman is a teacher in a private school. As the school was not open, no salary, no money, nothing, nothing. For them to feed was problem, [never mind] if the baby falls sick, and now there is no money to take the baby to hospital. Sometimes, they will go and do herbal, this thing agbo (herbal concoction)" (Mother—healthy child, 3 children)

Health facilities made adjustments to ensure continuous service delivery without undermining safety. Face-masking, physical distancing, and improved personal hygiene were adopted; however, they created additional problems such as discomfort (face-masking), denied access to care, or seeking medical advice from people without medical training. Caregivers complied with the rule although there were reports of anger and verbal assaults on healthcare providers when these measures were enforced at the health facilities.

There was a continuation of routine vaccination services during the lockdown, but caregivers' incorrect assumption of PHC closures during the lockdown (secondary facilities were closed to non-emergency cases), compliance with the lockdown order and fear of COVID-19 partly contributed to reduced attendance at the immunization clinic as reported by a CHEW:

"If you remember even on social media (mass media), it was broadcasted that if what you want to do at the hospital is not very important, stay indoors and stay safe. So people adhered to that rule, to the extent that when we went for outreach services, we asked them why they haven't been coming for immunization. Then they will say it's because of the lockdown, and then "corona" stopped us from coming out. They would also claim they don't know that the facility still runs its services" (CHEW—female, public facility)

When COVID-19 vaccines became available in Nigeria, there were mixed perceptions and ambiguity towards them. Among some caregivers, the vaccine was regarded as "a mark of the beast", or a depopulation strategy from Western countries. Religious belief, misinformation and fear of side effects were reasons identified by caregivers for COVID-19 vaccine hesitancy. Healthcare providers, in contrast, expressed distrust in the government and were concerned about vaccine safety, quality, short timeline for vaccine development and the government's aggression towards COVID-19. They believed the vaccines were not tested very well in Nigeria before being approved.

"*That thing (COVID-19 vaccine) is not well tested that's my point. It's supposed to go through a series of tests before allowing it to come into this country. So I cannot even advise anyone to take it.*" (Nurse—female, private hospital)

Social media (WhatsApp, Facebook, Instagram) was identified as a source of misinformation about the vaccine. One healthcare provider queried the decision of the government to accept donated vaccines that are being rejected by other countries, as reported on social media. Similarly, vaccines sent to Nigeria were presumed to be of sub-optimal quality compared to the ones used abroad but this was linked to distrust in governments.

"Some people (healthcare providers) don't want to take it because of the things we have seen on social media that if you take it, it can cause this and that" (CHEW—female, public facility)

For peer review only - http://bmjopen.bmj.com/site/about/guidelines.xhtml

However, some healthcare providers and caregivers had positive perceptions of the vaccine, describing it as beneficial to the recipients, such as preventing sudden death and protecting against the virus. Others also showed trust in the government believing that the government cannot bring vaccines if they are harmful. Some caregivers also expressed willingness to receive the vaccine given that they are utilizing an existing routine immunization programme.

"If the vaccine comes, we know there's a reason why the government brought it. It has a work it wants to accomplish, which is why they want to bring it; we will take it" (Mother—sick child, 4 children)

Perceived higher risk of infection, the possibility of vaccines becoming scarce, a sense of responsibility to clients, motivation from senior colleagues or health managers, and later positive testimonies from recipients, were identified as drivers of uptake among healthcare providers. Being a requirement for overseas travel or pilgrimage, counselling, and public awareness were reported by healthcare providers as drivers of vaccine uptake among community members. Few healthcare providers who had taken the vaccine identified self-reflection and personal inquiry as ways they dealt with the misinformation about the vaccine.

"I heard they were cloning the vaccine in some European countries. That was my fear but when I did my own research. I found out that there is no issue." (Doctor—female, public facility)

Despite the fear and negative perceptions, community members turned out en masse to receive the vaccine, and turnout exceeded expectations, making the supply inadequate.

*We were even surprised. I wasn't expecting people to come out. It was supposed to be a 10 day program […]but we extended further for four weeks or thereabout. People were still coming, we had to tell them that there was no more vaccination. (*Doctor—male, public facility).

DISCUSSION

It is important to understand both community and healthcare workers' perceptions and experiences during the initial COVID-19 waves to adapt the provision of health care services to children during future pandemics. In the Nigerian context, participants reported both direct and indirect effects on care seeking for children, especially during the acute lockdown periods. Both groups of participants interpreted the COVID-19 pandemic through medical, political, social and economic lenses; however religious interpretation of the pandemic was more prominent among caregivers. Care seeking for children under-five was affected in part due to the perception of healthcare settings being contagious, fear of COVID-19 diagnosis, and limited access to transportation. Adapting to seek care from alternative sources for sick children was reported by both groups. COVID-19 vaccine hesitancy was a major issue among healthcare providers, but less so among community members at the time of vaccine roll-out in Lagos. The motivations for vaccine uptake differed between the groups, and social media seemed to play a crucial role in shaping acceptability of the COVID-19 vaccine.

Our study suggests that COVID-19 related misinformation, rooted in a general distrust of government and cutting across every aspect of the COVID-19 response (including vaccine roll-out), had negative influences on care-seeking for children. This resonates with findings elsewhere in Africa and globally that misinformation and misleading interpretations of health information (e.g. daily reporting of cases and deaths from COVID-19 and fear of being counted as a COVID-19 case, assumption of facility closure during the lockdown) contributed to hospital avoidance,[16,39,40] and therefore requires consideration and active management in future outbreaks. [41] Conversely, the diversity in COVID-19 placement could conceivably have positive influences on care seeking. For instance, religious beliefs relating to COVID-19 may provide emotional resilience and motivate caregivers to do everything possible to protect their children.[42] Fear of COVID-19 may similarly motivate caregivers to seek care early and get vaccinated, and even a disbelief in COVID-19 may motivate caregivers to go about business as usual.

For peer review only - http://bmjopen.bmj.com/site/about/guidelines.xhtml

While there were people who did not believe in COVID-19 and/or did not seek care to avoid being caught up in the response (e.g. wanting to avoid isolation centres), some took it seriously and many integrated religious interpretations into their understanding of the disease. A study conducted in Nigeria found that religion and religious institutions, focused on Christianity, could have a negative influence on illness perception and behaviour, but that most Nigerian Christians comfortably integrated religious and physical health domains.[43]Additionally, some religious organizations actively encouraged adherence to COVID-19 preventive measures.[43] These findings highlight the dynamic process of classifying new diseases, as seen in the emergence of Ebola disease,[44] and the need for socio-cultural considerations and community participation in public health planning and communication, as well as active feedback and management of rumours and misinformation during the response.[45,46]

When caregivers decided to seek care for their children, lack of transportation due to lockdown inhibited access. Our finding agrees with an online survey conducted in Nigeria,[47] but contrasts with a study conducted in the Netherlands which reported parental non-deterrence in care seeking for a sick child. [48] Though the nature of illness could have been responsible for this contrasting finding, given the different epidemiological profiles, differences in health systems, COVID-19 related public health measures, as well as better health literacy around COVID-19, also have modulating effects. As reported in the UK, positive experiences from the National Health Service and support from others were positive influencers of care seeking, whereas fear driven by media and community were barriers to parental care seeking. [49] Worsened household income and food security reported during the acute phase of COVID-19 are in keeping with findings in other African countries, and these have the potential to exacerbate child malnutrition and mortality. [50,51] Like in other settings,[52–55] we found evidence suggesting decreased childhood immunization during the lockdown but the extent is unclear as healthcare providers reported using outreach services to vaccinate defaulters.

Healthcare services being considered as high-risk settings for infection influenced care seeking practices for children. Similar to reports in Nigeria and elsewhere, caregivers were avoiding hospitals for fear of contracting COVID-19. [49,56–58] The resultant self-management of childhood illness and decreased healthcare service utilization are in keeping with other studies from Europe and Africa. [57–60] Studies within and outside Nigeria have also reported increased self-medication practice for the prevention and treatment of COVID-19 related symptoms but did not focus on self-medication for children during the pandemic.[61–63] A study conducted in Uganda also found higher neonatal mortality and morbidity during the lockdown.[64] Estimating the impacts of reduced hospital visits, seeking care from alternative sources, delayed hospital visits and increased self-medication for sick children was outside the scope of this study but will be crucial for understanding the indirect effects of COVID-19 public health measures. Nevertheless, our study supports the need for intelligent health communication and flexible approaches to increasing service delivery capacity, such as mobile outreach clinics to maintain health care access for children. [20,65] A study conducted in the UK hypothesized that decreased incidence of childhood illness during the lockdown period contributed to low paediatric admission for common and severe childhood illness during the lockdown; [66] however, hospital avoidance, care seeking from alternative sources and delayed presentation should not be dismissed.

The underlying distrust in government influenced COVID-19 perceptions, and provided the platform for the growing misinformation about the pandemic and this in turn shaped vaccine hesitancy.[67,68] Our findings are in agreement with studies in Nigeria which found that non-adherence to recommended preventive measures for COVID-19 was centered on political distrust, stemming from decades of perceived bad governance. [68,69] The mixed perception towards COVID-19 in Nigeria was therefore not surprising and similar controversies have been reported across several regions globally.[70] In times of uncertainty, a coping strategy is to use religion to provide explanations for strange events, [71]and these may conflict with emerging scientific evidence (particularly as conclusions change with new data) and frustrate containment measures. [72] Our findings support the need for inclusive risk communication for epidemic preparedness and control. Moreover, intervention adaptation to suit local contexts is essential during emergency response to

For peer review only - http://bmjopen.bmj.com/site/about/guidelines.xhtml

epidemics. [45] Early reported cases of COVID-19 in the country were among foreigners and high-profile politicians. Linking COVID-19 results to known public officers could have been responsible for the perception that COVID-19 is a disease of the elite. In addition, limited testing capacity could have driven the perception that COVID-19 is not real, as up to 80% of infected individuals had been reported as mild or asymptomatic.[73]

Interestingly, the demand for COVID-19 vaccine was reportedly higher than anticipated among community members despite negative media reports and conspiracy theories. This finding is consistent with a study conducted by Julio et al. which found higher willingness to receive COVID-19 vaccine in low-and-middle-income countries compared to high income countries in which the survey was done.[74] Our findings support the call for vaccine equity, the need for sustained global partnership, and continuous post-vaccination surveillance to achieve effective global vaccination for COVID-19.[75] The concern about the unprecedented short period to vaccine production and licensing underscores the need for sustained and increased efforts toward control of other communicable diseases like tuberculosis, HIV/AIDS, and pneumonia—not neglecting other diseases because of COVID-19. Considering the background mistrust in government, donation of substandard vaccines, and vaccines with short expiry dates or not valid for travel as well as conditional donation of vaccines feeds into public narratives of lack of trust in COVID-19 vaccines and reinforces conspiracy theories about COVID-19. [76–78] Meanwhile, vaccine hesitancy among healthcare providers requires attention for increased and sustained COVID-19 vaccine coverage in the long term. [79]

This study had limitations, firstly we recruited caregivers from PHCs only and did not gather perspectives from other community members. This may mean that the perspectives captured here underestimates negative effects on care-seeking. More so, given that participants were not consulted in the design of the interview guide, we acknowledge that finding from this study may not reflect all aspects considered important to the participants. Review of facility data shows a considerable decrease in out-patient attendance for children (Appendix V). Our findings have provided context-specific understanding of the indirect and direct effects of COVID-related public health measures and may inform future public health responses to disease outbreaks. Though the implementation of lockdown is context-specific, findings from our study may be transferrable to other low and middle-income countries with a similar weak health system and where distrust of government has been a problem.

CONCLUSION

The interpretation of the emergence of a new disease classification is dynamic and multi-faceted. The COVID-19 pandemic in Lagos had both direct and indirect effects on care-seeking for children. It is plausible that these had negative impacts on morbidity and mortality. Subsequent disease outbreak response requires active management of misinformation and intelligent health communication, including context-specific understanding of social-media messaging and the role of religious institutions. Strengthening health and social support system interventions, notably around ensuring access to healthcare is not negatively affected, is crucial to building adaptive capacity for future disease outbreaks, pandemics and building public trust.

For peer review only - http://bmjopen.bmj.com/site/about/guidelines.xhtml

Acknowledgements

We thank the clinical data collectors and facility heads for their support during the data collection, and the caregivers and healthcare workers for giving us their time.

Contributors

AAB, OEO, CK and HMA conceived of the study and TC, CK and AGF are grant holders. AAB designed the study. OEO collected the data with oversight from AAB and OCU. AAB and OEO led the analysis, with support from HMA, CK and HG. The manuscript was drafted by AAB with support from OEO, CK and HMA. All authors contributed to revisions and approved the final manuscript.

Competing interests

SA, TA, CC and PV are employed by Save the Children UK who are part of the partnership funding the research. TFO, MM are employees of GSK, a multinational for-profit pharmaceutical company that produces pharmaceutical products for childhood pneumonia, including a SARS-CoV-2 vaccine, and no direct financial interests in oxygen or pulse oximeter products.

Funding

This work was funded through the GlaxoSmithKline (GSK)-Save the Children Partnership (grant reference: 82603743). Employees of both GSK and Save the Children UK contributed to the design and oversight of the wider INSPIRING study as part of a co-design process but did not take part directly in this sub-study. Any views or opinions presented are solely those of the author/publisher and do not necessarily represent those of Save the Children UK or GSK, unless otherwise specifically stated.

Data availability statement

Data are available upon reasonable request. Transcripts of interviews conducted are available in English may be shared based on nature of request to bakare.ayobami.adebayo@ki.se.

Ethics approval and participant consent

We obtained ethical approvals from the following ethics committees: Lagos State Primary Health Care Board (ref: LS/PHCB/MS/1128/VOL.V1/005), University of Ibadan/University College Hospital (Ref: UI/EC/19/0551) and the University College London (Ref: 3433/005). We obtained informed oral consent from all the participants and conducted the interviews under strict adherence to the study COVID-19 prevention protocol.

For peer review only

References

1. World Health Organization (WHO). Statement on the second meeting of the International Health Regulations (2005) Emergency Committee regarding the outbreak of novel coronavirus (2019-nCoV). Geneva, Switzerland. Published 2020. Accessed December 22, 2022. https://www.who.int/news/item/30-01-2020-statement-on-the-second-meeting-of-the-international-health-regulations-(2005)-emergency-committee-regarding-the-outbreak-of-novel-coronavirus-(2019-ncov)
2. Adams J, MacKenzie MJ, Amegah AK, et al. The conundrum of low covid-19 mortality burden in sub-saharan africa: Myth or reality? *Glob Health Sci Pract*. 2021;9(3):433-443. doi:10.9745/GHSP-D-21-00172
3. Sub-Saharan Africa Exits Recession in 2021 but Recovery Still Vulnerable. Accessed December 8, 2021. https://www.worldbank.org/en/news/press-release/2021/10/06/sub-saharan-africa-exits-recession-in-2021-but-recovery-still-vulnerable
4. World Health Organization. *COVID-19 Weekly Epidemiological Update*. 85th ed.; 2022. Accessed April 2, 2022. https://www.who.int/publications/m/item/weekly-epidemiological-update-on-covid-19---29-march-2022
5. Waogodo Cabore J, Cyprian Karamagi H, Kipchumba Kipruto H, et al. Articles COVID-19 in the 47 countries of the WHO African region: a modelling analysis of past trends and future patterns. *Lancet Glob Health*. Published online 2022. doi:10.1016/S2214-109X(22)00233-9
6. COVID19_Cases. Accessed December 9, 2021. https://who.maps.arcgis.com/apps/dashboards/0c9b3a8b68d0437a8cf28581e9c063a9
7. Lanre R, Bello K, Olatunde O. Easing of lockdown measures in Nigeria: Implications for the healthcare system. *Health Policy Technol*. 2020;9(January):399-404. https://www.ncbi.nlm.nih.gov/pmc/articles/PMC7490626/
8. Amzat J, Aminu K, Kolo VI, Akinyele AA, Ogundairo JA, Danjibo MC. Coronavirus outbreak in Nigeria: Burden and socio-medical response during the first 100 days. *International Journal of Infectious Diseases*. 2020;98(January):218-224. doi:10.1016/j.ijid.2020.06.067
9. Esmé B, Nick G, Max L, Pablo ARM, Anjela T, and Diego AVP. *The Inequality Virus: Bringing Together a World Torn Apart by coronavirus through a Fair, Just and sustainable Economy*. Vol 1. Oxfam GB; 2021.
10. Jensen N, Kelly AH, Avendano M. The COVID-19 pandemic underscores the need for an equity-focused global health agenda. *Humanit Soc Sci Commun*. 2021;8(1). doi:10.1057/s41599-020-00700-x
11. Akande OW, Elimian KO, Igumbor E, et al. Epidemiological comparison of the first and second waves of the COVID-19 pandemic in Nigeria, February 2020–April 2021. *BMJ Glob Health*. 2021;6(11):e007076. doi:10.1136/BMJGH-2021-007076
12. Odusanya OO, Odugbemi BA, Odugbemi TO, Ajisegiri WS. COVID-19: A Review of the Effectiveness of Non-Pharmacological Interventions. *NIgerian Postgraduate Medical Journal*. 2020;27(4):1-7. doi:10.4103/npmj.npmj
13. Verschuur J, Koks EE, Hall JW. Global economic impacts of COVID-19 lockdown measures stand out in highfrequency shipping data. *PLoS One*. 2021;16(4 April):1-16. doi:10.1371/journal.pone.0248818
14. Ozili PK. COVID-19 Pandemic and Economic Crisis: The Nigerian Experience and Structural Causes. *SSRN Electronic Journal*. Published online 2020:1-19. doi:10.2139/ssrn.3567419

For peer review only - http://bmjopen.bmj.com/site/about/guidelines.xhtml

15. Chakraborty I, Maity P. COVID-19 outbreak: Migration, effects on society, global environment and prevention. *Science of the Total Environment*. 2020;728:138882. doi:10.1016/j.scitotenv.2020.138882

16. Conlon C, McDonnell T, Barrett M, et al. The impact of the COVID-19 pandemic on child health and well-being : Are children " slipping through the net " ? A qualitative study of frontline emergency care staff. *BMC Health Serv Res*. 2021;9(1):1-29. https://bmchealthservres.biomedcentral.com/articles/10.1186/s12913-021-06284-9

17. Impact of COVID-19 on people's livelihoods, their health and our food systems. Accessed March 15, 2022. https://www.who.int/news/item/13-10-2020-impact-of-covid-19-on-people%27s-livelihoods-their-health-and-our-food-systems

18. Passavanti M, Argentieri A, Barbieri DM, et al. The psychological impact of COVID-19 and restrictive measures in the world. *J Affect Disord*. 2021;283(September 2020):36-51. doi:10.1016/j.jad.2021.01.020

19. Berhan Y. Will Africa be Devastated by Covid-19 as Many Predicted? Perspective and Prospective. *Ethiop J Health Sci*. 2020;30(3):459-467. doi:10.4314/ejhs.v30i3.17

20. United Nations. *Policy Brief: The Impacts of COVID-19 on Children*. Vol 109.; 2020. doi:10.1111/apa.15484

21. Ashikkali L, Carroll W, Johnson C. The indirect impact of COVID-19 on child health. *Paediatr Child Health*. 2020;30(12):430-437. doi:10.1016/j.paed.2020.09.004

22. Abayomi O. Olaseni, Akinsola OS, Agberotimi SF, Rotimi Oguntayo. Psychological distress experiences of Nigerians during Covid-19 pandemic; the gender difference. *www.archbronconeumol.org Original*. 2020;(January).

23. Ogoina D. Covid-19 and the rest of us. *Niger Delta Medical Journal*. 2020;4(1):6-8.

24. Obi-Ani NA, Anikwenze C, Isiani MC. Social media and the Covid-19 pandemic: Observations from Nigeria. *Cogent Arts Humanit*. 2020;7(1). doi:10.1080/23311983.2020.1799483

25. Johnson OA, Olaniyi SF, John S, et al. "Infodemic" in a pandemic: COVID-19 Conspiracy Theories in an African Country. *Social Health and Behavior*. Published online 2020:19-24. doi:10.4103/SHB.SHB

26. Scaramuzza A, Tagliaferri F, Bonetti L, et al. Changing admission patterns in paediatric emergency departments during the COVID-19 pandemic. *Arch Dis Child*. 2020;105(7):704-706. doi:10.1136/archdischild-2020-319397

27. United Nations. *Transforming Our World: The 2030 Agenda for Sustainble Development*.; 2016.

28. WHO and Maternal and Child Epidemiology Estimation Group (MCEE). Global and Regional Child Deaths by Cause. 2018.

29. Testimony J Olumade, Oluwafolajimi A Adesanya, Iyanuoluwa J Fred-Akintunwa, et al. Infectious disease outbreak preparedness and response in Nigeria_ history, limitations and recommendations for global health policy and practice _ Enhanced Reader. *AIMS Public Health*. 2020;7(4):736-757.

30. Braun V, Clarke V. Reflecting on reflexive thematic analysis. *Qual Res Sport Exerc Health*. 2019;11(4):589-597. doi:10.1080/2159676X.2019.1628806

31. Tong A, Sainsbury P, Craig J. Consolidated criteria for reporting qualitative research (COREQ): a 32-item checklist for interviews and focus groups. 2007;19(6):349-357.

32. About Lagos – Lagos State Government. Accessed February 24, 2022. https://lagosstate.gov.ng/about-lagos/

For peer review only - http://bmjopen.bmj.com/site/about/guidelines.xhtml

33. Presidential task force on COVID-19. *Implementation Guidance for Lockdown Policy*. Vol 5.; 2020. http://www.akrabjuara.com/index.php/akrabjuara/article/view/919
34. Lawal RA, Ibrahim K. *#ENDSARS: Effecting Positive Change in Governance in Nigeria BEYOND M a y 2 0 2 1*. www.bbforpeace.org
35. Graham HR, Olojede OE, Bakare AA, et al. Measuring oxygen access: Lessons from health facility assessments in Lagos, Nigeria. *BMJ Glob Health*. 2021;6(8). doi:10.1136/bmjgh-2021-006069
36. Braun V, Clarke V. Qualitative Research in Psychology Using thematic analysis in psychology Using thematic analysis in psychology. *Qual Res Psychol*. 2006;3(2):77-101. http://www.tandfonline.com/action/journalInformation?journalCode=uqrp20%5Cnhttp://www.tandfonline.com/action/journalInformation?journalCode=uqrp20
37. QSR International Pty Ltd. (2020) NVivo (released in March 2020).
38. Graham HR, Olojede OE, Bakare AAA, et al. Pulse oximetry and oxygen services for the care of children with pneumonia attending frontline health facilities in Lagos, Nigeria (INSPIRING-Lagos): Study protocol for a mixed-methods evaluation. *BMJ Open*. 2022;12(5). doi:10.1136/bmjopen-2021-058901
39. Nigeria records chloroquine poisoning after Trump endorses it for coronavirus treatment - CNN. Accessed January 5, 2022. https://edition.cnn.com/2020/03/23/africa/chloroquine-trump-nigeria-intl/index.html
40. What are the myths about the coronavirus in Africa? | World Economic Forum. Accessed January 5, 2022. https://www.weforum.org/agenda/2020/04/debunking-9-popular-myths-doing-the-rounds-in-africa-about-the-coronavirus/
41. Winters M, Oppenheim B, Sengeh P, et al. Debunking highly prevalent health misinformation using audio dramas delivered by WhatsApp: Evidence from a randomised controlled trial in Sierra Leone. *BMJ Glob Health*. 2021;6(11). doi:10.1136/bmjgh-2021-006954
42. Roberto A, Sellon A, Cherry ST, Hunter-Jones J, Winslow H. Impact of spirituality on resilience and coping during the COVID-19 crisis: A mixed-method approach investigating the impact on women. *https://doi.org/101080/0739933220201832097*. 2020;41(11-12):1313-1334. doi:10.1080/07399332.2020.1832097
43. Ossai EC. 'It is the antichrist. Can't you see?' Perceptions of COVID-19 among Nigeria's Christians and the Religion—Health Debate. *https://doi.org/103366/swc20210325*. 2021;27(1):48-64. doi:10.3366/SWC.2021.0325
44. Barry S Hewlett, Richard P. Amola. Cultural Contexts of Ebola in Northern Uganda. *Emerg Infect Dis*. 2003;9(10).
45. Owoyemi A, Okolie EA, Omitiran K, et al. Importance of community-level interventions during the COVID-19 pandemic: Lessons from sub-saharan Africa. *American Journal of Tropical Medicine and Hygiene*. 2021;105(4):879-883. doi:10.4269/ajtmh.20-1533
46. Dash S, Parray AA, de Freitas L, et al. Combating the COVID-19 infodemic: A three-level approach for low and middle-income countries. *BMJ Glob Health*. 2021;6(1). doi:10.1136/bmjgh-2020-004671
47. Briggs D, Kattey K. COVID-19 : Parents ' Healthcare-Seeking Behaviour for their Sick Children in COVID-19 : Parents ' Healthcare-Seeking Behaviour for their Sick Children in Nigeria - An Online Survey. 2020;(September). doi:10.9734/IJTDH/2020/v41i1330344

For peer review only - http://bmjopen.bmj.com/site/about/guidelines.xhtml

48. Tan CD, Lutgert EK, Neill S, et al. Parents ' experiences with a sick or injured child during the COVID-lockdown : an online survey in the Netherlands. Published online 2021:1-7. doi:10.1136/bmjopen-2021-055811
49. Watson G, Pickard L, Williams B, Hargreaves D, Blair M. Do I, don't I?' A qualitative study addressing parental perceptions about seeking healthcare during the COVID-19 pandemic. *Arch Dis Child*. 2021;106(11):1118-1124. doi:10.1136/archdischild-2020-321260
50. Kansiime MK, Tambo JA, Mugambi I, Bundi M, Kara A, Owuor C. COVID-19 implications on household income and food security in Kenya and Uganda: Findings from a rapid assessment. *World Dev*. 2021;137. doi:10.1016/j.worlddev.2020.105199
51. Osendarp S, Akuoku JK, Black RE, et al. The COVID-19 crisis will exacerbate maternal and child undernutrition and child mortality in low- and middle-income countries. *Nat Food*. 2021;2(7):476-484. doi:10.1038/s43016-021-00319-4
52. Emilia Connolly, Emma J Boley, Donald Luke Fejfar, et al. Childhood immunization during the COVID-19 pandemic: experiences in Haiti, Lesotho, Liberia and Malawi. *Bull World Health Organization*. Published online 2022.
53. Zhong Y, Clapham HE, Aishworiya R, et al. Childhood vaccinations: Hidden impact of COVID-19 on children in Singapore. *Vaccine*. 2021;39(5):780-785. doi:10.1016/J.VACCINE.2020.12.054
54. Chandir S, Siddiqi DA, Setayesh H, Khan AJ. Impact of COVID-19 lockdown on routine immunisation in Karachi, Pakistan. *Lancet Glob Health*. 2020;8(9):e1118-e1120. doi:10.1016/S2214-109X(20)30290-4/ATTACHMENT/620A1068-4BDB-41AC-9C3C-14F0A5E00589/MMC1.PDF
55. McDonald HI, Tessier E, White JM, et al. Early impact of the coronavirus disease (COVID-19) pandemic and physical distancing measures on routine childhood vaccinations in England, January to April 2020. *Eurosurveillance*. 2020;25(19):2000848. doi:10.2807/1560-7917.ES.2020.25.19.2000848/CITE/PLAINTEXT
56. Iwuoha VC, Aniche ET, Obiora CA, UMeifekwem UT. Citizens lack access to healthcare facilities_ How COVID-19 lockdown and social distancing policies boost roadside chemist businesses in South-Eastern Nigeria _ Enhanced Reader. *International Journal of Health Planning Management*. Published online 2021.
57. Davis AL, Sunderji A, Marneni SR, et al. Caregiver-reported delay in presentation to pediatric emergency departments for fear of contracting COVID-19: a multi-national cross-sectional study. *Canadian Journal of Emergency Medicine*. 2021;23(6):778-786. doi:10.1007/s43678-021-00174-z
58. Lazzerini M, Barbi E, Apicella A, Marchetti F, Cardinale F, Trobia G. Delayed access or provision of care in Italy resulting from fear of COVID-19. *Lancet Child Adolesc Health*. 2020;4(5):e10-e11. doi:10.1016/S2352-4642(20)30108-5
59. Ciacchini B, Tonioli F, Marciano C, et al. Reluctance to seek pediatric care during the COVID-19 pandemic and the risks of delayed diagnosis. *Ital J Pediatr*. 2020;46(1):1-4. doi:10.1186/s13052-020-00849-w
60. Singh DR, Sunuwar DR, Shah SK, et al. Impact of COVID-19 on health services utilization in Province-2 of Nepal: a qualitative study among community members and stakeholders. *BMC Health Serv Res*. 2021;21(1):1-14. doi:10.1186/s12913-021-06176-y
61. Wegbom AI, Edet CK, Raimi O, Fagbamigbe AF, Kiri VA. Self-Medication Practices and Associated Factors in the Prevention and/or Treatment of COVID-19 Virus: A Population-Based Survey in Nigeria. *Front Public Health*. 2021;9(June):1-9. doi:10.3389/fpubh.2021.606801

For peer review only - http://bmjopen.bmj.com/site/about/guidelines.xhtml

62. Onchonga D, Omwoyo J, Nyamamba D. Assessing the prevalence of self-medication among healthcare workers before and during the 2019 SARS-CoV-2 (COVID-19) pandemic in Kenya. *Saudi Pharmaceutical Journal*. 2020;28(10):1149-1154. doi:10.1016/j.jsps.2020.08.003
63. Osaigbovo L, Ogboghodo E, Obaseki D, et al. Pattern of Drug Sales At Community Pharmacies in Edo State As Evidence of Self-Medication During the Covid-19 Pandemic: Implications for Policy Implementtation. *J Chem Inf Model*. 2020;20(04):150-158.
64. Hedstrom A, Mubiri P, Nyonyintono J, et al. Impact of the early COVID-19 pandemic on outcomes in a rural Ugandan neonatal unit: A retrospective cohort study. *PLoS One*. 2021;16(12 December). doi:10.1371/JOURNAL.PONE.0260006
65. *Maintaining the Provision and Use of Services for Maternal, during the COVID-19 Pandemic: Lessons Learned from 19 Countries*.; 2021. licence: CC BY-NC-SA 3.0 IGO.
66. Kadambari S, Goldacre R, Morris E, Goldacre MJ, Pollard AJ. Indirect effects of the covid-19 pandemic on childhood infection in England: population based observational study. *BMJ*. Published online 2022. doi:10.1136/bmj-2021-067519
67. Agbo UM, Nche GC. Suspecting the Figures: What Church Leaders Think About Government's Commitment to Combating COVID-19 in Nigeria. *J Asian Afr Stud*. Published online 2022. doi:10.1177/00219096211069645
68. Wonodi C, Obi-Jeff C, Adewumi F, et al. Conspiracy theories and misinformation about COVID-19 in Nigeria: Implications for vaccine demand generation communications. *Vaccine*. 2022;40(13):2114-2121. doi:10.1016/J.VACCINE.2022.02.005
69. Ezeibe CC, Ilo C, Ezeibe EN, et al. Political distrust and the spread of COVID-19 in Nigeria. *Glob Public Health*. 2020;15(12):1753-1766. doi:10.1080/17441692.2020.1828987
70. Hardy LJ. *Connection, Contagion, and COVID-19*.
71. Adiyoso W, Kanegae H. The Preliminary Study of the Role of Islamic Teaching in the Disaster Risk Reduction (A Qualitative Case Study of Banda Aceh, Indonesia). *Procedia Environ Sci*. 2013;17:918-927. doi:10.1016/j.proenv.2013.02.110
72. Yau EKB, Ping NPT, Shoesmith WD, James S, Hadi NMN, Lin LJ. The behaviour changes in response to COVID-19 pandemic within Malaysia. *Malaysian Journal of Medical Sciences*. 2020;27(2):45-50. doi:10.21315/mjms2020.27.2.5
73. Wu Z, McGoogan J. Characteristics of and Important Lessons From the Coronavirus Disease 2019 (COVID-19) Outbreak in China: Summary of a Report of 72 314 Cases From the Chinese Center for Disease Control and Prevention. *JAMA*. Published online 2020.
74. Solís Arce JS, Warren SS, Meriggi NF, et al. COVID-19 vaccine acceptance and hesitancy in low- and middle-income countries. *Nat Med*. 2021;27(8):1385-1394. doi:10.1038/s41591-021-01454-y
75. Forman R, Shah S, Jeurissen P, Jit M, Mossialos E. COVID-19 vaccine challenges: What have we learned so far and what remains to be done? *Health Policy (New York)*. 2021;125(5):553-567. doi:10.1016/j.healthpol.2021.03.013
76. Statement on Covishield and the European Union (EU) Digital COVID Certificate "Green Pass" – Africa CDC. Accessed June 27, 2022. https://africacdc.org/download/statement-on-covishield-and-the-european-union-eu-digital-covid-certificate-green-pass/
77. Joint Statement on Dose Donations of COVID-19 Vaccines to African Countries. Accessed June 27, 2022. https://www.who.int/news/item/29-11-2021-joint-statement-on-dose-donations-of-covid-19-vaccines-to-african-countries

For peer review only - http://bmjopen.bmj.com/site/about/guidelines.xhtml

78. Lucien Hordijk, Priti Patnaik. Covid-19-How Europe's vaccine donations went tragically wrong. doi:10.1136/bmj.o1286

79. Ackah M, Ameyaw L, Gazali M, et al. COVID-19 vaccine acceptance among health care workers in Africa: A systematic review and meta-analysis. *PLoS One*. 2022;17(5):e0268711. doi:10.1371/JOURNAL.PONE.0268711

For peer review only

Appendix I: -Ease of COVID – 19 Lockdown in Nigeria

	Phase 1	Phase 2	Phase 3	Phase 4	Phase 5
**Start date**<br><br>**End date**	4th May 2020<br><br>1st June 2020	2nd June 2020<br><br>18th October 2020	19th October 2020	11th May 2021	2nd April 2022
**Land travel (Interstate)**	Banned except for essential services and movement of goods and services only	Open under strict conditions: Allowed for essential services and movement of goods and services only	Open	Open	Open
**Land travel (Intrastate)**	Limited to 6 am-6 pm with a 50% reduction in bus occupancy	Open	Open	Open	Open
**Airspace**	Closed for most passenger flights. Open to cargo and specially approved flights only	Open for domestic flights, limited for essential international flights until August 26	Open for domestic and international flights	Open for domestic and international flights	Open for domestic and international flights
**Movement**	Curfew from 8pm to 6am	Curfew from 10pm – 4am	12am to 4am	Curfew from 12am – 4am	No restrictions
**Working hours**	9 am to 2 pm	9 am – 2 pm for Government/other corporate offices	All government staff on grade level 12 and below to continue staying at home<br><br>No limit for private and other corporate bodies	All government staff on grade level 12 and below to continue staying at home until 11th June 2021<br><br>No limit for private and other corporate bodies	No restrictions
**Workspace**	50% staff occupancy or less	75% staff occupancy or less<br>50% for clients	100% occupancy	No limits but virtual meetings and work from home encouraged	No restrictions
**Entertainment activities**	Banned	Banned	Open	Open with some restrictions (bars, night clubs, pubs remained closed)	Open at 50% capacity
**Mass gathering**	Limited to 20 people or less	Limited to 20 people or less	Limited to 50 people or less	Limited to 50 people or less except with permission	Open

For peer review only

				from the state government	
**Religious gathering**	Restricted	Restricted	Restricted (subject to the protocol from the state government and the federal capital territory	Limited to less than 50% capacity<br><br>Gathering more than 50 people must be held outdoors only	Open
**Schools**	Closed	Closed, but special consideration for graduation exams	Open	Open	Open
**Markets**	Partial closure (opened only on designated days weekly between 8.00 am-3.00 pm)	Controlled access by local authorities	Open	Open	Open
**Face masks**	Mandatory for all persons in public spaces	Mandatory for all persons in public spaces	Mandatory for all persons in public spaces	Mandatory for all persons in public spaces	Mandatory for indoor activities only, but at individual discretion for outdoor activities
**Banks and other financial institutions**	Limited staff physically to between 30%-50%	Limited staff physically to 75% or less. Operated for normal working hours	Open	Open	Open

Source: NCDC Coronavirus COVID-19 Microsite. Accessed June 29, 2022. https://covid19.ncdc.gov.ng/guideline/

- *There was a total lockdown of economic activities in the FCT, Lagos and Ogun states for 35days from 30th March 2020. This was coupled with a total ban on non-essential interstate travels*
- *From the third phase, the end dates were assumed as the onset of the next phase*
- *Data collection was done during the phase 3*
- *The second wave of infection and vaccine rollout started during the phase 3*

For peer review only - http://bmjopen.bmj.com/site/about/guidelines.xhtml

Appendix II: In-depth interview guide for healthcare provider's interviews

1. Tell me about the facility you work in?
    a. What type of services do you offer children?
    b. Tell me specifically about this week in your clinic
2. Think about last 8 months, has things been typical? Why? Why not?
    a. When did you first hear about covid?
    b. When did you make adaptation or adjustment in your facility as a result of covid?
    c. What changes did your facility make?

NOW WE WANT TO FOCUS ON QUESTIONS REGARDING CHILDREN

3. Tell me how the lockdown in year 2020 affected service provision at your facility
    a. How did it affect services you provide for children?
    a. How did it affect care seeking for sick under-five?
        - Probe severity of illness at presentation/late presentation
        - Was the PHC the first point of call?

4. Thinking about this time last year, before covid/EndSARS, is there any differences? What is different (numbers, type of presentation, services provided, resources), what is the same?
5. Now that lockdown is over, have things normalized the way it used to be before COVID-19? What has normalized? What is yet to normalize. What about number of under-five that you see, any difference compared to last year in terms of number, type of presentation
6. Late in last year, there was Endsars protest. How did it affect service delivery in your facility?
7. Currently, there is second wave of Covid-19 in Nigeria. How has it affected service delivery in your facility?
8. What can you say about the care seeking behavior of caregivers of sick children you have attended to in recent times?
9. Between Covid-19 lockdown, End-Sars protest and current economic hardship, which one has affected care seeking for sick children most? Why? Short term consequences? Any long term consequences?
10. Finally, the federal government is making plans to procure Covid-19 vaccines for Nigerians. How willing are you to receive the vaccine? Why/why not? What about for your child/children? Why/why not? Will you tell others to take it? Why?
11. Do you have any other things to say?

For peer review only

Appendix III: In – Depth interview guide for caregiver of under-five with recent illness episode

1. Tell me about your family.
   Probe to get information on:
   a. Who lives with the participant
   b. Participant's job
   c. Where participant's extended family live
   d. Involvement in child's care
2. How will you summarize year 2020?
   Probe:
   a. How did it affect you and your family?
   b. Could you say these changes were due to impact of covid-19 pandemic?
      1. If yes, why? In what other ways have covid-19 affected you and your household
      2. If no, why not?
   c. Have you noticed changes in the price of commodities?
      i. How does this affect you and your household?
         1. House rent
         2. Transport cost
         3. Food items

Now we want to talk about child health services, particularly care seeking for sick under-five

3. Your child was recently sick; I would like to know more about the illness.
   a. How did it start? Who first noticed the symptoms?
   b. What did you do first? When did you do that?
   c. What next did you do?
      i. How did you decide?
      ii. Why did you do that? Could you have done something else?
      iii. What treatment were given? Was your child asked to do some tests? Could you afford all the test?
      iv. Were you referred?
         1. If yes, did you honour the referral? Why?
         2. How did you feel with the referral?
         3. If no, why? What did you do next? Why did you do that?
         4. Was your child asked to do some tests? Could you afford all the test?
         5. What about medications? Did you buy all the medication?
      v. Like how much did it cost you to treat your child? Would the cost have been cheaper if not for current situations? How did you cover the cost of treatments for your child?
         1. Personal money/savings?
         2. Support from father?
         3. Support family and friends?
         4. Did you have to borrow or sell any items?

4. Overall, has covid-19 affected your decisions and steps when your child was sick?

   i. If yes, how?
   ii. If no, what affected your decisions and steps?
      i. Endsars protest?

For peer review only

ii. Insecurity?
iii. Current economic hardship?
iv. Could you have taken different actions/steps (*relate this to previous answers*) What about fears of catching covid at the hospital?

5. Finally, the federal government has indicated that by Jan 2021 the country will have covid-19 vaccine. How willing are you to receive the vaccine? Why/why not? What about for your child/children? Why/why not?
6. Do you have other things to say or bring to my attention? Thank you for your time!

For peer review only

Appendix IV: IDI guide for No illness episode

1. Tell me about your family.
   Probe to get information on:
   e. Who lives with the participant.
   f. Participant's job
   g. Where participant's extended family live
   h. Involvement in child's care
2. How will you summarize year 2020?
   Probe:
   a. How has it affected you and your family?
   b. Were these covid-19 related?
      i. If yes, why and how? In what **other** ways have covid-19 affected you and your household
      ii. If no, what do you think is responsible?
   c. Have you noticed changes in the price of commodities?
      i. How does this affect you and your household?
         1. House rent
         2. Transport cost
         3. Food items
         4. Fuel price
3. Now we want to talk about care seeking for under-five. What actions do mothers/caregivers take when their child develops illness?
   i. Why do they do that?
      1. Could it be because of trust/distrust in health care workers?
      2. Could cost have influenced their decision? How?
      3. What else could have influenced their action?
   ii. Think about the last time your child (or that of someone close to you) fell sick
      1. What was wrong? What did you do? How did you decide on what to do? Who did you talk to? What alternatives were considered? What were your concerns?
      2. Was your child referred?
      3. Did you honour the referral?
      4. If yes, why? If no, why?
      5. If it happens this period, could you or they have taken different action? Why?
   iii. During the covid-pandemic in Nigeria, do you think covid-19 affected decisions taken by caregivers when their child was sick? if yes, why and how? If no, why?
   iv. What about now? Do covid-19 affect actions taken by mothers when their child falls sick?
   v. Between covid-19 and current economic hardship, which one has greater influence on actions taken by caregivers when their child is sick?
      1. Why and how?
4. Finally, the federal government has indicated that by Jan 2021 the country will have covid-19 vaccine. How willing are you to receive the vaccine? Why/why not? What about for your child/children? Why/why not?
5. Do you have any other thing to tell me?

Thank you for your time!

For peer review only

Appendix V Outpatient attendance for under-five children in the 7 flagship facilities in Ikorodu LGA (January-June 2020)*

**Year**	**Flagship PHCs**													
2020	**Ikorodu**		**Igbogbo**		**Odonla**		**Agbede**		**Ipakodo**		**Imota**		**Oke-Eletu¥**	
**January—March**	**Diagnosis**	**Number**	**Diagnosis**	**Number**	**Diagnosis**	**Number**	**Diagnosis**	**Number**	**Diagnosis**	**Number**	**Diagnosis**	**Number**	**Diagnosis**	**Number**
	Pneumonia	9	Pneumonia	3	Pneumonia	1	Pneumonia	0	Pneumonia	3	Pneumonia	19	Pneumonia	-
	LRTI	7	LRTI	12	LRTI	1	LRTI	0	LRTI	1	LRTI	2	LRTI	-
	URTI	133	URTI	290	URTI	89	URTI	0	URTI	102	URTI	47	URTI	-
	ARTI	0	ARTI	0	ARTI	0	ARTI	0	ARTI	5	ARTI	0	ARTI	-
	Malaria	511	Malaria	275	Malaria	149	Malaria	129	Malaria	234	Malaria	125	Malaria	-
	Sepsis	43	Sepsis	97	Sepsis	243	Sepsis	21	Sepsis	42	Sepsis	33	Sepsis	-
	Others	274	Others	374	Others	406	Others	115	Others	211	Others	252	Others	-
	**Total**	**977**	**Total**	**1051**	**Total**	**892**	**Total**	**265**	**Total**	**589**	**Total**	**478**	**Total**	-
**April—June**	Pneumonia	1	Pneumonia	0	Pneumonia	2	Pneumonia	0	Pneumonia	0	Pneumonia	1	Pneumonia	0
	LRTI	3	LRTI	1	LRTI	0	LRTI	0	LRTI	0	LRTI	0	LRTI	0
	URTI	10	URTI	55	URTI	5	URTI	1	URTI	25	URTI	2	URTI	34
	ARTI	0	ARTI	0	ARTI	0	ARTI	0	ARTI	0	ARTI	0	ARTI	0
	Malaria	215	Malaria	183	Malaria	64	Malaria	23	Malaria	39	Malaria	52	Malaria	42
	Sepsis	22	Sepsis	26	Sepsis	55	Sepsis	2	Sepsis	9	Sepsis	8	Sepsis	63
	Others	113	Others	39	Others	159	Others	5	Others	99	Others	125	Others	164
	**Total**	**364**	**Total**	**304**	**Total**	**285**	**Total**	**31**	**Total**	**172**	**Total**	**188**	**Total**	**303**

*Lagos placed on lockdown on the 30 March 2020

¥ Facility register not found

For peer review only

International Journal for Quality in Health Care; Volume 19, Number 6: pp. 349–357 10.1093/intqhc/mzm042
Advance Access Publication: 14 September 2007

Consolidated criteria for reporting qualitative research (COREQ): a 32-item checklist for interviews and focus groups

ALLISON TONG[1,2], PETER SAINSBURY[1,3] AND JONATHAN CRAIG[1,2]

[1]School of Public Health, University of Sydney, NSW 2006, Australia, [2]Centre for Kidney Research, The Children's Hospital at Westmead, NSW 2145, Australia, and [3]Population Health, Sydney South West Area Health Service, NSW 2170, Australia

Abstract

Background. Qualitative research explores complex phenomena encountered by clinicians, health care providers, policy makers and consumers. Although partial checklists are available, no consolidated reporting framework exists for any type of qualitative design.

Objective. To develop a checklist for explicit and comprehensive reporting of qualitative studies (indepth interviews and focus groups).

Methods. We performed a comprehensive search in Cochrane and Campbell Protocols, Medline, CINAHL, systematic reviews of qualitative studies, author or reviewer guidelines of major medical journals and reference lists of relevant publications for existing checklists used to assess qualitative studies. Seventy-six items from 22 checklists were compiled into a comprehensive list. All items were grouped into three domains: (i) research team and reflexivity, (ii) study design and (iii) data analysis and reporting. Duplicate items and those that were ambiguous, too broadly defined and impractical to assess were removed.

Results. Items most frequently included in the checklists related to sampling method, setting for data collection, method of data collection, respondent validation of findings, method of recording data, description of the derivation of themes and inclusion of supporting quotations. We grouped all items into three domains: (i) research team and reflexivity, (ii) study design and (iii) data analysis and reporting.

Conclusions. The criteria included in COREQ, a 32-item checklist, can help researchers to report important aspects of the research team, study methods, context of the study, findings, analysis and interpretations.

Keywords: focus groups, interviews, qualitative research, research design

Qualitative research explores complex phenomena encountered by clinicians, health care providers, policy makers and consumers in health care. Poorly designed studies and inadequate reporting can lead to inappropriate application of qualitative research in decision-making, health care, health policy and future research.

Formal reporting guidelines have been developed for randomized controlled trials (CONSORT) [1], diagnostic test studies (STARD), meta-analysis of RCTs (QUOROM) [2], observational studies (STROBE) [3] and meta-analyses of observational studies (MOOSE) [4]. These aim to improve the quality of reporting these study types and allow readers to better understand the design, conduct, analysis and findings of published studies. This process allows users of published research to be more fuller informed when they critically appraise studies relevant to each checklist and decide upon applicability of research findings to their local settings. Empiric studies have shown that the use of the CONSORT statement is associated with improvements in the quality of reports of randomized controlled trials [5]. Systematic reviews of qualitative research almost always show that key aspects of study design are not reported, and so there is a clear need for a CONSORT-equivalent for qualitative research [6].

The Uniform Requirements for Manuscripts Submitted to Biomedical Journals published by the International Committee of Medical Journal Editors (ICMJE) do not provide reporting guidelines for qualitative studies. Of all the mainstream biomedical journals (Fig. 1), only the British Medical Journal (BMJ) has criteria for reviewing qualitative research. However, the guidelines for authors specifically record that the checklist is not routinely used. In addition, the checklist is not comprehensive and does not provide specific guidance to assess some of the criteria. Although checklists for critical appraisal are available for qualitative research, there is no widely endorsed reporting framework for any type of qualitative research [7].

We have developed a formal reporting checklist for in-depth interviews and focus groups, the most common methods for data collection in qualitative health research.

Address reprint requests to: Allison Tong, Centre for Kidney Research, The Children's Hospital at Westmead, NSW 2145, Australia. Tel: +61-2-9845-1482; Fax: +61-2-9845-1491; E-mail: allisont@health.usyd.edu.au, allisont@chw.edu.au

© The Author 2007. Published by Oxford University Press on behalf of International Society for Quality in Health Care; all rights reserved

For peer review only - http://bmjopen.bmj.com/site/about/guidelines.xhtml

For peer review only

Downloaded from https://academic.oup.com/intqhc/article/19/6/349/1791966 by guest on 31 October 2021

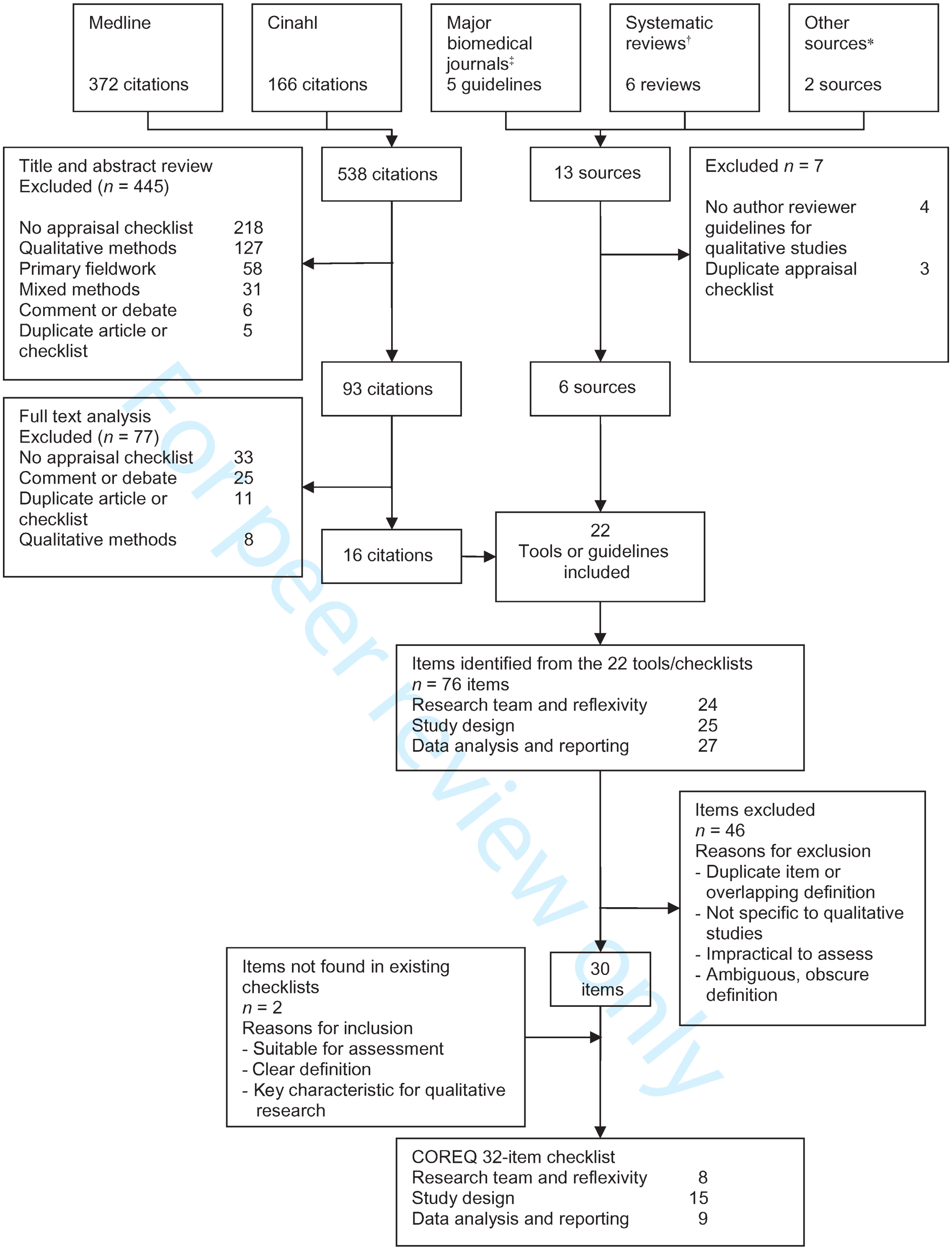

Figure 1 Development of the COREQ Checklist. *References [26, 27], †References [6, 28–32], ‡Author and reviewer guidelines provided by BMJ, JAMA, Lancet, Annals of Internal Medicine, NEJM.

These two methods are particularly useful for eliciting patient and consumer priorities and needs to improve the quality of health care [8]. The checklist aims to promote complete and transparent reporting among researchers and indirectly improve the rigor, comprehensiveness and credibility of interview and focus-group studies.

Basic definitions

Qualitative studies use non-quantitative methods to contribute new knowledge and to provide new perspectives in health care. Although qualitative research encompasses a broad range of study methods, most qualitative research

For peer review only

Downloaded from https://academic.oup.com/intqhc/article/19/6/349/1791966 by guest on 31 October 2021

publications in health care describe the use of interviews and focus groups [8].

Interviews

In-depth and semi-structured interviews explore the experiences of participants and the meanings they attribute to them. Researchers encourage participants to talk about issues pertinent to the research question by asking open-ended questions, usually in one-to-one interviews. The interviewer might re-word, re-order or clarify the questions to further investigate topics introduced by the respondent. In qualitative health research, in-depth interviews are often used to study the experiences and meanings of disease, and to explore personal and sensitive themes. They can also help to identify potentially modifiable factors for improving health care [9].

Focus groups

Focus groups are semi-structured discussions with groups of 4–12 people that aim to explore a specific set of issues [10]. Moderators often commence the focus group by asking broad questions about the topic of interest, before asking the focal questions. Although participants individually answer the facilitator's questions, they are encouraged to talk and interact with each other [11]. This technique is built on the notion that the group interaction encourages respondents to explore and clarify individual and shared perspectives [12]. Focus groups are used to explore views on health issues, programs, interventions and research.

Methods

Development of a checklist

Search strategy. We performed a comprehensive search for published checklists used to assess or review qualitative studies, and guidelines for reporting qualitative studies in: Medline (1966—Week 1 April 2006), CINAHL (1982—Week 3 April 2006), Cochrane and Campbell protocols, systematic reviews of qualitative studies, author or reviewer guidelines of major medical journals and reference lists of relevant publications. We identified the terms used to index the relevant articles already in our possession and performed a broad search using those search terms. The electronic databases were searched using terms and text words for research (standards), health services research (standards) and qualitative studies (evaluation). Duplicate checklists and detailed instructions for conducting and analysing qualitative studies were excluded.

Data extraction. From each of the included publications, we extracted all criteria for assessing or reporting qualitative studies. Seventy-six items from 22 checklists were compiled into a comprehensive list. We recorded the frequency of each item across all the publications. Items most frequently included in the checklists related to sampling method, setting for data collection, method of data collection, respondent validation of findings, method of recording data, description of the derivation of themes and inclusion of supporting quotations. We grouped all items into three domains: (i) research team and reflexivity, (ii) study design and (iii) data analysis and reporting. (see Tables 2–4)

Within each domain we simplified all relevant items by removing duplicates and those that were ambiguous, too broadly defined, not specific to qualitative research, or impractical to assess. Where necessary, the remaining items were rephrased for clarity. Based upon consensus among the authors, two new items that were considered relevant for reporting qualitative research were added. The two new items were identifying the authors who conducted the interview or focus group and reporting the presence of non-participants during the interview or focus group. The COREQ checklist for explicit and comprehensive reporting of qualitative studies consists of 32 criteria, with a descriptor to supplement each item (Table 1).

COREQ: content and rationale (see Tables 1)

Domain 1: research team and reflexivity

(i) Personal characteristics: Qualitative researchers closely engage with the research process and participants and are therefore unable to completely avoid personal bias. Instead researchers should recognize and clarify for readers their identity, credentials, occupation, gender, experience and training. Subsequently this improves the credibility of the findings by giving readers the ability to assess how these factors might have influenced the researchers' observations and interpretations [13–15].

(ii) Relationship with participants: The relationship and extent of interaction between the researcher and their participants should be described as it can have an effect on the participants' responses and also on the researchers' understanding of the phenomena [16]. For example, a clinician–researcher may have a deep understanding of patients' issues but their involvement in patient care may inhibit frank discussion with patient–participants when patients believe that their responses will affect their treatment. For transparency, the investigator should identify and state their assumptions and personal interests in the research topic.

Domain 2: study design

(i) Theoretical framework: Researchers should clarify the theoretical frameworks underpinning their study so readers can understand how the researchers explored their research questions and aims. Theoretical frameworks in qualitative research include: grounded theory, to build theories from the data; ethnography, to understand the culture of groups with shared characteristics; phenomenology, to describe the meaning and significance of experiences; discourse analysis, to analyse linguistic expression; and content analysis, to systematically organize data into a structured format [10].

For peer review only

Downloaded from https://academic.oup.com/intqhc/article/19/6/349/1791966 by guest on 31 October 2021

Table 1 Consolidated criteria for reporting qualitative studies (COREQ): 32-item checklist

No	Item	Guide questions/description	
**Domain 1: Research team and reflexivity**			
Personal Characteristics			
1.	Interviewer/facilitator	Which author/s conducted the interview or focus group?	Page 7
2.	Credentials	What were the researcher's credentials? *E.g. PhD, MD*	Page 7
3.	Occupation	What was their occupation at the time of the study?	Page 7
4.	Gender	Was the researcher male or female?	Page 7
5.	Experience and training	What experience or training did the researcher have?	Page 7
Relationship with participants			
6.	Relationship established	Was a relationship established prior to study commencement?	Page 7
7.	Participant knowledge of the interviewer	What did the participants know about the researcher? e.g. *personal goals, reasons for doing the research*	Page 7
8.	Interviewer characteristics	What characteristics were reported about the interviewer/facilitator? e.g. *Bias, assumptions, reasons and interests in the research topic*	Page 7
**Domain 2: study design**			
Theoretical framework			
9.	Methodological orientation and Theory	What methodological orientation was stated to underpin the study? *e.g. grounded theory, discourse analysis, ethnography, phenomenology, content analysis*	Page 5
Participant selection			
10.	Sampling	How were participants selected? *e.g. purposive, convenience, consecutive, snowball*	Page 6
11.	Method of approach	How were participants approached? e.*g. face-to-face, telephone, mail, email*	Page 6
12.	Sample size	How many participants were in the study?	Page 6
13.	Non-participation	How many people refused to participate or dropped out? Reasons?	Page 7
Setting			
14.	Setting of data collection	Where was the data collected? e.*g. home, clinic, workplace*	Page 6
15.	Presence of non-participants	Was anyone else present besides the participants and researchers?	Page 7
16.	Description of sample	What are the important characteristics of the sample? *e.g. demographic data, date*	Pages 6,7
Data collection			
17.	Interview guide	Were questions, prompts, guides provided by the authors? Was it pilot tested?	Yes, see supplementary materials
18.	Repeat interviews	Were repeat interviews carried out? If yes, how many?	Page 8
19.	Audio/visual recording	Did the research use audio or visual recording to collect the data?	Page 7
20.	Field notes	Were field notes made during and/or after the interview or focus group?	Page 7
21.	Duration	What was the duration of the interviews or focus group?	Page 8
22.	Data saturation	Was data saturation discussed?	Page 8
23.	Transcripts returned	Were transcripts returned to participants for comment and/or correction?	Page 8
**Domain 3: analysis and findings**z			
Data analysis			
24.	Number of data coders	How many data coders coded the data?	Page 8
25.	Description of the coding tree	Did authors provide a description of the coding tree?	page 7
26.	Derivation of themes	Were themes identified in advance or derived from the data?	Page 8
27.	Software	What software, if applicable, was used to manage the data?	Page 8
28.	Participant checking	Did participants provide feedback on the findings?	Page 8
Reporting			
29.	Quotations presented	Were participant quotations presented to illustrate the themes / findings? Was each quotation identified? e.*g. participant number*	Pages 11 and 12
30.	Data and findings consistent	Was there consistency between the data presented and the findings?	Pages 11 and 12
31.	Clarity of major themes	Were major themes clearly presented in the findings?	Pages 11 and 12
32.	Clarity of minor themes	Is there a description of diverse cases or discussion of minor themes?	Pages 11 and 12

(ii) Participant selection: Researchers should report how participants were selected. Usually purposive sampling is used which involves selecting participants who share particular characteristics and have the potential to provide rich, relevant and diverse data pertinent to the research question [13, 17]. Convenience sampling is less optimal because it may fail to capture important perspectives from difficult-to-reach people [16]. Rigorous attempts to recruit participants and reasons for non-participation should be stated to reduce the likelihood of making unsupported statements [18].

For peer review only - http://bmjopen.bmj.com/site/about/guidelines.xhtml

For peer review only

Downloaded from https://academic.oup.com/intqhc/article/19/6/349/1791966 by guest on 31 October 2021

Table 2 Items included in 22 published checklists: Research team and reflexivity domain

Item	References																					
	[26][a]	[27][a]	[6][b]	[28][b]	[32][b]	[13]	[15]	[14]	[17]	[33]	[34]	[35]	[16]	[19]	[36]	[7]	[37]	[23]	[38]	[39]	[22]	BMJ
Research team and reflexivity																						
Nature of relationship between the researcher and participants		•		•	•		•		•						•				•			
Examination of role, bias, influence	•	•			•	•	•	•							•						•	
Description of role		•		•					•	•				•	•					•	•	
Identity of the interviewer		•		•		•					•		•		•							
Continued and prolonged engagement		•				•							•	•					•	•		
Response to events	•	•				•	•	•														
Prior assumptions and experience		•						•									•			•		
Professional status		•					•								•							
Journal, record of personal experience		•								•				•								
Effects of research on researcher		•				•	•															
Qualifications		•													•							
Training of the interviewer/facilitator			•		•																	
Expertise demonstrated		•																	•			
Perception of research at inception								•						•								
Age							•															
Gender							•															
Social class							•															
Reasons for conducting study		•																				
Sufficient contact													•									
Too close to participants													•									
Empathy																	•					
Distance between researcher and participants							•															
Background								•														
Familiarity with setting																					•	

[a]Other publications, [b]Systematic review of qualitative studies; BMJ, British Medical Journal—editor's checklist for appraising qualitative research); •, item included in the checklist.

Downloaded from https://academic.oup.com/intqhc/article/19/6/349/1791966 by guest on 31 October 2021

For peer review only

Table 3 Items included in 22 published checklists: Study design

Item	[26][a]	[27][a]	[6][b]	[28] [b]	[32][b]	[13]	[15]	[14]	[17]	[33]	[34]	[35]	[16]	[19]	[36]	[7]	[37]	[23]	[38]	[39]	[22]	BMJ
Study design																						
Methodological orientation, ontological or epistemological basis		•		•				•	•					•					•	•	•	•
Sampling—convenience, purposive	•	•			•	•	•	•	•	•	•	•	•	•		•		•	•	•	•	•
Setting		•		•	•			•			•				•					•		
Characteristics and description of sample		•			•			•			•		•	•								
Reasons for participant selection	•	•				•		•			•											
Non-participation	•	•		•	•																	
Inclusion and exclusion, criteria		•			•	•													•			
Identity of the person responsible for recruitment				•	•						•				•							
Sample size		•		•	•						•										•	
Method of approach		•									•					•						
Description of explanation of research to participants	•				•										•							
Level and type of participation														•								
Method of data collection, e.g. focus group, in-depth interview	•	•	•	•	•	•		•	•		•	•	•	•		•				•	•	
Audio and visual recording	•	•	•	•	•	•				•	•		•						•		•	•
Transcripts			•	•	•	•			•		•		•						•			•
Setting and location	•	•		•	•		•		•		•				•						•	•
Saturation of data	•	•	•			•			•				•	•							•	
Use of a topic guide, tools, questions	•	•	•								•					•			•	•		
Field notes			•	•	•	•													•			•
Changes and modifications	•	•		•	•														•		•	
Duration of interview, focus group		•				•					•									•		
Sensitive to participant language and views		•										•		•								
Number of interviews, focus groups		•				•																
Time span																					•	
Time and resources available to the study		•																				

[a]Other publications, [b]Systematic review of qualitative studies; BMJ, British Medical Journal—editor's checklist for appraising qualitative research; •, item included in the checklist.

Downloaded from https://academic.oup.com/intqhc/article/19/6/349/1791966 by guest on 31 October 2021

Table 4 Items included in 22 published checklists: Analysis and reporting

Item	[26][a]	[27][a]	[6][b]	[28][b]	[32][b]	[13]	[15]	[14]	[17]	[33]	[34]	[35]	[16]	[19]	[36]	[7]	[37]	[23]	[38]	[39]	[22]	BMJ
Respondent validation	•	•	•		•		•		•	•			•	•			•	•	•	•		
Limitations and generalizability	•	•		•	•		•		•		•		•	•				•	•			
Triangulation	•	•		•	•	•	•	•	•					•			•		•			
Original data, quotation		•	•	•	•			•	•		•			•		•				•	•	•
Derivation of themes explicit	•	•	•	•	•		•	•			•								•			•
Contradictory, diverse, negative cases	•	•		•	•		•			•				•					•			•
Number of data analysts	•	•	•			•			•			•	•						•			•
In-depth description of analysis	•			•	•			•			•			•							•	•
Sufficient supporting data presented	•	•		•	•		•				•					•						
Data, interpretation and conclusions linked and integrated		•		•	•							•		•						•		
Retain context of data		•					•	•						•					•			
Explicit findings, presented clearly	•	•		•					•	•												
Outside checks													•	•				•	•			
Software used		•				•													•			•
Discussion both for and against the researchers' arguments	•	•		•	•																	
Development of theories, explanations		•					•			•		•										
Numerical data		•									•							•				•
Coding tree or coding system		•					•												•		•	
Inter-observer reliability		•									•										•	
Sufficient insight into meaning/perceptions of participants		•								•												
Reasons for selection of data to support findings		•			•																	
New insight		•						•														
Results interpreted in credible, innovative way									•													
Eliminate other theories													•									
Range of views														•								
Distinguish between researcher and participant voices								•														
Proportion of data taken into account														•								

[a]Other publications, [b]Systematic review of qualitative studies; BMJ, British Medical Journal—editor's checklist for appraising qualitative research, •, item included in the checklist.

Downloaded from https://academic.oup.com/intqhc/article/19/6/349/1791966 by guest on 31 October 2021

For peer review only

For peer review only - http://bmjopen.bmj.com/site/about/guidelines.xhtml

Researchers should report the sample size of their study to enable readers to assess the diversity of perspectives included.

(iii) Setting: Researchers should describe the context in which the data were collected because it illuminates why participants responded in a particular way. For instance, participants might be more reserved and feel disempowered talking in a hospital setting. The presence of non-participants during interviews or focus groups should be reported as this can also affect the opinions expressed by participants. For example, parent interviewees might be reluctant to talk on sensitive topics if their children are present. Participant characteristics, such as basic demographic data, should be reported so readers can consider the relevance of the findings and interpretations to their own situation. This also allows readers to assess whether perspectives from different groups were explored and compared, such as patients and health care providers [13, 19].

(iv) Data collection: The questions and prompts used in data collection should be provided to enhance the readers' understanding of the researcher's focus and to give readers the ability to assess whether participants were encouraged to openly convey their viewpoints. Researchers should also report whether repeat interviews were conducted as this can influence the rapport developed between the researcher and participants and affect the richness of data obtained. The method of recording the participants' words should be reported. Generally, audio recording and transcription more accurately reflect the participants' views than contemporaneous researcher notes, more so if participants checked their own transcript for accuracy [19–21]. Reasons for not audio recording should be provided. In addition, field notes maintain contextual details and non-verbal expressions for data analysis and interpretation [19, 22]. Duration of the interview or focus group should be reported as this affects the amount of data obtained. Researchers should also clarify whether participants were recruited until no new relevant knowledge was being obtained from new participants (data saturation) [23, 24].

Domain 3: analysis and findings

(i) Data analysis: Specifying the use of multiple coders or other methods of researcher triangulation can indicate a broader and more complex understanding of the phenomenon. The credibility of the findings can be assessed if the process of coding (selecting significant sections from participant statements), and the derivation and identification of themes are made explicit. Descriptions of coding and memoing demonstrate how the researchers perceived, examined and developed their understanding of the data [17, 19]. Researchers sometimes use software packages to assist with storage, searching and coding of qualitative data. In addition, obtaining feedback from participants on the research findings adds validity to the researcher's interpretations by ensuring that the participants' own meanings and perspectives are represented and not curtailed by the researchers' own agenda and knowledge [23].

(ii) Reporting: If supporting quotations are provided, researchers should include quotations from different participants to add transparency and trustworthiness to their findings and interpretations of the data [17]. Readers should be able to assess the consistency between the data presented and the study findings, including the both major and minor themes. Summary findings, interpretations and theories generated should be clearly presented in qualitative research publications.

Discussion

The COREQ checklist was developed to promote explicit and comprehensive reporting of qualitative studies (interviews and focus groups). The checklist consists of items specific to reporting qualitative studies and precludes generic criteria that are applicable to all types of research reports. COREQ is a comprehensive checklist that covers necessary components of study design, which should be reported. The criteria included in the checklist can help researchers to report important aspects of the research team, study methods, context of the study, findings, analysis and interpretations.

At present, we acknowledge there is no empiric basis that shows that the introduction of COREQ will improve the quality of reporting of qualitative research. However this is no different than when CONSORT, QUOROM and other reporting checklists were introduced. Subsequent research has shown that these checklists have improved the quality of reporting of study types relevant to each checklist [5, 25], and we believe that the effect of COREQ is likely to be similar. Despite differences in the objectives and methods of quantitative and qualitative methods, the underlying aim of transparency in research methods and, at the least, the theoretical possibility of the reader being able to duplicate the study methods should be the aims of both methodological approaches. There is a perception among research funding agencies, clinicians and policy makers, that qualitative research is 'second class' research. Initiatives like COREQ are designed to encourage improvement in the quality of reporting of qualitative studies, which will indirectly lead to improved conduct, and greater recognition of qualitative research as inherently equal scientific endeavor compared with quantitative research that is used to assess the quality and safety of health care. We invite readers to comment on COREQ to improve the checklist.

References

1. Moher D, Schulz KF, Altman D. The CONSORT statement: revised recommendations for improving the quality of reports of parallel-group randomized trials. *JAMA* 2001;**285**:1987–91.

2. Moher D, Cook DJ, Eastwood S *et al.* Improving the quality of reports of meta-analyses of randomised controlled trials: the QUOROM statement. Quality of Reporting of Meta-analyses. *Lancet* 1999;**354**:1896–900.

Downloaded from https://academic.oup.com/intqhc/article/19/6/349/1791966 by guest on 31 October 2021

For peer review only

3. STROBE Statement: Strengthening the reporting of observational studies in epidemiology. http://www.strobe-statement.org/Checkliste.html
4. Stroup DF, Berlin JA, Morton SC *et al.* Meta-analysis of observational studies in epidemiology: a proposal for reporting. Meta-analysis Of Observational Studies in Epidemiology (MOOSE) group. *JAMA* 2000;**283**:2008–12.
5. Moher D, Jones A, Lepage L. Use of the CONSORT Statement and quality of reports of randomized trials. A comparative before-and-after evaluation. *JAMA* 2001;**285**:1992–5.
6. Mills E, Jadad AR, Ross C *et al.* Systematic review of qualitative studies exploring parental beliefs and attitudes toward childhood vaccination identified common barriers to vaccination. *J Clin Epidemiol* 2005;**58**:1081–8.
7. Knafl KA, Howard NJ. Interpreting and reporting qualitative research. *Res Nurs Health* 1984;**7**:7–14.
8. Sofaer S. Qualitative research methods. *Int J Qual Health Care* 2002;**14**:329–36.
9. Wright EB, Holcombe C, Salmon P. Doctor's communication of trust, care, and respect in breast cancer: qualitative study. *BMJ* 2004;**328**:864–8.
10. Liamputtong P, Ezzy D. *Qualitative Research Methods*. 2nd edn. Melbourne, Victoria: Oxford University Press, 2005.
11. Krueger RA, Casey MA. *Focus Groups. A Practical Guide for Applied Research.* Thousand Oaks CA: Sage Publications, 2000.
12. Morgan DL. *Focus Groups as Qualitative Research.* Newbury Park, California: Sage, 1988.
13. Giacomini MK, Cook DJ. Users' guides to the medical literature XXIII. Qualitative research in health care. A. Are the results of the study valid? *JAMA* 2000;**284**:357–62.
14. Malterud K. Qualitative research:standards challenges guidelines. *Lancet* 2001;**358**:483–8.
15. Mays N, Pope C. Qualitative research in health care: assessing quality in qualitative research. *BMJ* 2000;**320**:50–2.
16. Elder NC, William L. Reading and evaluating qualitative research studies. *J Fam Pract* 1995;**41**:279–85.
17. Cote L, Turgeon J. Appraising qualitative research articles in medicine and medical education. *Med Teach* 2005;**27**:71–5.
18. Altheide D, Johnson J. Criteria for assessing interpretive validity in qualitative research. In Denzin N, Lincoln Y (eds). *Handbook of Qualitative Research.* Thousand Oaks CA: Sage Publications, 1994.
19. Fossey E, Harvey C, McDermott F *et al.* Understanding and evaluating qualitative research. *Aust N Z J Psychiatry* 2002;**36**:717–32.
20. Seale C, Silverman S. Ensuring rigour in qualitative research. *Eur J Public Health* 1997;**7**:379–84.
21. Scheff T. Single case analysis in the health sciences. *Eur J Public Health* 1995;**5**:72–4.
22. Bluff R. Evaluating qualitative research. *Br J Midwifery* 1997;**5**:232–5.
23. Popay J, Rogers A, Williams G. Rationale and standards for the systematic review of qualitative literature in health services research. *Qual Health Res* 1998;**8**:341–51.
24. Blumer H. *Critiques of Research, in the Social Sciences.* New Brunswick, NJ: Transaction Books, 1979.
25. Delaney A, Bagshaw SM, Ferland A *et al.* A systematic evaluation of the quality of meta-anlyses in the critical care literature. *Crit Care* 2005;**9**:575–82.
26. Critical Skills Appraisal Programme (CASP) 10 Questions to help you make sense of qualitative research: Milton Keynes Primary Care Trust, 2002.
27. Spencer L, Ritchie J, Lewis J *et al. Quality in Qualitative Evaluation: A Framework for Assessing Research Evidence.* London: Cabinet Office. Government Chief Social Researcher's Office, 2003.
28. Campbell R, Pound P, Pope C *et al.* Evaluating meta-ethnography: a synthesis of qualitative research on lay experience of diabetes and diabetes care. *Soc Sci Med* 2003;**56**:671–84.
29. Feder GS, Hutson M, Ramsay I *et al.* Women exposed to intimate partner violence: expectations and experiences when they encounter health care professionals: a meta-analysis of qualitative studies. *Arch Intern Med* 2006;**166**:22–37.
30. Pound P, Britten N, Morgan M *et al.* Resisting medicines: a synthesis of qualitative studies of medicine taking. *Soc Sci Med* 2005;**61**:133–55.
31. Smith LK, Pope C, Botha JL. Patients' help-seeking experiences and delay in cancer presentation: a qualitative synthesis. *Lancet* 2005;**366**:825–31.
32. Walter FM, Emery J, Braithwaite D *et al.* Lay understanding of familial risk of common chronic diseases: a systematic review and synthesis of qualitative research. *Ann Fam Med* 2004; **2**:583–94.
33. Inui TS, Frankel RM. Evaluating the quality of qualitative research: a proposal pro-term. *J Gen Intern Med* 1991;**6**:485–6.
34. Boulton M, Fitzpatrick R, Swinburn C. Qualitative research in health care: II A structured review and evaluation of studies. *J Eval Clin Pract* 1996;**2**:171–9.
35. Dixon-Woods M, Shaw RL, Agarwal S *et al.* The problem of appraising qualitative research. *Qual Saf Health Care* 2004;**13**:223–5.
36. Hoddinott P, Pill R. A review of recently published qualitative research in general practice. More methodological questions than answers? *Fam Pract* 1997;**14**:313–9.
37. Kuzel AJ, Engel JD, Addison RB *et al.* Desirable features of qualitative research. *Fam Pract Res J* 1994;**14**:369–78.
38. Treloar C, Champness S, Simpson PL *et al.* Critical appraisal checklist for qualitative research studies. *Indian J Pediatr* 2000;**67**:347–51.
39. Cesario S, Morin K, Santa-Donato A. Evaluating the level of evidence in qualitative research. *J Obstet Gynecol Neonatal Nurs* 2001;**31**:708–14.

Accepted for publication 7 July 2007

For peer review only

Downloaded from https://academic.oup.com/intqhc/article/19/6/349/1791966 by guest on 31 October 2021
